# Design, Synthesis and Biological Evaluation of New Antioxidant and Neuroprotective Multitarget Directed Ligands Able to Block Calcium Channels

**DOI:** 10.3390/molecules25061329

**Published:** 2020-03-14

**Authors:** Irene Pachòn Angona, Solene Daniel, Helene Martin, Alexandre Bonet, Artur Wnorowski, Maciej Maj, Krzysztof Jóźwiak, Tiago Barros Silva, Bernard Refouvelet, Fernanda Borges, José Marco-Contelles, Lhassane Ismaili

**Affiliations:** 1 Neurosciences Intégratives et Cliniques EA 481, Pôle de Chimie Organique et Thérapeutique, Univ. Bourgogne Franche-Comté, UFR Santé, 19, rue Ambroise Paré, F-25000 Besançon, France; pachon.angona.irene@gmail.com (I.P.A.); solene.du70@hotmail.fr (S.D.); bernard.refouvelet@univ-fcomte.fr (B.R.); 2 PEPITE EA4267, Laboratoire de Toxicologie Cellulaire, Univ. Bourgogne Franche-Comté, F-25000 Besançon, France; helene.martin@univ-fcomte.fr (H.M.); alexandre.bonet@univ-fcomte.fr (A.B.); 3 Department of Biopharmacy, Medical University of Lublin, ul. W. Chodzki 4a, 20-093 Lublin, Poland; artur.wnorowski@gmail.com (A.W.); maciejmaj@umlub.pl (M.M.); krzysztof.jozwiak@umlub.pl (K.J.); 4 CIQUP/Department of Chemistry and Biochemistry, Faculty of Sciences, University of Porto, R. Campo Alegre 1021/1055, 4169-007 Porto, Portugal; ntb.silva@gmail.com (T.B.S.); mfernandamborges@gmail.com (F.B.); 5 Laboratory of Medicinal Chemistry (IQOG, CSIC), Juan de la Cierva, 3, 28006 Madrid, Spain

**Keywords:** Alzheimer’s disease, Ca^2+^ channel antagonists, Hantzsch reaction, multitarget directed ligands, neuroprotection, oxidative stress

## Abstract

We report herein the design, synthesis and biological evaluation of new antioxidant and neuroprotective multitarget directed ligands (MTDLs) able to block Ca^2+^ channels. New dialkyl 2,6-dimethyl-4-(4-(prop-2-yn-1-yloxy)phenyl)-1,4-dihydropyridine-3,5-dicarboxylate MTDLs **3a**–**t**, resulting from the juxtaposition of nimodipine, a Ca^2+^ channel antagonist, and rasagiline, a known MAO inhibitor, have been obtained from appropriate and commercially available precursors using a Hantzsch reaction. Pertinent biological analysis has prompted us to identify the MTDL 3,5-dimethyl-2,6–dimethyl–4-[4-(prop–2–yn–1-yloxy)phenyl]-1,4-dihydro- pyridine- 3,5-dicarboxylate (**3a**), as an attractive antioxidant (1.75 TE), Ca^2+^ channel antagonist (46.95% at 10 μM), showing significant neuroprotection (38%) against H_2_O_2_ at 10 μM, being considered thus a hit-compound for further investigation in our search for anti-Alzheimer’s disease agents.

## 1. Introduction

Alzheimer’s disease (AD) is a neurodegenerative pathology characterized by a highly interconnected biological processes leading to neuronal death, accumulation and aggregation of abnormal extracellular deposits of beta-amyloid peptide (Aβ) and neurofibrillary tangles, composed of hyperphosphorylated tau protein [1], and low level of neurotransmitter acetylcholine. Therefore, new strategies, based on the multitarget directed ligand (MTDL) approach [2,3], have been developed for the design of new drugs able to bind simultaneously at diverse enzymatic systems or receptors involved in the progress of AD [4,5,6,7,8]. Accordingly, and following this paradigm a number of MTDLs has been described by many research groups [9,10,11]. Our contributions in this area have used multicomponent reactions (MCRs) [12,13,14,15,16] as the method of choice for introducing rapidly and efficiently chemical diversity in the search for new MTDLs.

In this context, we report herein the design, synthesis and biological evaluation of new MTDLs able to lower the oxidative stress (OS), inhibit monoamine oxidase enzymes (MAOs), and block Ca^2+^ channels. OS plays a crucial role in the pathogeny of AD [17,18]. Diverse molecular processes converge to generate high concentrations of radical oxygenated species (ROS) that oxidize proteins, nucleic acids, polysaccharides and lipids, whose modification may affect their structure and biological functions resulting in the neuronal death [19]. OS is caused by various underlying factors, such as mitochondrial dysfunction [20,21], and disruption of metal homeostasis (Cu, Fe, Zn), being also implicated in Aβ aggregation [18,22] and neuroinflammation [23,24]. Therefore, the antioxidant strategy is one of the most promising pathways for the development of new drugs for aging-related diseases, such as AD [25]. MAO is an interesting pharmacological target for the development of new drugs for AD and other neurodegenerative diseases, such as Parkinson’s disease (PD). In fact, MAO catalyze the deamination of biogenic amines such adrenaline, dopamine, serotonin, thus leading to the production of H_2_O_2_, which is a source of ROS, responsible of OS [26,27]. Ca^2+^ channel blockade is also a well-established pathway in the development of new drugs for AD, as evidenced by the example of nivaldipine, currently under clinical development [28]. Ca^2+^ entry through voltage-gated L-type Ca^2+^ channels (Ca_v_ 1.1–1.4) causes both Ca^2+^ overload and mitochondrial disruption, which leads to the activation of the apoptotic cascade and cell death [29]. In addition, increased cytosolic calcium levels, involved in the pathogenesis of AD, regulate glycogen synthase kinase, protein kinase C, and other kinases that hyperphosphorylate tau, potentiate NFT formation [30] and also facilitate the formation of A*β* peptides through calcium-mediated *β*-secretase activity [31,32,33,34,35].

**Figure 1:**
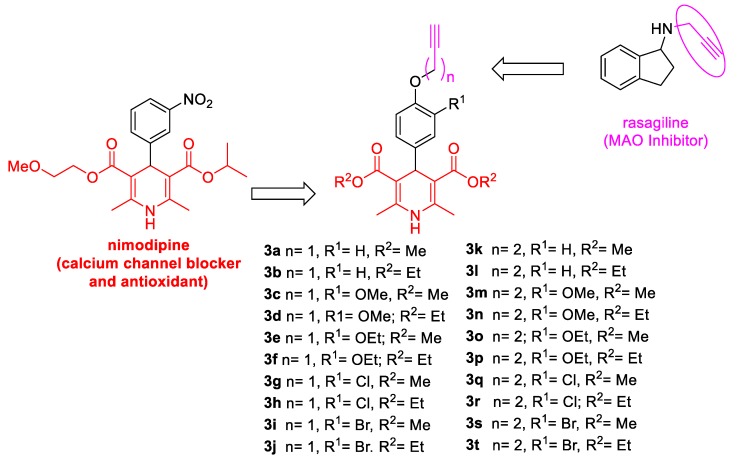
Structure of nimodipine, rasagiline, and the new MTDL dialkyl 2,6-dimethyl-4-(4-(prop-2-yn-1-yloxy)phenyl)-1,4-dihydropyridine-3,5-dicarboxylate derivatives **3a**–**t** reported here.

The new MTDLs **3a**–**t** reported here are dialkyl 2,6-dimethyl-4-(4-(prop-2-yn-1-yloxy)- phenyl)-1,4-dihydropyridine-3,5-dicarboxylate derivatives (Figure 1), that result from the juxtaposition of nimodipine, a Ca^2+^ channel blocker, and rasagiline, a known MAO inhibitor. Thus, the designed MTDLs bear a 1,4-DHP, the core fragment of well-known Ca^2+^ channel antagonists, attached to a propargylalkoxy motif, a well-known MAO pharmacophore, able to promote also neuronal survival via neuroprotective/ neurorescue pathways [36,37].

## 2. Results and Discussion

### 2.1. Synthesis

**Scheme 1:**
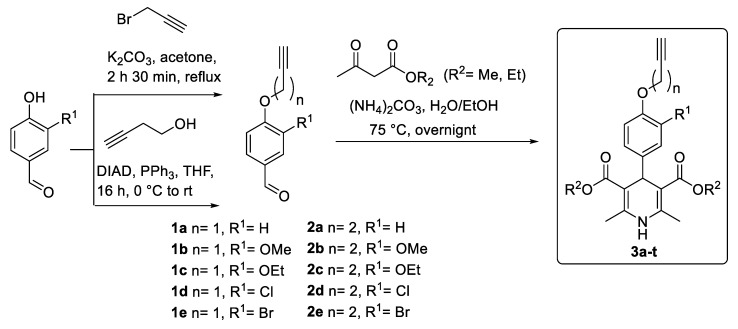
Synthesis of dialkyl 2,6-dimethyl-4-(4-(alk-2-yn-1-yloxy)phenyl)-1,4-dihydropyridine-3,5-dicarboxylates **3a**–**t**.

The synthesis of the new MTDL **3a**–**t** has been carried via a one-pot Hantzsch reaction of aldehydes **1a**–**e**/**2a**–**e** with ethyl or methyl acetoacetate and ammonium carbonate in EtOH/water (Scheme 1). Aldehydes **1a**–**e** were prepared from the appropriate substituted 4-hydroxybenzaldehydes and propargyl bromide, under typical Williamson reaction conditions (Scheme 1). Aldehydes **2a**–**e** were synthesized by a Mitsunobu reaction under the conditions described by Mertens [38], from but-3-yn-1-ol and 3-substituted 4-hydroxybenzaldehydes, in the presence of Ph_3_P and diisopropyl azodicarboxylate (DIAD), in THF, at room temperature (rt) (Scheme 1). All new compounds showed excellent analytical and spectroscopic data, in good agreement with the expected values (see Material and Methods, and Supplementary Material).

To verify, the effectiveness of our design, compounds **3a**–**t** were submitted to Ca^2+^ channel blockade, antioxidant and MAO inhibition evaluation assays, followed by the neuroprotection analysis of selected compounds.

### 2.2. Biological Evaluation

#### 2.2.1. Ca^2+^ Channel Blockade

The Ca^2+^ channel blockade capacity of compounds **3a-t**, and nimodipine as standard, at 10 μM concentration, has been carried out following the usual methodology [39]. As shown in Table 1, the observed % values ranged from 20.2 (**3k**) to 47.0 (**3a**). The most potent DHP corresponded, in decreasing order, to **3a** (47.0 ± 6.6%), **3h** (42.8 ± 14.0%), and **3j** (39.0 ± 4.8%), comparing thus very favorably witH-Nimodipine (52.8 ± 5.5%). From the point of view of the structure activity relationship (SAR), compounds bearing n = 1 length as linker showed better results than those bearing *n* = 2 linkers. In fact, only one adduct with n = 1 presented an inhibition value under 25% (**3f,** 22.9 ± 6.7**%**), the rest surpassed 30% Ca^2+^ influx blockage, which is 2/3-fold the value of nimodipine (52.8%). Concerning the influence of R^1^ over the blockade activity, no conclusions can be drawn. However, it is worth mentioning that compounds **3a** and **3h** with R^1^ = H and Cl, respectively, are the most active compounds, as they presented values of inhibition that almost doubled the rest (47% and 43%).

#### 2.2.2. Antioxidant Assay

The antioxidant activity of compounds **3a**–**t**, compared to melatonin, used as positive control, showing an ORAC value of 2.45 [12], was determined by the ORAC-FL method [40]. The antioxidant activities are expressed as Trolox equivalents (TE) units. As shown in Table 1, the values for the antioxidant capacity range from 0.52 (**3e**) to 2.78 (**3n**). Three compounds, **3l** (2.45 TE), **3n** (2.78 TE) and **3p** (2.78 TE), showed antioxidant activities equal or higher than melatonin (2.45 TE). Concerning the SAR, for the same R^1^ substituent, compounds with a linker length of *n* = 2 showed better ORAC values than those with *n* = 1, except for the pairs **3g, 3q** and **3h, 3r.** For the same linker length the best results for *n* = 1 were obtained for compounds bearing R^1^ = H, whereas for compounds with *n* = 2, the best results corresponded to molecules bearing R^1^ = OMe or R^1^ = OEt.

#### 2.2.3. hMAOs Inhibition

The effect of the compounds **3a**–**t** on the activity of both human MAO (hMAO) isoforms was evaluated by measuring the production of 4-hydroxyquinoline (4-HQ, λmax = 316 nm) from kynuramine, using microsomal recombinant hMAO isoforms. Unexpectedly and unfortunately these compounds showed a very low inhibition.

Based on the previously described biological results, the three most balanced compounds (**3a**, **3h** and **3j**) against calcium channel blockade and antioxidant activity were evaluated for their capacity to protect human neuronal cells (SH-SY5Y cell line) from cell death.

#### 2.2.4. Neuroprotective Activity

Several in vitro approaches have been performed to mimic human neuronal features, based on neuronal-like cells such as the neuroblastoma line SH-SY5Y, a human cell line that divides quickly and has the ability to differentiate in post-mitotiC-Neurons, thus it is considered a convenient and popular model to study neuroprotective activity for PD and AD [41]. For this purpose, cytotoxicity was induced by mitochondrial respiratory chain blockers oligomycin rotenone (O/R) and by H_2_O_2_, a well-known toxic responsible for the generation of ROS. Prior to the neuroprotective assay, the effect of the compounds on the cell viability was evaluated at 1 and 10 µM, showing no cytotoxicity against SH-SY5Y cells. As shown in Table 2, compounds **3a** and **3j** showed a modest neuroprotective effect against O/R. However, and very interestingly, the two compounds showed an interesting effect against H_2_O_2_, particularly at 10 µM where they showed a percentage of neuroprotection equal to 38 and 39 for **3a** and **3j**, respectively.

## 3. Materials and Methods

### 3.1. General Information

All reagents were purchased from Sigma Aldrich (Saint-Quentin Fallavier France) or TCI (Zwijndrecht, Belgium). ^1^H- and ^13^C-NMR spectra were recorded on a Bruker (Wissembourg France) spectrometer, operating at 400 and 100 MHz, respectively, in solution in dimethylsulfoxide (DMSO-d_6_) at rt. Chemical shift values are given in δ (ppm) relatively to TMS as internal reference. Coupling constants are given in Hz. The following abbreviations were used: s= singlet, d= doublet, t= triplet, q= quartet, m= multiplet. Elemental analyses were obtained by a Carlo Erba EA 1108 analyzer and the analytical results were within ± 0.2% of the theoretical values for all compounds. High resolution mass spectra were obtained at Centre Commun de Spectrométrie de Masse, Lyon, France on a Bruker micrOTOF-Q II spectrometer (Bruker Daltonics, Champs sur Marne France) in positive ESI-TOF (electrospray ionization-time of flight).

### 3.2. Synthesis of Propargylic Aldehydes ***1a–e***

A suspension of the corresponding 4-hydroxybenzaldehyde (1 equiv) and K_2_CO_3_ (1.3 equiv) in acetone (1.6 mmol/mL) was stirred at reflux for 30 min. The mixture was cooled to rt and propargyl bromide (1.6 equiv) was added dropwise. The resulting suspension was stirred at reflux for 2 h 30 min. After that time, the solvent was removed under pressure conditions. The residue was dissolved in water and extracted with ethyl acetate three times. Organic layers were joined and dried over Na_2_SO_4_. Activated carbon was added to the solution and the mixture is stirred over 15 min at 40 °C. The crude was finally filtered over Celite^®^, the filtrate was evaporated and the obtained residue was recrystallized from EtOAct/hexane (1:2 *v*/*v*) to afford the desired products in yields ranging from 34% to 98%.

*4-(Prop-2-yn-1-yloxy)benzaldehyde* (**1a**). The crude was prepared according to the general procedure starting from commercially available 4-hydroxybenzaldehyde (1 equiv, 8.19 mmol, 1 g), K_2_CO_3_ (1.3 equiv, 10.65 mmol, 1.47 g) and propargyl bromide (1.6 equiv, 13.10 mmol, 0.992 mL) in acetone (20 mL) to afford compound **1a** (1.00 g, 77%). The crude was used without further purification. ^1^H-NMR (CDCl_3_) δ 9.91 (s, 1H), 7.93–7.80 (m, 2H), 7.14–7.07 (m, 2H), 4.78 (d, *J* = 2.4 Hz, 2H), 2.57 (t, *J* = 2.4 Hz, 1H).

*3-Methoxy-4-(prop-2-yn-1-yloxy)benzaldehyde* (**1b**). The crude was prepared according to the general procedure starting from commercially available 4-hydroxy-3-methoxybenzaldehyde (1 equiv, 6.58 mmol, 1 g), K_2_CO_3_ (1.3 equiv, 8.55 mmol, 1.181 g) and propargyl bromide (1.6 equiv, 10.52 mmol, 0.800 mL) in acetone (16 mL) to afford compound **1b** (1.16 g, 93%). The crude was used without further purification. ^1^H-NMR (CDCl_3_) δ 9.87 (s, 1H), 7.52–7.39 (m, 2H), 7.14 (d, *J* = 8.1 Hz, 1H), 4.86 (d, *J* = 2.3 Hz, 2H), 3.94 (s, 3H), 2.56 (t, *J* = 2.4 Hz, 1H).

*3-Ethoxy-4-(prop-2-yn-1-yloxy)benzaldehyde* (**1c**). The crude was prepared according to the general procedure starting from commercially available 3-ethoxy-4-hydroxybenzaldehyde (1 equiv, 6.02 mmol, 1.00 g), K_2_CO_3_ (1.3 equiv, 7.82 mmol, 1.081 g) and propargyl bromide (1.6 equiv, 9.63 mmol, 0.730 mL) in acetone (15 mL) to afford compound **1c** (428.32 mg, 35%). The crude was used without further purification.

*3-Chloro-4-(prop-2-yn-1-yloxy)benzaldehyde* (**1d**). The crude was prepared according to the general procedure starting from commercially available 3-chloro-4-hydroxybenzaldehyde (1 equiv, 6.39 mmol, 1.00 g), K_2_CO_3_ (1.3 equiv, 8.31 mmol, 1.148 g) and propargyl bromide (1.6 equiv, 10.22 mmol, 0.78 mL) in acetone (10 mL) to afford compound **1d** (852.80 mg, 69%). The crude was used without further purification. ^1^H-NMR (CDCl_3_) δ 9.87 (s, 1H), 7.93 (d, *J* = 2.0 Hz, 1H), 7.79 (dd, *J* = 8.5, 2.0 Hz, 1H), 7.21 (d, *J* = 8.5 Hz, 1H), 4.88 (d, *J* = 2.4 Hz, 2H), 2.60 (t, *J* = 2.4 Hz, 1H).

*3-Bromo-4-(prop-2-yn-1-yloxy)benzaldehyde* (**1e**). The crude was prepared according to the general procedure starting from commercially available 3-bromo-4-hydroxybenzaldehyde (1 equiv, 4.98 mmol, 1.00 g), K_2_CO_3_ (1.3 equiv, 6.47 mmol, 893.81 mg) and propargyl bromide (1.6 equiv, 7.96 mmol, 0.60 mL) in acetone (12 mL) to afford compound **1e** (1.163 g 98%). The crude was used without further purification. ^1^H-NMR (CDCl_3_) δ 9.86 (s, 1H), 8.09 (d, *J* = 1.0 Hz, 1H), 7.82 (dd, *J* = 8.5, 1.1 Hz, 1H), 7.17 (d, *J* = 8.5 Hz, 1H), 4.89–4.81 (m, 2H), 2.60 (t, *J* = 2.4 Hz, 1H).

### 3.3. Synthesis of Propargylic Aldehydes 2a–e

A solution of 4-hydroxybenzaldehyde (1 equiv), triphenylphosphine (2 equiv) and 3-butyn-1-ol (1.5 equiv) in THF (0.8 mmol/mL) was cooled to 0 °C. DIAD (1.5 equiv) is then added dropwise and the resulting mixture is stirred overnight at rt. The solvent was evaporated, the residue solubilized in ethyl acetate and washed 3 times with 1M NaOH solution and brine. The organic layers were dried over Na_2_SO_4_, filtered and evaporated under pressure conditions. The residue was triturated with ethyl ether and filtered. The filtrate was purified by flash chromatography with hexane/EtOAc (9:1 *v*/*v*) to afford the desired products in yields ranging from 45% to 97%.

*4-(But-3-yn-1-yloxy)benzaldehyde* (**2a**). The crude was prepared according to the general procedure starting from commercially available 4-hydroxybenzaldehyde (1 equiv, 16.38 mmol, 2.00 g), triphenylphosphine (2 equiv, 32.76 mmol, 8.59 g), DIAD (1.5 equiv, 24.57 mmol, 4.84 mL) and 3-butyn-1-ol (1.5 equiv, 27.57 mmol, 1.860 mL) in THF (120 mL) to afford compound **2a** (1.30 g, 46%). The crude was used without further purification. ^1^H-NMR (400 MHz, CDCl_3_): δ 2.06 (br s, 1 H, CH), 2.73 (dt, *J* = 2.4, 7.1 Hz, 2 H, CH_2_), 4.18 (t, *J* = 7.1 Hz, 2 H, CH_2_O), 7.02 (d, *J* = 8.5 Hz, 2 H, H*arom*), 7.84 (d, *J* = 8.5 Hz, 2 H, H*arom*), 9.90 (s, 1 H, CHO).

*4-(But-3-yn-1-yloxy)-3-methoxybenzaldehyde* (**2b**). The crude was prepared according to the general procedure starting from commercially available 4-hydroxy-3-methoxybenzaldehyde (1 equiv, 6.57 mmol, 1.00 g), triphenylphosphine (2 equiv, 13.15 mmol, 3.45 g), DIAD (1.5 equiv, 9.86 mmol, 1.94 mL) and 3-butyn-1-ol (1.5 equiv, 9.86 mmol, 0.746 mL) in THF (60 mL) to afford compound **2b** (674.9 mg, 50%). The crude was used without further purification. ^1^H-NMR (CDCl_3_) δ 9.85 (s, *J* = 4.2 Hz, 1H), 7.48–7.38 (m, 2H), 6.99 (dd, *J* = 8.1, 3.5 Hz, 1H), 4.28–4.18 (m, 2H), 3.97–3.89 (m, 3H), 2.77 (td, *J* = 7.3, 2.7 Hz, 2H), 2.09–2.00 (m, 1H).

*4-(But-3-yn-1-yloxy)-3-ethoxybenzaldehyde* (**2c**). The crude was prepared according to the general procedure starting from commercially available 3-ethoxy-4-hydroxybenzaldehyde (1 equiv, 6.02 mmol, 1.00 g), triphenylphosphine (2 equiv, 12.04 mmol, 3.16 g), DIAD (1.5 equiv, 9.03 mmol, 1.78 mL) and 3-butyn-1-ol (1.5 equiv, 9.03 mmol, 0.683 mL) in THF (60 mL) to afford compound **2c** (1.423 g, 70%). The crude was used without further purification. ^1^H-NMR (CDCl_3_) δ 9.85 (s, 1H), 7.42 (dt, *J* = 5.5, 1.8 Hz, 2H), 6.99 (d, *J* = 8.0 Hz, 1H), 4.23 (t, *J* = 7.3 Hz, 2H), 4.15 (q, *J* = 7.0 Hz, 2H), 2.77 (td, *J* = 7.3, 2.7 Hz, 2H), 2.05 (t, *J* = 2.7 Hz, 1H), 1.47 (t, *J* = 7.0 Hz, 3H), 1.26 (d, *J* = 6.3 Hz, 3H).

*4-(But-3-yn-1-yloxy)-3-chlorobenzaldehyde* (**2d*)***. The crude was prepared according to the general procedure starting from commercially available 3-ethoxy-4-hydroxybenzaldehyde (1 equiv, 6.39 mmol, 1.00 g), triphenylphosphine (2 equiv, 12.78 mmol, 3.35 g), DIAD (1.5 equiv, 9.58 mmol, 1.89 mL) and 3-butyn-1-ol (1.5 equiv, 9.58 mmol, 0.725 mL) in THF (60 mL) to afford compound **2d** (1.30 g, 97%). The crude was used without further purification. ^1^H-NMR (CDCl_3_) δ 9.84 (s, 1H), 7.90 (d, *J* = 2.0 Hz, 1H), 7.76 (dd, *J* = 8.5, 2.0 Hz, 1H), 7.04 (d, *J* = 8.5 Hz, 1H), 4.26 (t, *J* = 7.1 Hz, 2H), 2.78 (td, *J* = 7.1, 2.7 Hz, 2H), 2.06 (t, *J* = 2.7 Hz, 1H).

*3-Bromo-4-(but-3-yn-1-yloxy)benzaldehyde* (**2e**). The crude was prepared according to the general procedure starting from commercially available 3-bromo-4-hydroxybenzaldehyde (1 equiv, 4.97 mmol, 1.00 g), triphenylphosphine (2 equiv, 9.94 mmol, 2.61 g), DIAD (1.5 equiv, 7.46 mmol, 1.47 mL) and 3-butyn-1-ol (1.5 equiv, 7.46 mmol, 0.565 mL) in THF (60 mL) to afford compound **2e** (631.20 mg, 50%). The crude was used without further purification. ^1^H-NMR (CDCl_3_) δ 9.85 (s, 1H), 8.09 (d, *J* = 2.0 Hz, 1H), 7.81 (dd, *J* = 8.5, 2.0 Hz, 1H), 7.00 (d, *J* = 8.5 Hz, 1H), 4.25 (t, *J* = 7.1 Hz, 2H), 2.80 (td, *J* = 7.1, 2.7 Hz, 2H), 2.07 (t, *J* = 2.7 Hz, 1H).

### 3.4. General procedure of compounds 3a–t

3-Substitued-4-alkynyloxy-benzaldehydes **1a**–**e** and **2a**–**e** (1 equiv) and the corresponding acetoacetate (3.5 equiv) were dissolved in a mixture of EtOH (4 mmol/mL) and the same volume of H_2_O. The resulting mixture is stirred and heated at 75 °C for 1 h. Next, ammonium carbonate (2.5 equiv) was added to the mixture, and the reaction stirred and heated at 75 °C overnight. The desired product precipitated once the crude reaction reached rt, or was triturated with diethyl ether. The solid was then filtered and washed with diethyl ether again to finally afford compounds **3a**–**t** in yields ranging from 12% to 76%.

**Figure 2:**
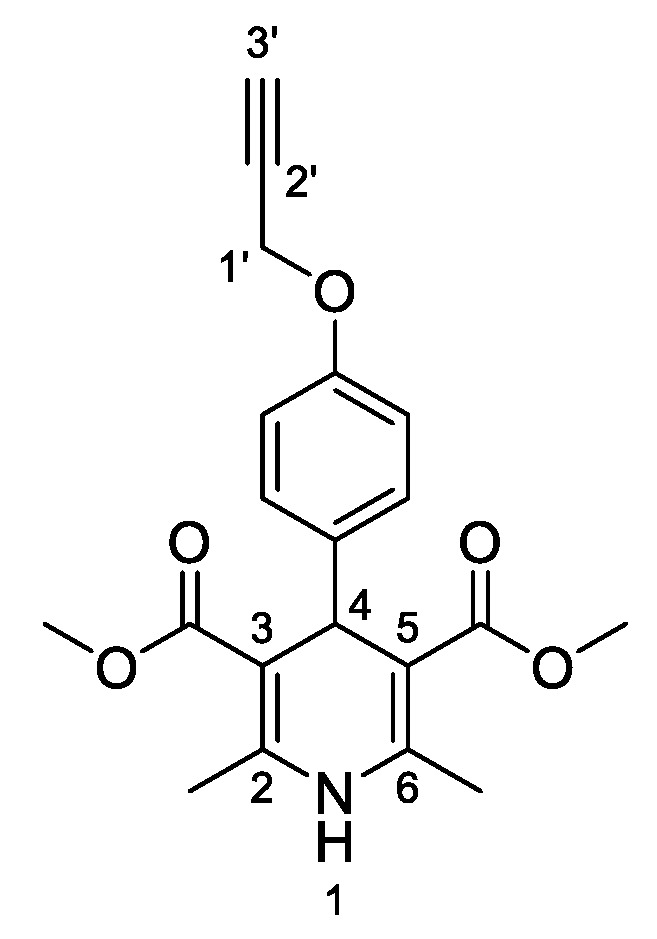
Chemical stucture of **3a**.

*3,5-Dimethyl-2,6–dimethyl–4-[4-(prop–2–yn–1-yloxy)phenyl]-1,4-dihydropyridine-3,5-dicarboxylate* (**3a,** Figure 2). Following the general procedure starting from 4-(prop-2-yn-1-yloxy)benzaldehyde (**1a**, 2.25 mmol, 360.02 mg), methyl acetoacetate (3.5 equiv, 7.88 mmol, 0.849 mL) and ammonium carbonate (2.5 equiv, 5.63 mmol, 540.51 mg) at 75 °C over 15 h, compound **3a** (509.4 mg, 64%) was isolated as white powder: ^1^H-NMR (CDCl_3_) δ 7.18 (d, *J* = 8.7 Hz, 2H, *2H_Ar_*), 6.82 (d, *J* = 8.7 Hz, 2H, 2*H_Ar_*), 5.67 (s, 1H, N*H*), 4.95 (s, 1H, *H_4_*), 4.62 (d, *J* = 2.4 Hz, 2H, *2H_1’_*), 3.64 (s, 6H, *2*CO_2_C*H_3_*), 2.50 (t, *J* = 2.4 Hz, 1H, *H_3’_*), 2.32 (s, 6H, 2C*H_3_*). ^13^C-NMR (CDCl_3_) δ 168.20, 156.19, 144.14, 140.94, 128.98, 128.79, 114.45, 114.37, 104.17, 79.03, 75.42, 55.91, 51.13, 38.58, 19.74. Anal. Calcd. for C_20_H_21_NO_5_: C, 67.59; H, 5.96; N, 3.94. Found: C, 67.33; H, 6.04; N, 3.89.

**Figure 3:**
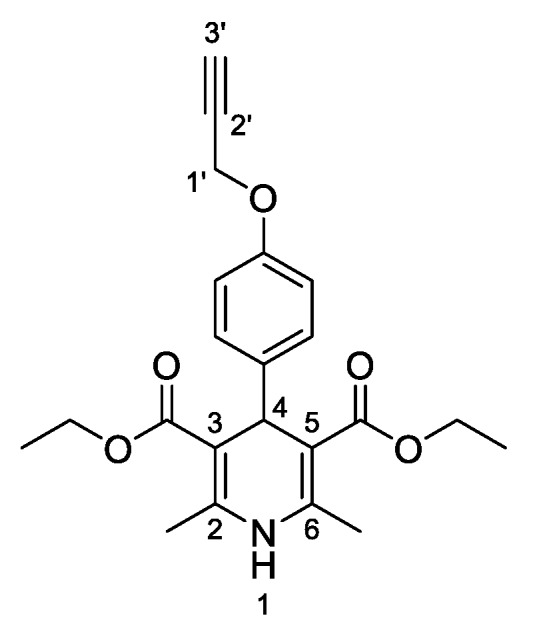
Chemical stucture of **3b**.

*3,5-Diethyl-2,6-dimethyl-4-[4-(prop-2-yn-1-yloxy)phenyl]-1,4-dihydropyridine-3,5-dicarboxylate* (**3b**, Figure 3). Following the general procedure starting from 4-(prop-2-yn-1-yloxy)benzaldehyde (**1a**, 1 equiv, 2.25 mmol, 360.02 mg), ethyl acetoacetate (3.5 equiv, 7.88 mmol, 1.00 mL) and ammonium carbonate (2.5 equiv, 5.63 mmol, 540.51 mg) at 75 °C over 15 h, the solvent was reduced under pressure conditions and the residue was triturated with diethyl ether. The resulting solid was stirred in diethyl ether overnight and filtered to afford compound **3b** (564.10 mg, 47%) as white powder: ^1^H-NMR (CDCl_3_) δ 7.23 – 7.12 (m, 2H, *2H_Ar_*), 6.86 – 6.75 (m, 2H, *2H_Ar_*), 5.58 (bs, 1H, N*H*), 4.94 (s, 1H, *H_4_*), 4.63 (d, *J* = 2.4 Hz, 2H, *2H_1’_*), 4.17 – 3.99 (m, 4H, 2OC*H**_2_***CH_3_), 2.49 (t, *J* = 2.4 Hz, 1H, *H_3’_*), 2.32 (s, 6H, 2C*H_3_*), 1.22 (t, *J* = 7.1 Hz, 6H, *2C*O_2_CH_2_C*H**_3_***). ^13^C-NMR (101 MHz, CDCl_3_) δ 167.79, 156.12, 143.72, 141.36, 129.17, 114.29, 104.49, 79.04, 75.38, 59.85, 56.00, 55.94, 38.94, 19.76, 14.41. HRMS ESI-TOF [M]^+^ *m/z* Calcd. for C_22_H_25_NO_5_: 383,1726. Found: 383,1733.

**Figure 4:**
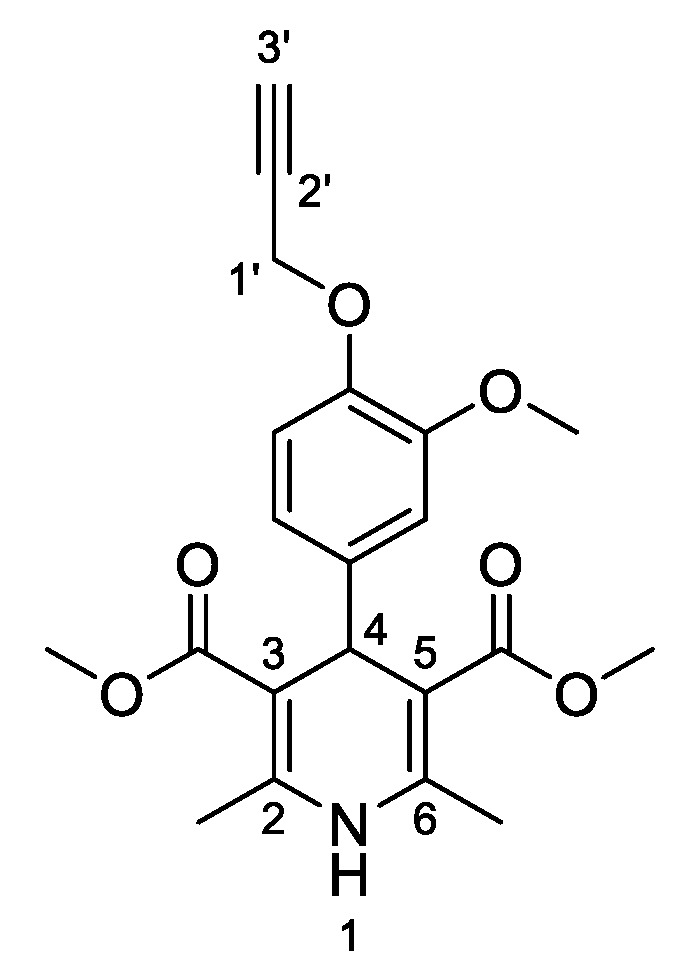
Chemical stucture of **3c**.

*3,5-Dimethyl-4- [3-methoxy-4-(prop-2-yn-1-yloxy) phenyl]-2,6-dimethyl-1,4-dihydropyridine-3,5- dicarboxylate* (**3c**, Figure 4). Following the general procedure starting from 3-methoxy-4-(prop-2-yn-1-yloxy)benzaldehyde (**1b**, 1 equiv, 1.84 mmol, 350.00 mg), methyl acetoacetate (3.5 equiv, 6.44 mmol, 0.695 mL) and ammonium carbonate (2.5 equiv, 4.60 mmol, 442.01 mg) at 75 °C over 15 h, compound **3c** (324.10 mg, 46%) precipitated as beige crystals: ^1^H-NMR (CDCl_3_) δ 6.88 (dd, *J* = 5.0, 3.1 Hz, 2H, *2H_Ar_*), 6.75 (dd, *J* = 8.3, 1.9 Hz, 1H, *H_Ar_*), 5.70 (d, *J* = 32.2 Hz, 1H, N*H*), 4.97 (s, 1H, *H_4_*), 4.69 (d, *J* = 2.3 Hz, 2H, *2H_1’_*), 3.82 (s, 3H, OC*H_3_*), 3.66 (s, 6H, 2CO_2_C*H_3_*), 2.48 (t, *J* = 2.3 Hz, 1H, *H_3’_*), 2.33 (s, 6H, 2C*H_3_*). ^13^C-NMR (CDCl_3_) δ 168.19, 149.09, 145.43, 144.19, 141.75, 119.52, 114.06, 112.02, 104.00, 79.10, 75.63, 56.87, 55.95, 51.15, 38.94, 19.75. HRMS ESI-TOF [M]^+^ *m/z* Calcd. for C_21_H_23_NO_6_: 385,1513. Found: 385,1525.

**Figure 5:**
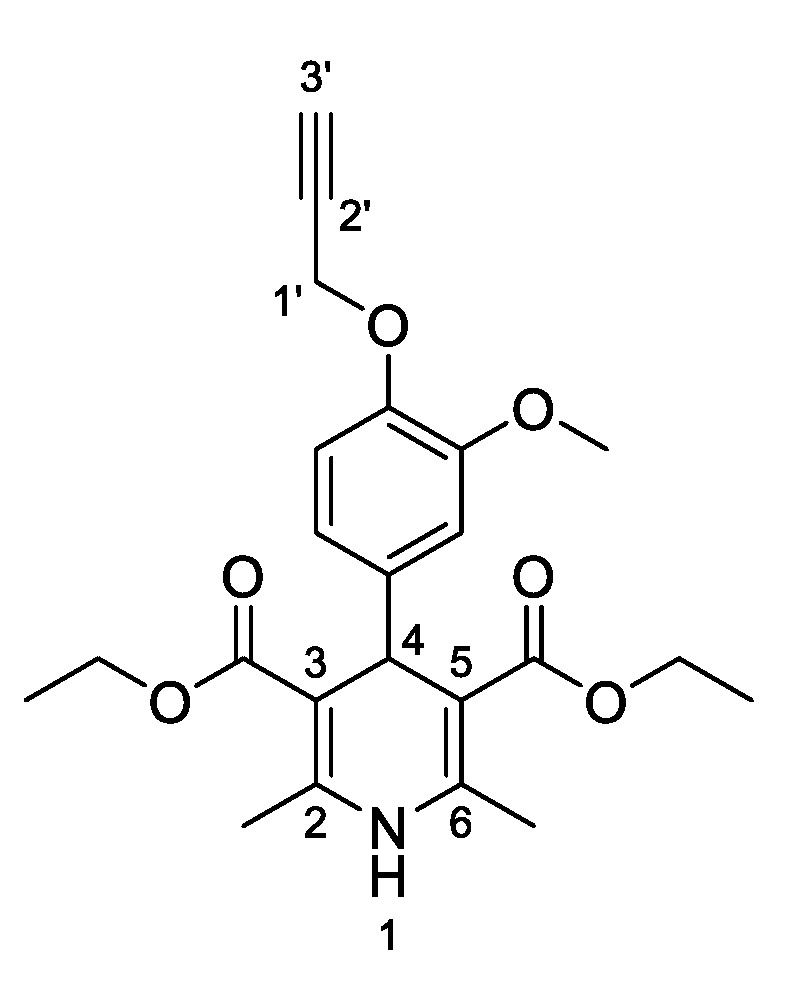
Chemical stucture of **3d**.

*3,5-Diethyl-4-[3-methoxy-4-(prop-2-yn-1-yloxy)phenyl]-2,6-dimethyl-1,4-dihydropyridine-3,5-dicarboxylate* (**3d**, Figure 5). Following the general procedure starting from 3-methoxy-4-(prop-2-yn-1-yloxy)benzaldehyde (**1b**, 1 equiv, 1.84 mmol, 350.00 mg), ethyl acetoacetate (3.5 equiv, 6.44 mmol, 0.822 mL) and ammonium carbonate (2.5 equiv, 4.60 mmol, 442.01 mg) at 75 °C over 15 h, compound **3d** (581.40 mg, 76%) precipitated as a yellow powder: ^1^H-NMR (CDCl_3_) δ 6.89 (dd, *J* = 6.3, 5.3 Hz, 2H, *2H_Ar_*), 6.78 (dd, *J* = 8.3, 2.0 Hz, 1H, *H_Ar_*), 5.61 (s, 1H, N*H*), 4.95 (s, 1H, *H_4_*), 4.69 (d, J = 2.4 Hz, 1H, *2H_1’_*), 4.18 – 4.04 (m, 4H, 2CO_2_C*H**_2_***CH_3_), 3.83 (s, 3H, OC*H_3_*), 2.47 (t, *J* = 2.4 Hz, 1H, *H_3’_*), 2.33 (s, 6H, 2C*H_3_*), 1.23 (t, *J* = 7.1 Hz, 6H, *2*CO_2_CH_2_C*H**_3_***). ^13^C-NMR (CDCl_3_) δ 167.79, 148.96, 145.35, 143.79, 142.23, 119.91, 114.07, 112.37, 104.34, 79.11, 75.59, 59.87, 56.92, 55.92, 39.28, 19.78, 14.48. HRMS ESI-TOF [M]^+^ *m/z* Calcd. for C_23_H_27_NO_6_: 413,1829. Found: 413,1838.

**Figure 6:**
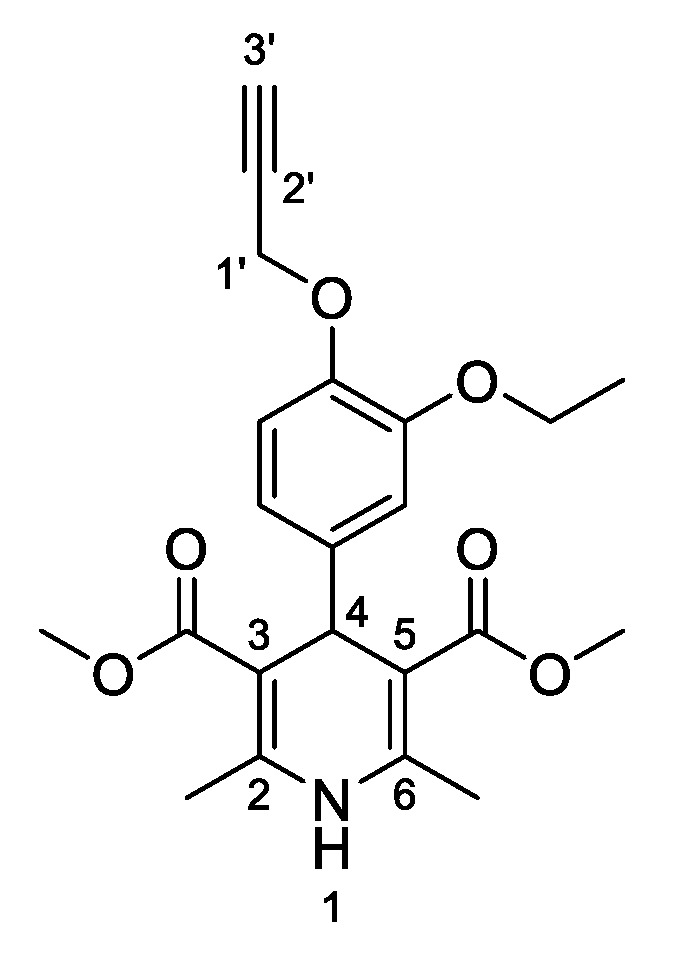
Chemical stucture of **3e**.

*3,5-Dimethyl-4-[3-ethoxy-4-(prop-2-yn-1-yloxy)phenyl]-2,6-dimethyl-1,4-dihydropyridine-3,5-dicarboxylate* (**3e**, Figure 6). Following the general procedure starting from 3-ethoxy-4-(prop-2-yn-1-yloxy)benzaldehyde (**1c**, 1 equiv, 1.22 mmol, 191.30 mg), methyl acetoacetate (3.5 equiv, 4.30 mmol, 0.462 mL) and ammonium carbonate (2.5 equiv, 3.06 mmol, 294.04 mg) at 75° C over 15 h, a precipitated was isolated that was filtered and washed in diethyl ether:pentane (1:2 v/v) to afford compound **3a** (192.70 mg, 40%) as a white powder: ^1^H-NMR (CDCl_3_) δ 6.88 (dd, *J* = 7.7, 5.1 Hz, 2H, 2*H_Ar_*), 6.75 (dd, *J* = 8.3, 1.9 Hz, 1H, *H_Ar_*), 5.61 (s, 1H, N*H*), 4.95 (s, 1H, *H_4_*), 4.69 (d, J = 2.2 Hz, 1H, 2*H_1’_*), 4.06 (q, *J* = 7.0 Hz, 2H, OC*H**_2_***CH_3_), 3.65 (s, 6H, 2CO_2_C*H_3_*), 2.47 (t, *J* = 2.3 Hz, 1H, *H_3’_*), 2.33 (s, 6H, 2C*H_3_*), 1.42 (t, *J* = 7.0 Hz, 3H, OCH_2_C*H**_3_***). ^13^C-NMR (CDCl_3_) δ 168.21, 148.45, 145.76, 144.17, 141.89, 119.62, 114.91, 113.68, 104.02, 79.32, 75.50, 64.44, 57.10, 51.15, 38.90, 19.74, 14.96. HRMS ESI-TOF [M]^+^ *m/z* Calcd. for C_22_H_25_NO_6_: 399,1673. Found: 399,1682.

**Figure 7:**
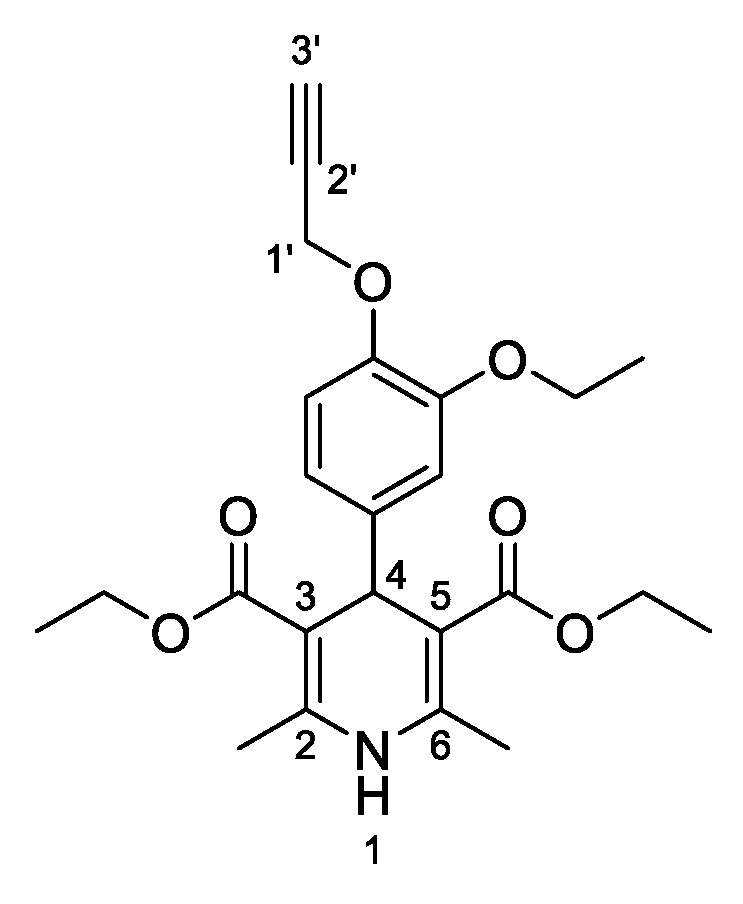
Chemical stucture of **3f**.

*3,5-Diethyl-4-[3-ethoxy-4-(prop-2-yn-1-yloxy)phenyl]-2,6-dimethyl-1,4-dihydropyridine-3,5-dicarboxylate* (**3f**, Figure 7). Following the general procedure starting from 3-ethoxy-4-(prop-2-yn-1-yloxy)benzaldehyde (**1c**, 1 equiv, 1.01 mmol, 205.00 mg), ethyl acetoacetate (3.5 equiv, 3.54 mmol, 0.452 mL) and ammonium carbonate (2.5 equiv, 2.53 mmol, 242.63 mg) at 75 °C over 15 h, the solvent was removed under pressure conditions and the residue was triturated with diethyl ether. The resulting solid was stirred in diethyl ether overnight and filtered to afford compound **3f** (272.90 mg, 52%) as abrown powder: ^1^H-NMR (CDCl_3_) δ 6.89 (dd, *J* = 5.0, 3.2 Hz, 2H, 2*H_Ar_*), 6.77 (dd, *J* = 8.3, 1.8 Hz, 1H, *H_Ar_*), 5.54 (s, 1H, N*H*), 4.94 (s, 1H, *H_4_*), 4.69 (d, *J* = 2.3 Hz, 2H, 2*H_1’_*), 4.16 – 3.98 (m, 6H, OC*H**_2_***CH_3_ and 2CO_2_C*H**_2_***CH_3_), 2.46 (t, *J* = 2.3 Hz, 1H, *H_3’_*), 2.33 (s, 6H, 2C*H_3_*), 1.42 (t, *J* = 7.0 Hz, 3H, OCH_2_C*H**_3_***), 1.23 (t, *J* = 7.1 Hz, 6H, 2CO_2_CH_2_**C*H_3_***). ^13^C-NMR (CDCl_3_) δ 167.80, 148.45, 145.71, 143.72, 142.29, 120.02, 114.93, 114.01, 104.38, 79.37, 75.45, 64.46, 59.88, 57.18, 39.22, 19.81, 15.04, 14.48. HRMS ESI-TOF [M]^+^ *m/z* Calcd. for C_24_H_29_NO_6_: 427,1977. Found: 427,1995. 

**Figure 8:**
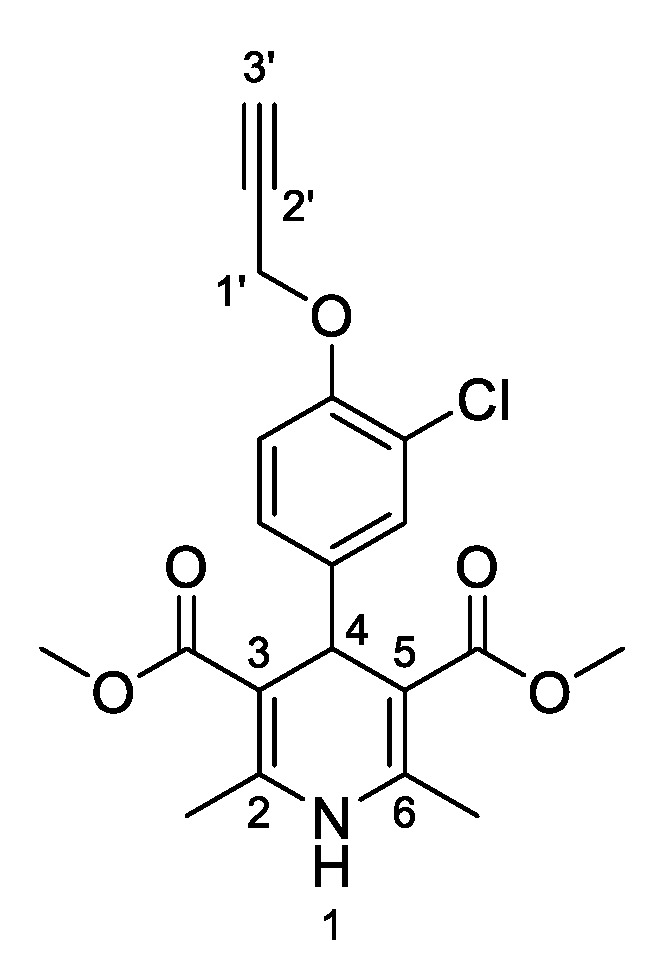
Chemical stucture of **3g**.

*3,5-Dimethyl-4-[3-chloro-4-(prop-2-yn-1-yloxy)phenyl]-2,6-dimethyl-1,4-dihydropyridine-3,5-dicarboxylate* (**3g**, Figure 8). Following the general procedure starting from 3-chloro-4-(prop-2-yn-1-yloxy)benzaldehyde (**1d**, 1 equiv, 1.54 mmol, 300.00 mg), methyl acetoacetate (3.5 equiv, 5.39 mmol, 0.578 mL) and ammonium carbonate (2.5 equiv, 3.85 mmol, 369.95 mg) at 75 °C over 15 h, the observed precipitate was filtered and washed in diethyl ether to afford compound **3g** (405.71 mg, 68%) as a white solid: ^1^H-NMR (400 MHz, CDCl_3_) δ 7.23 (d, *J* = 2.1 Hz, 1H, *H_Ar_*), 7.13 (dd, *J* = 8.5, 2.1 Hz, 1H, *H_Ar_*), 6.94 (d, *J* = 8.5 Hz, 1H, *H_Ar_*), 5.64 (s, 1H, N*H*), 4.94 (s, 1H, *H_4_*), 4.71 (d, *J* = 2.3 Hz, 2H, 2*H_1’_*), 3.66 (s, 6H, 2CO_2_C*H_3_*), 2.52 (t, *J* = 2.3 Hz, 1H, *H_3’_*), 2.34 (s, 6H, 2C*H_3_*). ^13^C-NMR (101 MHz, CDCl_3_) δ 167.96, 151.68, 144.38, 142.15, 129.74, 126.98, 122.80, 114.02, 103.76, 78.46, 76.09, 57.02, 51.22, 38.67, 19.83. HRMS ESI-TOF [M]^+^ *m/z* Calcd. for C_20_H_20_ClNO_5_: 389,1024. Found: 389,1030.

**Figure 9:**
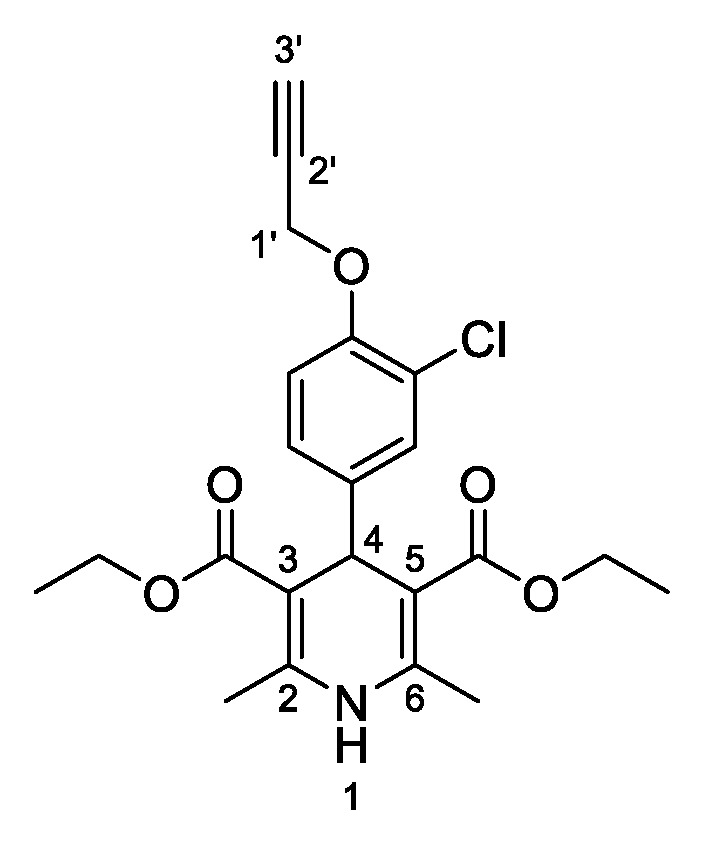
Chemical stucture of **3h**.

*3,5-Diethyl-4-[3-chloro-4-(prop-2-yn-1-yloxy)phenyl]-2,6-dimethyl-1,4-dihydropyridine-3,5-dicarboxylate* (**3h**, Figure 9**)**. Following the general procedure starting from 3-chloro-4-(prop-2-yn-1-yloxy)benzaldehyde (**1d**, 1 equiv, 1.54 mmol, 300.00 mg), ethyl acetoacetate (3.5 equiv, 5.39 mmol, 0.688 mL) and ammonium carbonate (2.5 equiv, 3.85 mmol, 369.95 mg) at 75 °C over 15 h, the precipitate was filtered and washed with diethyl ether to afford compound **3h** (416.9 mg, 68%) as a white solid: ^1^H-NMR (CDCl_3_) δ 7.29 (s, 1H, *H_Ar_*), 7.16 (dd, *J* = 8.5, 2.2 Hz, 1H, *H_Ar_*), 6.95 (d, *J* = 8.5 Hz, 1H, *H_Ar_*), 5.61 (s, 1H, N*H*), 4.94 (s, 1H, *H_4_*), 4.74 (d, *J* = 2.4 Hz, 2H, 2*H_1’_*), 4.21 – 4.03 (m, 4H, 2CO_2_C*H_2_*CH_3_), 2.54 (t, *J* = 2.4 Hz, 1H, *H_3’_*), 2.36 (s, 6H, 2C*H_3_*), 1.25 (t, *J* = 7.1 Hz, 6H, 2CO_2_CH_2_C*H**_3_***). ^13^C-NMR (CDCl_3_) δ 296.64, 280.65, 273.12, 271.69, 259.32, 256.41, 251.67, 243.07, 233.13, 207.55, 205.15, 189.07, 186.15, 168.17, 148.91, 143.51. Anal. Calcd. for C_22_H_24_ClNO_5_: C, 63.23; H, 5.79; N, 3.35. Found: C, 62.93; H, 5.88; N, 3.39.

**Figure 10:**
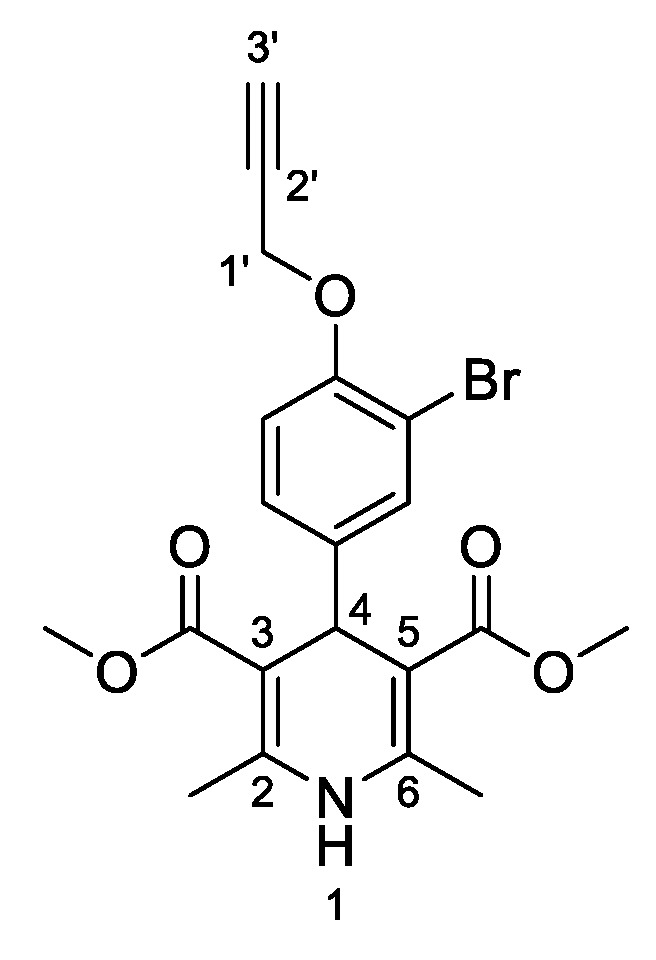
Chemical stucture of **3i**.

*3,5-Dimethyl-4-[3-bromo-4-(prop-2-yn-1-yloxy)phenyl]-2,6-dimethyl-1,4-dihydropyridine-3,5-dicarboxylate* (**3i**, Figure 10). Following the general procedure starting from 3-bromo-4-(prop-2-yn-1-yloxy)benzaldehyde (**1e**, 1 equiv, 1.05 mmol, 250.00 mg), methyl acetoacetate (3.5 equiv, 3.66 mmol, 0.395 mL) and ammonium carbonate (2.5 equiv, 2.63 mmol, 252.24 mg) at 75 °C over 15 h, the precipitate was filtered and washed with diethyl diethyl ether:pentane (1:1 v/v) to afford compound **3i** (266.20 mg, 58%) as a white solid: ^1^H-NMR (CDCl_3_) δ 7.40 (d, *J* = 2.2 Hz, 1H, *H_Ar_*), 7.17 (dd, *J* = 8.5, 2.2 Hz, 1H, *H_Ar_*), 6.92 (d, *J* = 8.5 Hz, 1H, *H_Ar_*), 5.63 (s, 1H, N*H*), 4.94 (s, 1H, *H_4_*), 4.71 (d, *J* = 2.4 Hz, 2H, 2*H_1’_*), 3.66 (s, *J* = 6.2 Hz, 6H, 2CO_2_C*H_3_*), 2.52 (t, *J* = 2.4 Hz, 1H, *H_3’_*), 2.34 (s, 6H, 2C*H_3_*). ^13^C-NMR (CDCl_3_) δ 167.95, 152.59, 144.38, 142.54, 132.78, 127.78, 113.81, 112.07, 103.77, 78.45, 76.10, 57.07, 51.22, 38.62, 19.84. HRMS ESI-TOF [M]^+^ *m/z* Calcd. for C_20_H_20_BrNO_5_: 433,0516. Found: 433,0525.

**Figure 11:**
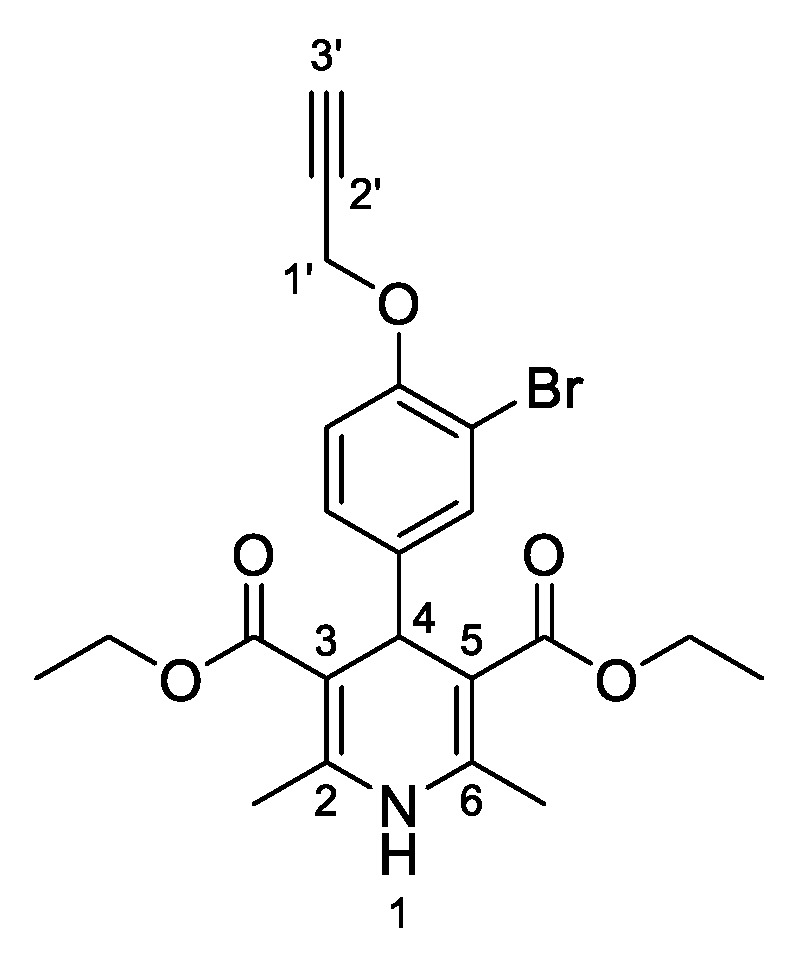
Chemical stucture of **3j**.

*3,5-Diethyl-4-[3-bromo-4-(prop-2-yn-1-yloxy)phenyl]-2,6-dimethyl-1,4-dihydropyridine-3,5-dicarboxylate* (**3j**, Figure 11). Following the general procedure starting from 3-bromo-4-(prop-2-yn-1-yloxy)benzaldehyde (**1e**, 1 equiv, 1.05 mmol, 205.00 mg), ethyl acetoacetate (3.5 equiv, 3.66 mmol, 0.467 mL) and ammonium carbonate (2.5 equiv, 2.63 mmol, 252.24 mg) at 70 °C over 15 h, the solvent was removed under pressure conditions and the residue was triturated with diethyl ether. The resulting solid was stirred in diethyl ether:pentane (1:1 v/v) overnight and filtered to afford compound **3j** (202.71 mg, 42%) as white powder: ^1^H-NMR (CDCl_3_) δ 7.43 (d, *J* = 2.1 Hz, 1H, *H_Ar_*), 7.18 (dd, *J* = 8.5, 2.1 Hz, 1H, *H_Ar_*), 6.91 (d, *J* = 8.5 Hz, 1H, *H_Ar_*), 5.56 (s, 1H, N*H*), 4.91 (s, 1H, *H_4_*), 4.71 (d, *J* = 2.4 Hz, 2H, 2*H_1’_*), 4.22 – 4.01 (m, 4H, 2CO_2_C*H**_2_***CH_3_), 2.51 (t, *J* = 2.4 Hz, 1H, *H_3’_*), 2.34 (s, 6H, 2C*H_3_*), 1.23 (t, *J* = 7.1 Hz, 6H, 2CO_2_CH_2_C*H**_3_***). ^13^C-NMR (CDCl_3_) δ 167.51, 152.46, 143.99, 142.98, 133.29, 128.09, 113.76, 111.78, 104.06, 78.44, 76.06, 59.97, 57.10, 39.03, 19.83, 14.42. HRMS ESI-TOF [M]^+^ *m/z* Calcd. for C_22_H_24_BrNO_5_: 461,0829. Found: 461,0838.

**Figure 12:**
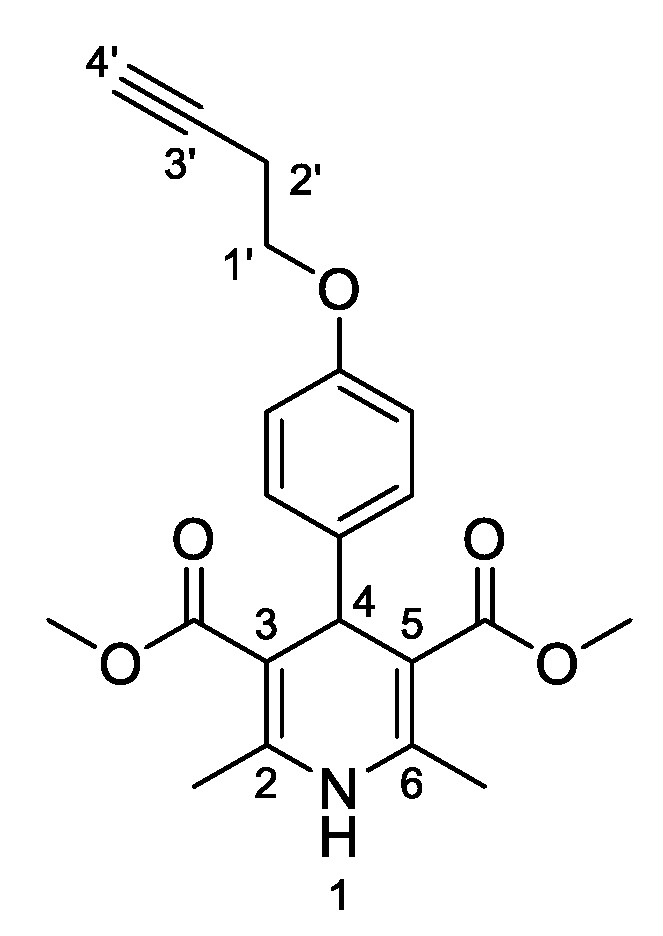
Chemical stucture of **3k**.

*3,5-Dimethyl-4-[4-(but-3-yn-1-yloxy)phenyl]-2,6-dimethyl-1,4-dihydropyridine-3,5-dicarboxylate* (**3k**, Figure 12). Following the general procedure starting from 4-(but-3-yn-1-yloxy)benzaldehyde (**2a**) (1 equiv, 1.43 mmol, 250.00 mg), methyl acetoacetate (3.5 equiv, 5.02 mmol, 0.640 mL) and ammonium carbonate (2.5 equiv, 3.58 mmol, 343.52 mg) at 75 °C over 15 h, the solvent was removed under pressure conditions and the resulting residue was purified by flash column chromatography using hexane/EtOAc (7/·3) + 1% Et_3_N, to afford compound **3k** (144.2 mg, 27%) as white crystals: ^1^H-NMR (CDCl_3_) δ 7.21–7.10 (m, 2H, 2*H_Ar_*), 6.79–6.72 (m, 2H, 2*H_Ar_*), 5.61 (s, 1H, N*H*), 4.94 (s, 1H, *H_4_*), 4.04 (t, *J* = 7.0 Hz, 2H, 2*H_1’_*), 3.64 (s, 6H, 2CO_2_C*H_3_*), 2.64 (td, *J* = 7.0, 2.7 Hz, 2H, 2*H_2’_*), 2.33 (s, 6H, 2C*H_3_*), 2.01 (t, *J* = 2.7 Hz, 1H, *H_4’_*). ^13^C-NMR (CDCl_3_) δ 168.20, 156.93, 144.03, 140.51, 129.01, 128.82, 114.28, 114.19, 104.27, 80.72, 69.90, 66.01, 51.13, 38.60, 19.77, 19.70. HRMS ESI-TOF [M]^+^ *m/z* Calcd. for C_21_H_23_NO_5_: 397,1879. Found: 397,1889.

**Figure 13:**
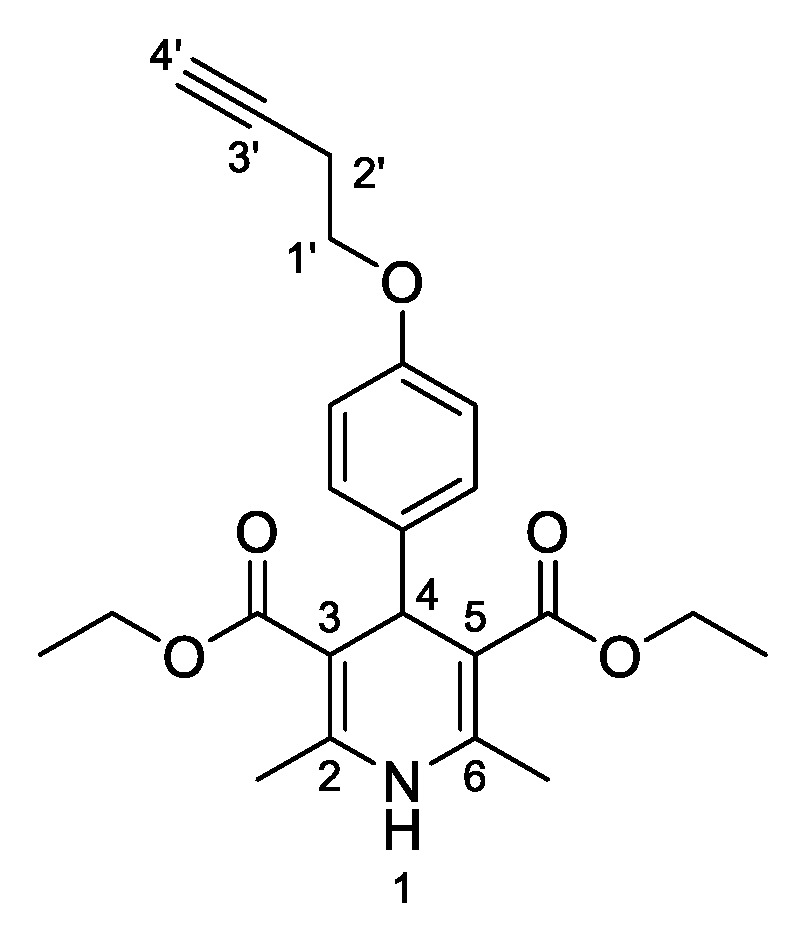
Chemical stucture of **3l**.

*3,5-Diethyl-4-[4-(but-3-yn-1-yloxy)phenyl]-2,6-dimethyl-1,4-dihydropyridine-3,5-dicarboxylate* (**3l**, Figure 13). Following the general procedure starting from 4-(but-3-yn-1-yloxy)benzaldehyde (**2a**, 1 equiv, 1.43 mmol, 250.00 mg), ethyl acetoacetate (3.5 equiv, 5.02 mmol, 0.540 mL) and ammonium carbonate (2.5 equiv, 3.58 mmol, 343.52 mg) at 75 °C over 15 h, the solvent was removed under pressure conditions and the residue was triturated with diethyl ether. The resulting residue was recrystallized in DCM/diethyl ether (1:1 v/v) to afford compound **3l** (70.10 mg, 12%) as a white solid: ^1^H-NMR (CDCl_3_) δ 7.23–7.11 (m, 2H, 2*H_Ar_*), 6.78–6.72 (m, 2H, 2*H_Ar_*), 5.57 (s, 1H, N*H*), 4.92 (s, 1H, *H_4_*), 4.15–3.99 (m, 6H, 2CO_2_C*H**_2_***CH_3_ and 2*H_1’_*), 2.64 (td, *J* = 7.0, 2.7 Hz, 2H, 2*H_2’_*), 2.32 (s, 6H, 2C*H_3_*), 2.01 (t, *J* = 2.7 Hz, 1H, *H_4’_*), 1.22 (t, *J* = 7.1 Hz, 6H, 2CO_2_CH_2_C*H**_3_***). ^13^C-NMR (CDCl_3_) δ 167.79, 156.86, 143.65, 140.92, 129.18, 114.11, 104.54, 80.73, 69.90, 66.03, 59.84, 38.93, 19.77, 19.70, 14.42. HRMS ESI-TOF [M]^+^ *m/z* Calcd. for C_23_H_27_NO_5_: 369,1570. Found: 369,1576.

**Figure 14:**
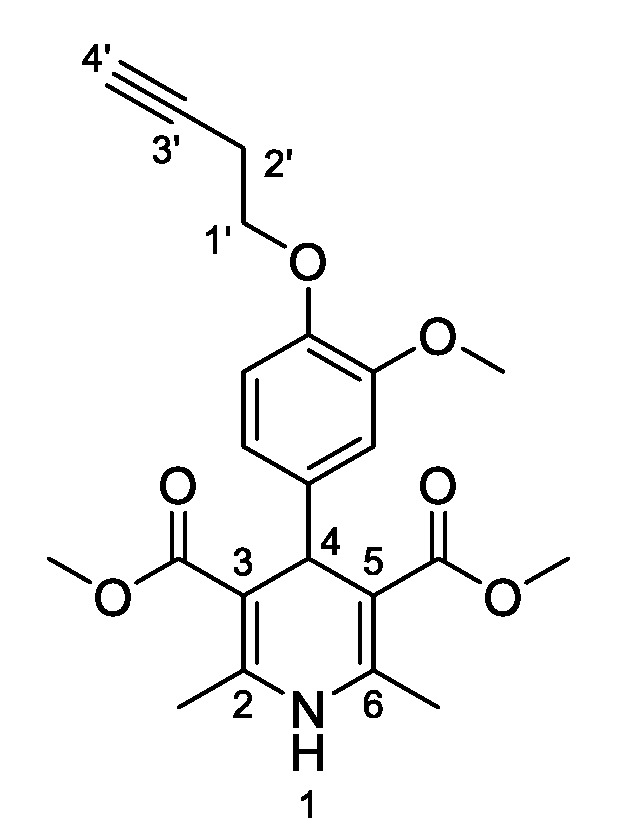
Chemical stucture of **3m**.

*3,5-Dimethyl-4-[4-(but-3-yn-1-yloxy)-3-methoxyphenyl]-2,6-dimethyl-1,4-dihydropyridine-3,5-dicarboxylate* (**3m**, Figure 14). Following the general procedure starting from 4-(but-3-yn-1-yloxy)-3-methoxybenzaldehyde (**2b**, 1 equiv, 1.47 mmol, 300.00 mg), methyl acetoacetate (3.5 equiv, 5.14 mmol, 0.594 mL) and ammonium carbonate (2.5 equiv, 3.68 mmol, 353.13 mg) at 75 °C over 15 h, the precipitate was filtered and washed in diethyl ether:pentane (1:1 v/v) to afford compound **3m** (420.5 mg, 72%) as a white solid: ^1^H-NMR (CDCl_3_) δ 6.87 (s, 1H, *H_Ar_*), 6.74 (d, *J* = 0.8 Hz, 2H, 2*H_Ar_*), 5.63 (s, 1H, N*H*), 4.95 (s, 1H, *H_4_*), 4.10 (t, *J* = 7.4 Hz, 2H, 2*H_1’_*), 3.82 (s, 3H, OC*H_3_*), 3.66 (s, 6H, 2CO_2_C*H_3_*), 2.68 (td, *J* = 7.4, 2.7 Hz, 2H, 2*H_2’_*), 2.34 (s, 6H, 2C*H_3_*), 2.01 (t, *J* = 2.7 Hz, 1H, *H_4’_*). ^13^C-NMR (CDCl_3_) δ 168.19, 149.13, 146.29, 144.09, 141.35, 119.75, 113.79, 112.39, 104.09, 80.54, 70.03, 67.23, 56.14, 51.14, 38.94, 19.78, 19.61. Anal. Calcd. for C_22_H_25_NO_5_: C, 66.15; H, 6.31; N, 3.51. Found: C, 66.23; H, 6.36; N, 3.59.

**Figure 15:**
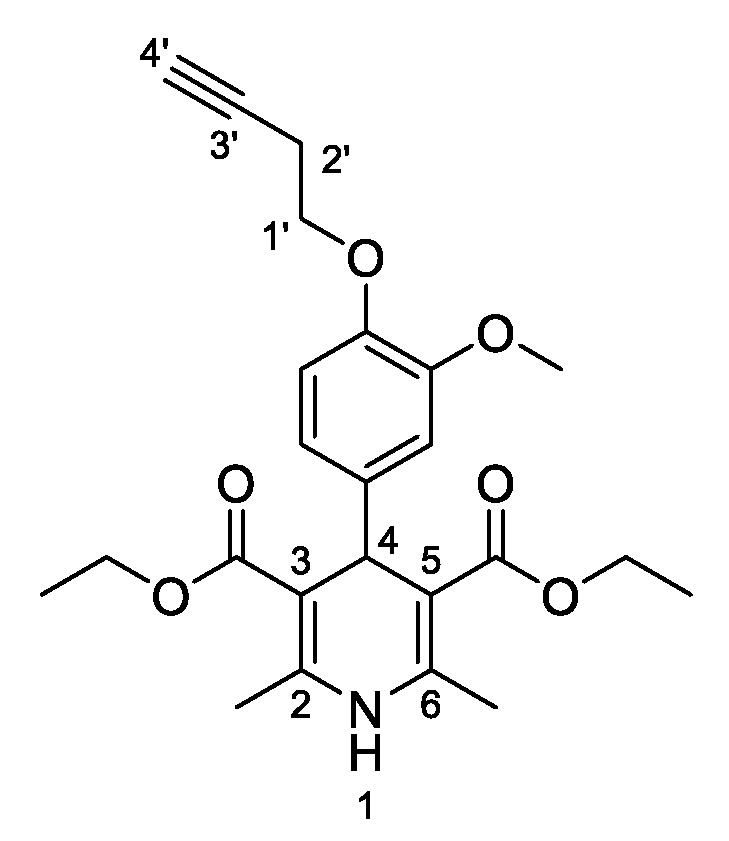
Chemical stucture of **3n**.

*3,5-Diethyl-4-[4-(but-3-yn-1-yloxy)-3-methoxyphenyl]-2,6-dimethyl-1,4-dihydropyridine-3,5-dicarboxylate* (**3n**, Figure 15). Following the general procedure starting from 4-(but-3-yn-1-yloxy)-3-methoxybenzaldehyde (**2b**, 1 equiv, 1.47 mmol, 300.00 mg), ethyl acetoacetate (3.5 equiv, 5.14 mmol, 0.656 mL) and ammonium carbonate (2.5 equiv, 3.68 mmol, 353.13 mg) at 75 °C over 15 h, the solvent was removed under pressure conditions and the residue was triturated with diethyl ether. The resulting solid was stirred in diethyl ether:pentane (1:1 v/v) overnight and filtered to afford compound **3n** (520.92 mg, 83%) as a light yellowish solid: ^1^H-NMR (CDCl_3_) δ 6.88 (d, *J* = 1.7 Hz, 1H, *H_Ar_*), 6.79–6.70 (m, 2H, 2*H_Ar_*), 5.55 (s, 1H, N*H*), 4.94 (s, 1H, *H_4_*), 4.10 (pd, *J* = 8.2, 3.7 Hz, 6H, 2CO_2_C*H**_2_***CH_3_ and 2*H_1’_*), 3.82 (s, 3H, OC*H_3_*), 2.68 (td, *J* = 7.5, 2.7 Hz, 2H, 2*H_2’_*), 2.33 (s, 6H, 2C*H_3_*), 2.01 (t, *J* = 2.7 Hz, 1H, *H_4’_*), 1.23 (t, *J* = 7.1 Hz, 6H, 2CO_2_CH_2_C*H**_3_***). ^13^C-NMR (CDCl_3_) δ 167.78, 148.99, 146.21, 143.70, 141.81, 120.14, 113.78, 112.73, 104.42, 80.56, 70.01, 67.27, 59.88, 56.12, 39.26, 19.81, 19.62, 14.49. HRMS ESI-TOF [M]^+^ *m/z* Calcd. for C_24_H_29_NO_6_: 427,1985. Found: 427,1995.

**Figure 16:**
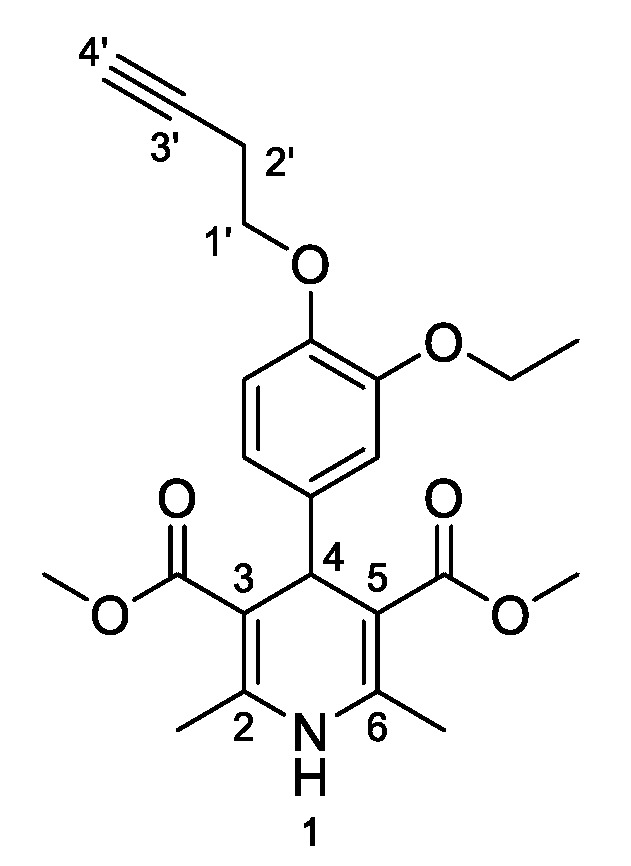
Chemical stucture of **3o**.

*3,5-Dimethyl-4-[4-(but-3-yn-1-yloxy)-3-ethoxyphenyl]-2,6-dimethyl-1,4-dihydropyridine-3,5-dicarboxylate* (**3o**, Figure 16). Following the general procedure starting from 4-(but-3-yn-1-yloxy)-3-ethoxybenzaldehyde (**2c**, 1 equiv, 1.60 mmol, 350.00 mg), methyl acetoacetate (3.5 equiv, 5.61 mmol, 0.679 mL) and ammonium carbonate (2.5 equiv, 4.00 mmol, 384.36 mg) at 75 °C over 15 h, the precipitate was filtered and washed in diethyl ether:pentane (1:1 v/v) to afford compound **3o** (288.40 mg, 44%) as a white solid: ^1^H-NMR (CDCl_3_) δ 6.89 (s, 1H, *H_Ar_*), 6.77 (s, 2H, 2*H_Ar_*), 5.67 (s, 1H, N*H*), 4.95 (s, 1H, *H_4_*), 4.09 (dt, *J* = 2.3, 7.1 Hz, 4H, OC***H_2_***CH_3_ and 2*H_1’_*), 3.67 (s, 6H, 2CO_2_C*H_3_*), 2.69 (td, *J* = 7.4, 2.6 Hz, 2H, 2*H_2’_*), 2.35 (s, 6H, 2C*H_3_*), 2.02 (t, *J* = 2.6 Hz, 1H, *H_4’_*), 1.42 (t, *J* = 7.0 Hz, 3H, OCH_2_C*H**_3_***). ^13^C-NMR (CDCl_3_) δ 168.20, 148.54, 146.73, 144.08, 141.58, 120.21, 120.01, 114.84, 114.43, 104.10, 80.72, 69.88, 67.61, 64.76, 51.13, 38.91, 19.75, 19.68, 15.03. HRMS ESI-TOF [M]^+^ *m/z* Calcd. for C_23_H_27_NO_6_: 413,1831. Found: 413,1838.

**Figure 17:**
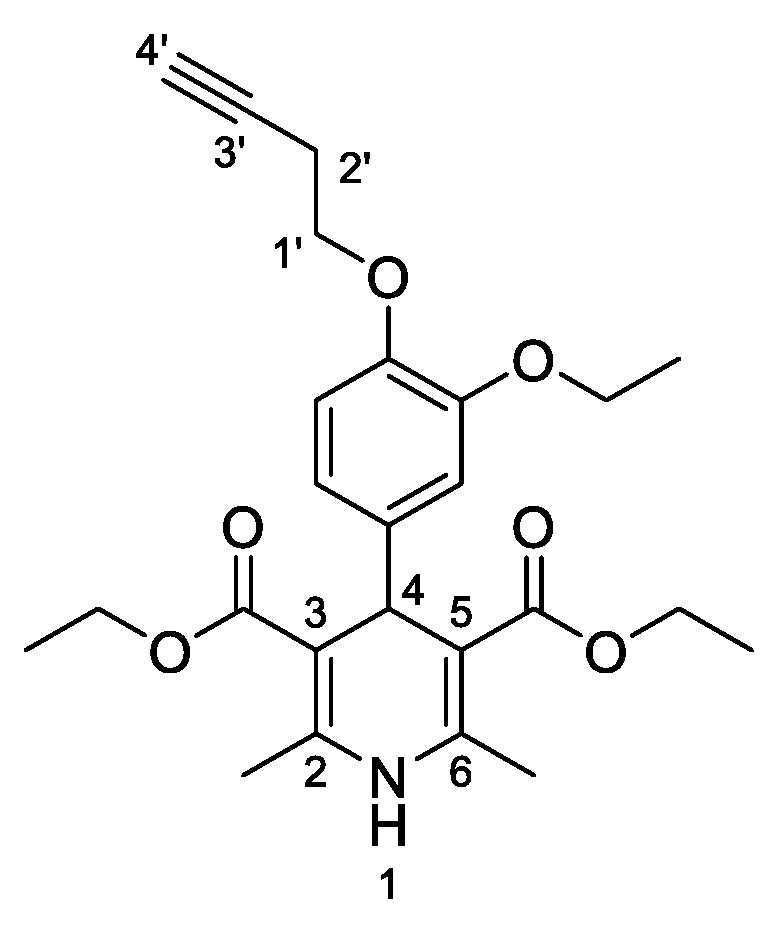
Chemical stucture of **3p**.

*3,5-Diethyl-4-[4-(but-3-yn-1-yloxy)-3-ethoxyphenyl]-2,6-dimethyl-1,4-dihydropyridine-3,5-dicarboxylate* (**3p**, Figure 17). Following the general procedure starting from 4-(but-3-yn-1-yloxy)-3-ethoxybenzaldehyde (**2c**, 1 equiv, 1.60 mmol, 350.00 mg), ethyl acetoacetate (3.5 equiv, 5.61 mmol, 0.716 mL) and ammonium carbonate (2.5 equiv, 4.00 mmol, 384.36 mg) at 75 °C over 15 h, the precipitate was filtered and washed in diethyl ether:pentane (1:1 v/v) to afford compound **3p** (340.31 mg, 48%) as a white solid: H-NMR (CDCl_3_) δ 6.88 (d, *J* = 1.3 Hz, 1H, *H_Ar_*), 6.81–6.70 (m, 2H, 2*H_Ar_*), 5.52 (s, 1H, N*H*), 4.93 (s, 1H, *H_4_*), 4.17 – 3.99 (m, 8H, OC*H**_2_***CH_3_ and 2*H_1’_* and 2CO_2_C*H**_2_***CH_3_), 2.67 (td, *J* = 7.4, 2.7 Hz, 2H, 2*H_2’_*), 2.33 (s, 6H, 2C*H_3_*), 2.00 (t, *J* = 2.7 Hz, 1H, *H_4’_*), 1.40 (t, *J* = 7.0 Hz, 3H, OCH_2_C*H**_3_***), 1.23 (t, *J* = 7.1 Hz, 6H, 2CO_2_CH_2_C*H**_3_***). ^13^C-NMR (CDCl_3_) δ 167.79, 148.52, 146.68, 143.64, 141.97, 120.41, 114.81, 114.76, 104.44, 80.71, 69.86, 67.66, 64.80, 59.86, 39.20, 19.81, 19.70, 15.10, 14.48. Anal. Calcd. for C_25_H_31_NO_6_: C, 68.01; H, 7.08; N, 3.17. Found: C, 67.87; H, 7.15; N, 3.25. HRMS ESI-TOF [M]^+^ *m/z* Calcd. for C_25_H_31_NO_6_: 441,2137. Found: 441,2151.

**Figure 18:**
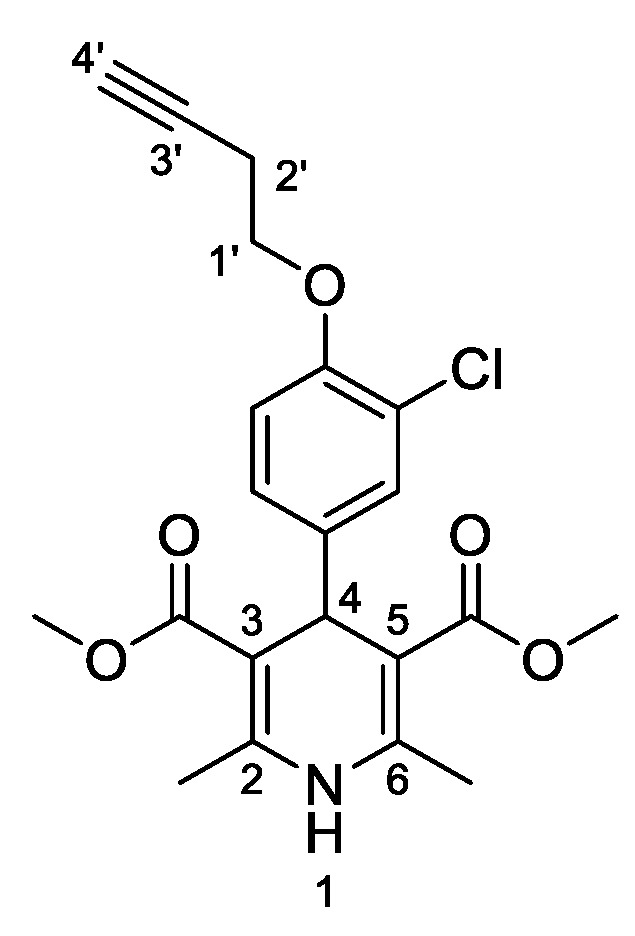
Chemical stucture of **3q**.

*3,5-Dimethyl-4-[4-(but-3-yn-1-yloxy)-3-chlorophenyl]-2,6-dimethyl-1,4-dihydropyridine-3,5-dicarboxylate* (**3q**, Figure 18). Following the general procedure starting from 4-(but-3-yn-1-yloxy)-3-chlorobenzaldehyde (**2d**, 1 equiv, 1.44 mmol, 300.00 mg), methyl acetoacetate (3.5 equiv, 5.03 mmol, 0.543 mL) and ammonium carbonate (2.5 equiv, 3.60 mmol, 345.92 mg) at 78 °C over 15 h, the solvent was removed under pressure conditions and the resulting residue was purified by flash column chromatography using hexane/EtOAc (6/4) + 1% Et_3_N, to afford compound **3q** (76.05 mg, 13%) as a white solid: ^1^H-NMR (CDCl_3_) δ 7.22 (d, *J* = 2.2 Hz, 1H, *H_Ar_*), 7.11 (dd, *J* = 8.5, 2.2 Hz, 1H, *H_Ar_*), 6.78 (d, *J* = 8.5 Hz, 1H, *H_Ar_*), 5.64 (s, 1H, N*H*), 4.92 (s, 1H, *H_4_*), 4.10 (t, *J* = 7.2 Hz, 2H, 2*H_1’_*), 3.65 (s, *J* = 4.3 Hz, 6H, 2CO_2_C*H**_3_***), 2.70 (td, *J* = 7.2, 2.7 Hz, 2H, 2*H_2’_*), 2.34 (s, 6H, 2C*H_3_*), 2.02 (t, *J* = 2.7 Hz, 1H, *H_4’_*). ^13^C-NMR (CDCl_3_) δ 167.84, 152.29, 144.19, 141.61, 129.55, 126.95, 122.72, 113.59, 103.67, 80.11, 70.01, 67.27, 51.06, 38.55, 19.68, 19.48. HRMS ESI-TOF [M]^+^ *m/z* Calcd. for C_21_H_22_ClNO_5_: 403,1180. Found: 403,1187.

**Figure 19:**
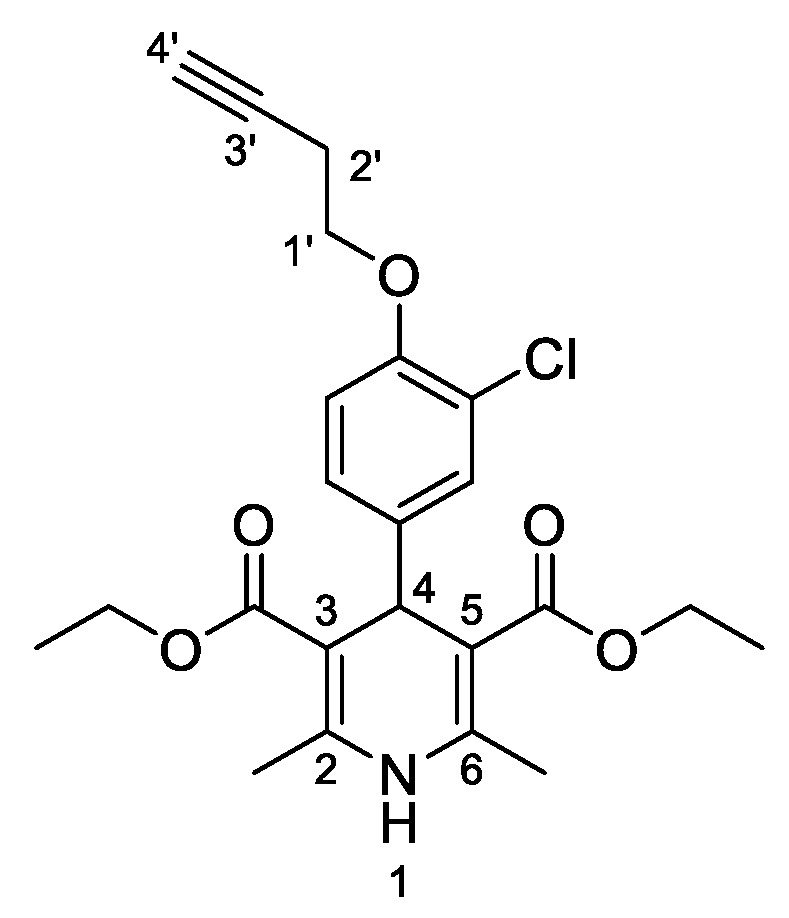
Chemical stucture of **3r**.

*3,5-Diethyl-4-[4-(but-3-yn-1-yloxy)-3-chlorophenyl]-2,6-dimethyl-1,4-dihydropyridine-3,5-dicarboxylate* (**3r**, Figure 19). Following the general procedure starting from 4-(but-3-yn-1-yloxy)-3-chlorobenzaldehyde (**2d**) (1 equiv, 1.44 mmol, 300.00 mg), ethyl acetoacetate (3.5 equiv, 5.03 mmol, 0.643 mL) and ammonium carbonate (2.5 equiv, 3.60 mmol, 345.92 mg) at 79 °C over 15 h, the solvent was removed under pressure conditions and the resulting residue was purified by flash column chromatography using hexane/EtOAc (6/4) + 1% Et_3_N, to afford compound **3r** (80.23 mg, 13%) as a white solid: ^1^H-NMR (CDCl_3_) δ 7.25 (d, *J* = 2.2 Hz, 1H, *H_Ar_*), 7.12 (dd, *J* = 8.4, 2.2 Hz, 1H, *H_Ar_*), 6.78 (d, *J* = 8.5 Hz, 1H, *H_Ar_*), 5.57 (s, 1H, N*H*), 4.90 (s, 1H, *H_4_*), 4.20–4.01 (m, 6H, 2*H_1’_* and 2CO_2_**C*H_2_***CH_3_), 2.70 (td, *J* = 7.3, 2.7 Hz, 2H, 2*H_2’_*), 2.33 (s, 6H, 2CH_3_), 2.02 (t, *J* = 2.7 Hz, 1H, *H_4’_*), 1.23 (t, *J* = 7.1 Hz, 6H, 2CO_2_CH_2_**C*H_3_***). ^13^C-NMR (CDCl_3_) δ 167.55, 152.33, 143.95, 142.16, 130.16, 127.41, 122.61, 113.64, 104.08, 80.26, 70.14, 67.44, 59.95, 39.06, 22.09, 19.81, 19.62, 14.41. HRMS ESI-TOF [M]^+^ *m/z* Calcd. for C_23_H_26_ClNO_5_: 431,1491. Found: 431,1500.

**Figure 20:**
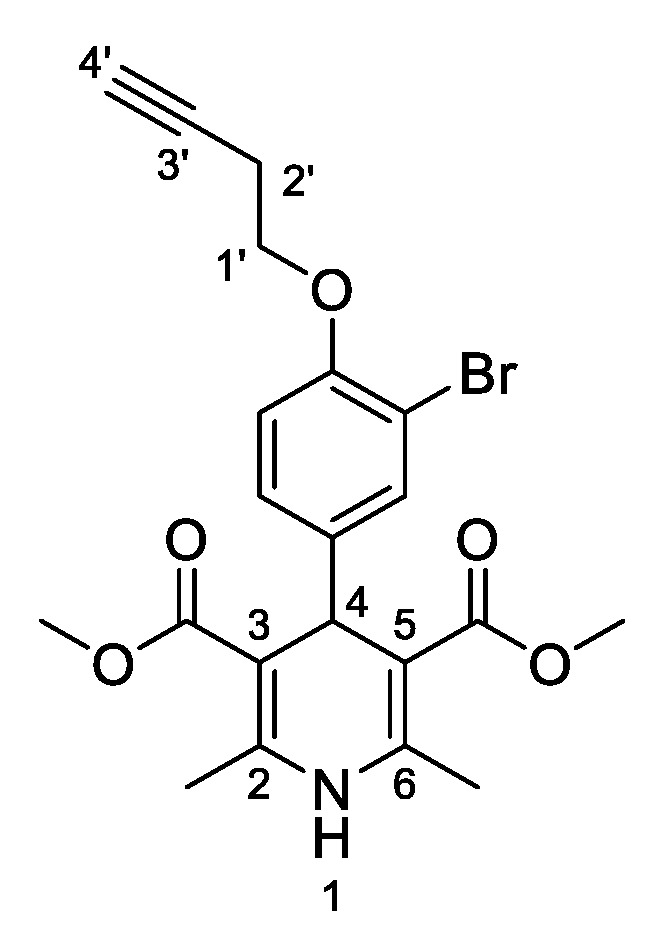
Chemical stucture of **3s**.

*3,5-Dimethyl-4-[3-bromo-4-(but-3-yn-1-yloxy)phenyl]-2,6-dimethyl-1,4-dihydropyridine-3,5-dicarboxylate* (**3s**, Figure 20). Following the general procedure starting from 3-bromo-4-(but-3-yn-1-yloxy)benzaldehyde (**2e,** 1 equiv, 0.77 mmol, 195.00 mg), methyl acetoacetate (3.5 equiv, 2.69 mmol, 0.291 mL) and ammonium carbonate (2.5 equiv, 1.93 mmol, 189.97 mg) at 75 °C over 15 h, the precipitated was filtered and washed in diethyl ether:pentane (1:3 v/v) to afford compound **3s** (142.40 mg, 23%) as a white solid: ^1^H-NMR (CDCl_3_) δ 7.38 (d, *J* = 1.8 Hz, 1H, *H_Ar_*), 7.16 (d, *J* = 8.4 Hz, 1H, *H_Ar_*), 6.76 (d, *J* = 8.4 Hz, 1H, *H_Ar_*), 5.62 (s, 1H, N*H*), 4.92 (s, 1H, *H_4_*), 4.10 (t, *J* = 7.2 Hz, 2H, 2*H_1’_*), 3.65 (s, 6H, 2CO_2_C*H**_3_***), 2.70 (td, *J* = 7.1, 2.5 Hz, 2H, 2*H_2’_*), 2.34 (s, 6H, 2CH_3_), 2.03 (d, *J* = 2.4 Hz, 1H, *H_4’_*). ^13^C-NMR (CDCl_3_) δ 167.95, 153.32, 144.30, 142.15, 132.73, 127.89, 113.49, 103.82, 80.27, 70.15, 67.46, 51.20, 38.65, 19.83, 19.62. HRMS ESI-TOF [M]^+^ *m/z* Calcd. for C_21_H_22_BrNO_5_: 447,0666. Found: 447,0681.

**Figure 21:**
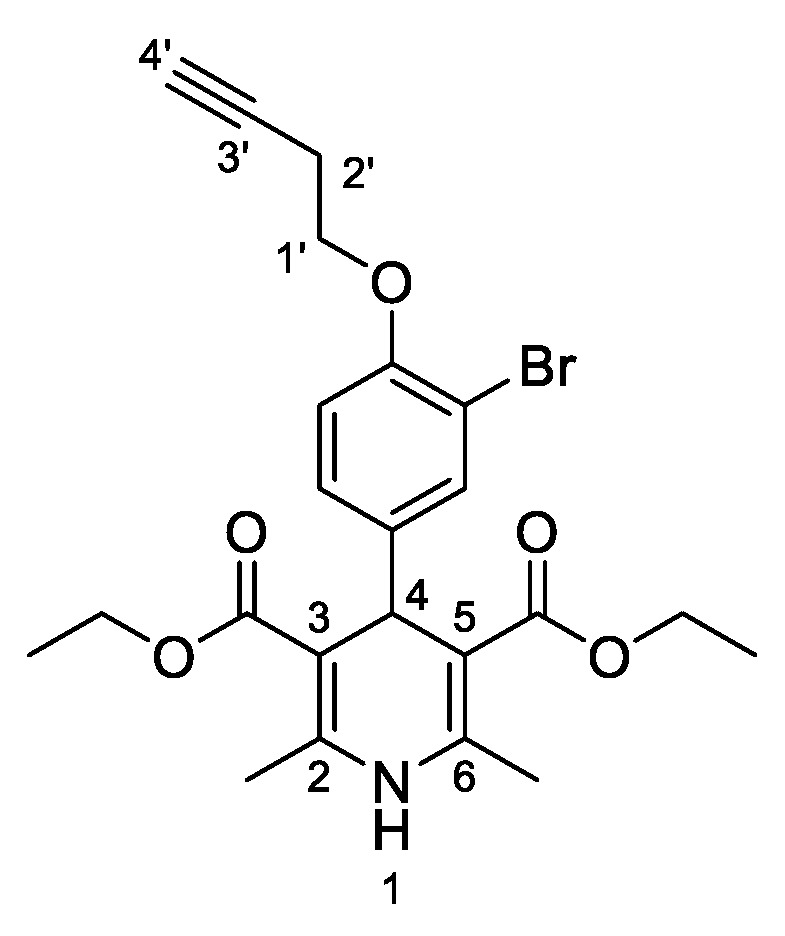
Chemical stucture of **3t**.

*3,5-Diethyl-4-[3-bromo-4-(but-3-yn-1-yloxy)phenyl]-2,6-dimethyl-1,4-dihydropyridine-3,5-dicarboxylate* (**3t**, Figure 21). Following the general procedure starting from 3-bromo-4-(but-3-yn-1-yloxy)benzaldehyde (**2e**, 1 equiv, 0.77 mmol, 195.00 mg), ethyl acetoacetate (3.5 equiv, 1.69 mmol, 0.343 mL) and ammonium carbonate (2.5 equiv, 1.93 mmol, 184.97 mg) at 85 °C over 15 h, the solvent was removed under pressure conditions and the residue was triturated with diethyl ether. The resulting solid was stirred in diethyl ether:pentane (1:1 v/v) overnight and filtered to afford compound **3t** (280.92 mg, 49%) as a white solid: ^1^H-NMR (CDCl_3_) δ 7.42 (d, *J* = 2.1 Hz, 1H, *H_Ar_*), 7.16 (dd, *J* = 8.4, 2.2 Hz, 1H, *H_Ar_*), 6.88 – 6.66 (m, 1H, *H_Ar_*), 5.55 (s, 1H, N*H*), 4.90 (s, 1H, *H_4_*), 4.31 – 4.02 (m, 6H, 2*H_1’_* and 2CO_2_C*H**_2_***CH_3_), 2.70 (td, *J* = 7.3, 2.7 Hz, 2H, 2*H_2’_*), 2.33 (s, 6H, 2CH_3_), 2.03 (t, *J* = 2.7 Hz, 1H, *H_4’_*), 1.23 (t, *J* = 7.1 Hz, 6H, 2CO_2_CH_2_C*H**_3_***). ^13^C-NMR (CDCl_3_) δ 167.56, 153.21, 144.02, 142.58, 133.20, 128.18, 113.42, 111.93, 104.03, 80.27, 70.14, 67.48, 59.95, 39.01, 19.77, 19.61, 14.41. HRMS ESI-TOF [M]^+^ *m/z* Calcd. for C_23_H_26_BrNO_5_: 475,0983. Found: 475,0994.

### 3.5. Biological Evaluation

#### 3.5.1. Calcium Channel Blockade

Human neuroblastoma cell line SH-SY5Y (CRL-2266) obtained from the American Type Culture Collection (Manassas, VA, USA) was maintained at 37°C in humidified atmosphere of 5% CO2/95% air. Mixture of Dulbecco’s Modified Eagle Medium and Nutrient Mixture F-12 (1:1) containing 10% of fetal bovine serum, was used as a culture medium (Thermo Fisher Scientific). The cells were seeded out in 96-well dark-walled plates at a density of 1 × 10^5^ cells per well. The cells were between second and eighth passage number at the time of the experiment. After 24 h, the cells were loaded with FLIPR Calcium 6 indicator (Molecular Devices, San Jose, CA, USA) for 2 h at 37 °C, according to the manufacturers protocol. Compounds of interest were dissolved in appropriate amount of DMSO, in order to prepare stock solutions at 10 mM concentration. They were then subsequently diluted to a final concentration of 10 μM in Hanks′ Balanced Salt Solution (HBSS, Thermo Fisher Scientific, Waltham, MA, USA) buffered with HEPES (Sigma-Aldrich) at pH = 7.4 and as such used for treatment of indicator loaded cells (10 min at 37 °C). Fluorescence of indicator loaded cells was measured with Synergy H1 (Biotek Instruments, Winooski, VT, USA) multilabel plate reader at excitation and emission wavelengths of 485 and 525 nm, respectively. The baseline fluorescence was recorded for 5 sec. Then, the cells were stimulated with KCl/CaCl_2_ solution (in HBSS, final concentration of KCl and CaCl_2_ was 90 mM and 5 mM, respectively) and the fluorescence was recorded for further 30 s. Dimethyl sulfoxide 1% solution in HBSS was used as a vehicle control. Nimodipine (10 μM) was used as a reference inhibitor. Compounds were assessed at the same final concentration as nimodipine in triplicates in three independent experiments. Fluorescent intensity values were normalized to the baseline. Outlier detection by Grubbs’ test was performed and outlying values were excluded from the further analysis.

#### 3.5.2. Oxygen Radical Absorbance Capacity Assay

The antioxidant activity of hybrids **3a-tI** was carried out by the ORAC-FL using fluorescein as a fluorescent probe. Briefly, fluorescein and antioxidant were incubated in a black 96-well microplate (Nunc, Thermo Scientific, 67403 Illkirch France) for 15 min at 37 °C. 2,2′-Azobis(amidinopropane) dihydrochloride was then added quickly using the built-in injector of a Varioskan Flash plate reader (Thermo Scientific). The fluorescence was measured at 485 nm (excitation wavelength) and 535 nm (emission wavelength) each min for 1h. All the reactions were made in triplicate and at least three different assays were performed for each sample.

#### 3.5.3. hMAOs Inhibition Screening

The effect of the test compounds on the activity of both hMAO isoforms was evaluated by measuring the production of 4-hydroxyquinoline (4-HQ, λ_max_= 316 nm) from kynuramine, using microsomal recombinant hMAO isoforms. Prior to the each experiment, stock solutions were prepared in the following conditions: test compounds/standard inhibitors (2 mM in DMSO), kynuramine (400 µM in dH_2_O), hMAO-A (36.7 µg/mL in sodium phosphate buffer 0.05 M pH 7.4,) and hMAO-B (147.8 µg/mL in sodium phosphate buffer 0.05 M pH 7.4,). The amount of hMAO-A (specific activity= 116 pmol kynuramine/min/µg protein) and hMAO-B (specific activity = 16.4 pmol kynuramine/min/ µg protein) was adjusted to obtain the same reaction rate (50 pmol kynuramine/min) under our experimental conditions. The assays were run in flat bottom 96-well microplates (BRANDplates, Pure Grade^TM^, BRAND GMBH, Wertheim, Germany) using a multimode plate reader (Biotek Synergy HT). Each experiment included control wells (vehicle) and standard inhibitors (clorgyline for hMAO-A and deprenyl for hMAO-B). In a typical experiment, 1 µL the test compounds/standard inhibitors (test wells) or DMSO (control wells), 20 µL of kynuramine and 160 µL of sodium phosphate buffer (0.05 M, pH 7.4) were incubated at 37 °C for 10 min and the absorbance at 316 nm was read to correct the background signal. After this incubation, 20 µL of hMAO-A or hMAO-B were added and the absorbance at 316 nm was read each minute over 45 min. Using a previously determined calibration curve for 4-HQ, the amount of 4-HQ formed was calculated and the n (4-HQ)= f (t) plots were obtained for each treatment. The reaction rates (ν_0_, indicted by the slopes) were used to calculate the % of inhibition, according to the following formula: % inhibition = [ν_0_ (control) - ν_0_ (test)]/ ν_0_ (control) * 100. The IC_50_ of the standard inhibitors were determined using the same protocol, using increasing concentrations of chlorgyline and deprenyl (0–100 nM) and tracing the dose-response curves. All values (% inhibition and IC_50_) are the mean ± SD from three experiments.

#### 3.5.4. Effect of Compounds 3a, 3h and 3j on H_2_O_2_ (200 µM) and Oligomycin/Rotenone (O at 10µM and R at 30 µM)-Induced Cell Death in SH-SY5Y Cells

SH-SY5Y cells were seeded in 96-well culture plates at a density of 12 × 10^4^ cells per well in DMEM/F12 (1:1) medium supplemented with 10% fetal bovine serum, 1× non-essential amino acids, 100 units/mL penicillin and 10 mg/mL streptomycin (Dutscher, 67170 Brumath, France). After 48 h of incubation, the cultures were treated with 100 µL of the test compounds or DMSO (0.1%) in the same medium. Following 24 h, the cells were co-incubated with H_2_O_2_ (200 µM) or a mixture of oligomycin/rotenone (10 µM/ 30 µM) with or without the tested compounds at noncytotoxic concentrations for an additional period of 24 h. The percent of cell viability was measured using CellTiter 96 AQueous Non-Radioactive Cell Proliferation (MTS) Assay (Promega, Charbonnières-les-Bains, France).

## 4. Conclusions

Compounds **3a**–**t** have been successfully synthesized in modest to high yields by Hantzsch multicomponent reactions, and their biological evaluation, as potential MAO inhibitors, Ca^2+^ channel blockers, antioxidant and neuroprotective agents has been assessed. Unfortunately, our molecules displayed very low MAO inhibition power. However, concerning Ca^2+^ channel blockade results, it is worth mentioning that compounds **3a** and **3h** showed Ca^2+^ influx blockage values of 47%, and 42.8%, respectively, very close to the standard reference nimodipine (52.8%) at 10 μM. The most active compounds were those with n = 1 linker length, with R^1^= H (**3a**) and R^1^= Cl (**3h**). This suggests that the C3 position at the aryl core is involved in the Ca^2+^ channel blockade, and that this finding is a good strategy for further pharmacomodulation studies. The antioxidant activity allowed us to identify molecules **3l** (2.45 TE), **3n** (2.78 TE) and **3p** (2.78 TE) as interesting antioxidants with ORAC values equal or higher than melatonin (2.45TE). Moreover, and very interestingly, the most potent Ca^2+^ channel agonists, **3a** and **3h**, showed interesting ORAC values equal to 1.75TE and 1.35TE. The neuroprotective activity results support the interest of compounds **3a** and **3h**, particularly for their neuroprotective activity against H_2_O_2_ in SHSY5Y cells. To sum up, the biological analyses have prompted us to identify the multifunctional compound 3,5-dimethyl-2,6–dimethyl–4-[4-(prop–2–yn–1-yloxy)phenyl]-1,4-dihydropyridine-3,5-dicarboxylate (**3a**) as an attractive antioxidant (1.75 TE), Ca^2+^ channel antagonist (47.0% at 10 μM), showing significant neuroprotection (38%) against H_2_O_2_ at 10 μM, being considered thus as a new hit-compound for further investigation in our search for anti-Alzheimer’s disease agents. These preliminary results confirmed in part the efficiency of our design. Thus, based on the poor MAO inhibition results, we intend to design and prepare new compounds witn nitrogen instead of oxygen at the aromatic ring, that we hope and expect to be more appropriate for efficient MAO inhibition. This work is now in progress in our laboratories, and the results will be presented elsewhere in due course.

## Figures and Tables

**Table 1:** Calcium blockade percentages for compounds **3a**–**t**, expressed in percentage of inhibition at 10 μM, and their ORAC (TE) values.^a.^

Compounds	n	R^1^	R^2^	Ca^2+^ Channel inhib. at 10 μM (%)	ORAC ^b^
**3a**	1	H	CH_3_	47.0 ± 6.6	1.75 ± 0.07
**3b**	1	H	CH_2_CH_3_	33.2 ± 3.9	1.44 ± 0.07
**3c**	1	OCH_3_	CH_3_	- ^c^	1.30 ± 0.02
**3d**	1	OCH_3_	CH_2_CH_3_	33.3 ± 9.5	1.60 ± 0.16
**3e**	1	OCH_2_CH_3_	CH_3_	- ^c^	0.502 ± 0.01
**3f**	1	OCH_2_CH_3_	CH_2_CH_3_	22.9 ± 6.7	0.98 ± 0.04
**3g**	1	Cl	CH_3_	28.7 ± 4.3	1.05 ± 0.05
**3h**	1	Cl	CH_2_CH_3_	42.8 ± 14.0	1.35 ± 0.05
**3i**	1	Br	CH_3_	31.3 ± 5.8	0.84 ± 0.03
**3j**	1	Br	CH_2_CH_3_	39.0 ± 4.8	1.41 ± 0.07
**3k**	2	H	CH_3_	20.2 ± 3.5	1.85 ± 0.17
**3l**	2	H	CH_2_CH_3_	22.5 ± 9.8	2.45 ± 0.25
**3m**	2	OCH_3_	CH_3_	22.4 ± 6.8	1.57 ± 0.14
**3n**	2	OCH_3_	CH_2_CH_3_	- ^c^	2.78 ± 0.17
**3o**	2	OCH_2_CH_3_	CH_3_	24.6 ± 2.2	2.06 ± 0.03
**3p**	2	OCH_2_CH_3_	CH_2_CH_3_	26.9 ± 1.1	2.77 ± 0.03
**3q**	2	Cl	CH_3_	- ^c^	1.06 ± 0.11
**3r**	2	Cl	CH_2_CH_3_	27.0 ± 5.8	0.83 ± 0.02
**3s**	2	Br	CH_3_	- ^c^	0.62 ± 0.03
**3t**	2	Br	CH_2_CH_3_	24.2 ± 3.7	1.43 ± 0.01
**Nimodipine**	-			52.8 ± 5.5	nd ^d^
**Melatonin**				nd ^d^	2.45 ± 0.09

^a^ Every percentage value is the mean of a triple of at least two different experiments. ^b^ Data are expressed as Trolox equivalents and are the mean (*n* = 3) ± SEM. ^c^ not active. ^d^ nd: not determined.

**Table 2:** Neuroprotective activity of compounds **3a**, **3h** and **3j** on H_2_O_2_ (200 µM) or oligomycin (O at 10 µM) /rotenone (R at 30 µM)-induced cell death in SH-SY5Y cells ^a^.

Compounds	Concentration	H_2_O_2_ (%)	O/R (%)
**3a**	0.3 µM	22.49 +/− 0.03 *	21.66 +/− 0.02 *
	10 µM	38.15 +/− 0.04 *	11.21 +/− 0.04
**3h**	0.3 µM	12.80 +/− 0.01	np ^b^
	10 µM	7.58 +/− 0.03	np ^b^
**3j**	0.3 µM	19.34 +/− 0.03 *	14.59 +/− 0.03
	10 µM	39.78 +/− 0.08 *	np ^b^

^a^ Data are expressed as % neuroprotection ± SEM of quadruplicates from three different cultures; * *p*  <  0.05, as compared to the control cultures (one-way ANOVA); np: not protective.

## Supplementary Materials

The following are available online, Figures are NMR Spectra of compounds 3a–t.
