# Supplementary material for: Design, Synthesis and Biological Evaluation of New Antioxidant and Neuroprotective Multitarget Directed Ligands Able to Block Calcium Channels
Source: Molecules. 2020 Mar 14;25(6):1329. doi: 10.3390/molecules25061329 (PMC7144121; doi:10.3390/molecules25061329)

Supplementary Material

Design, Synthesis and Biological Evaluation of New Antioxidant and Neuroprotective Multitarget Directed Ligands able to Block Calcium Channels

Irene PACHON ANGONA[1], Solene DANIEL[1], Helene MARTIN[2] , Alexandre BONET[2], Artur WNOROWSKI[3], Maciej MAJ[3], Krzysztof JOZWIAK[3], Tiago BARROS SILVA[4], Bernard REFOUVELET[1], Fernanda BORGES[4], José MARCO-CONTELLES*,[5] and Lhassane ISMAILI*[2]

[1]Neurosciences intégratives et cliniques EA 481, Pôle de Chimie Organique et Thérapeutique, Univ. Bourgogne Franche-Comté, UFR Santé, 19, rue Ambroise Paré, F-25000 Besançon, France

[2]PEPITE EA4267, Laboratoire de Toxicologie Cellulaire, Univ. Bourgogne Franche-Comté, F-25000 Besançon, France

[3]Department of Biopharmacy, Medical University of Lublin, ul. W. Chodzki 4a, 20-093 Lublin, Poland

[4]CIQUP/Department of Chemistry and Biochemistry, Faculty of Sciences, University of Porto, R. Campo Alegre 1021/1055, 4169-007 Porto, Portugal
[5]Laboratory of Medicinal Chemistry (IQOG, CSIC), Juan de la Cierva, 3; 28006-Madrid, Spain

NMR Spectra of compounds 3a-t……………………………………………………………S2-S22

1HNMR spectra of compound 3a

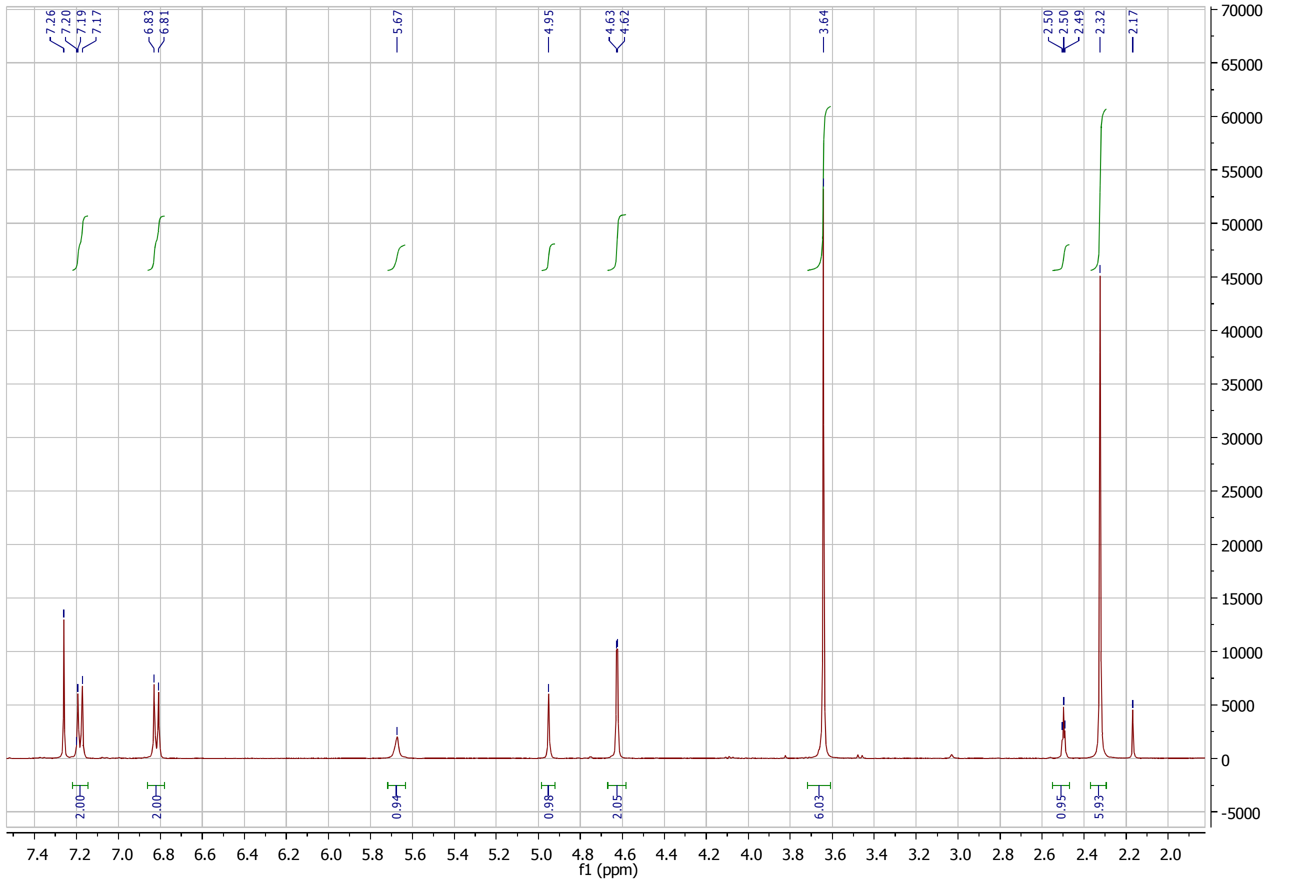

13CNMR spectra of compound 3a

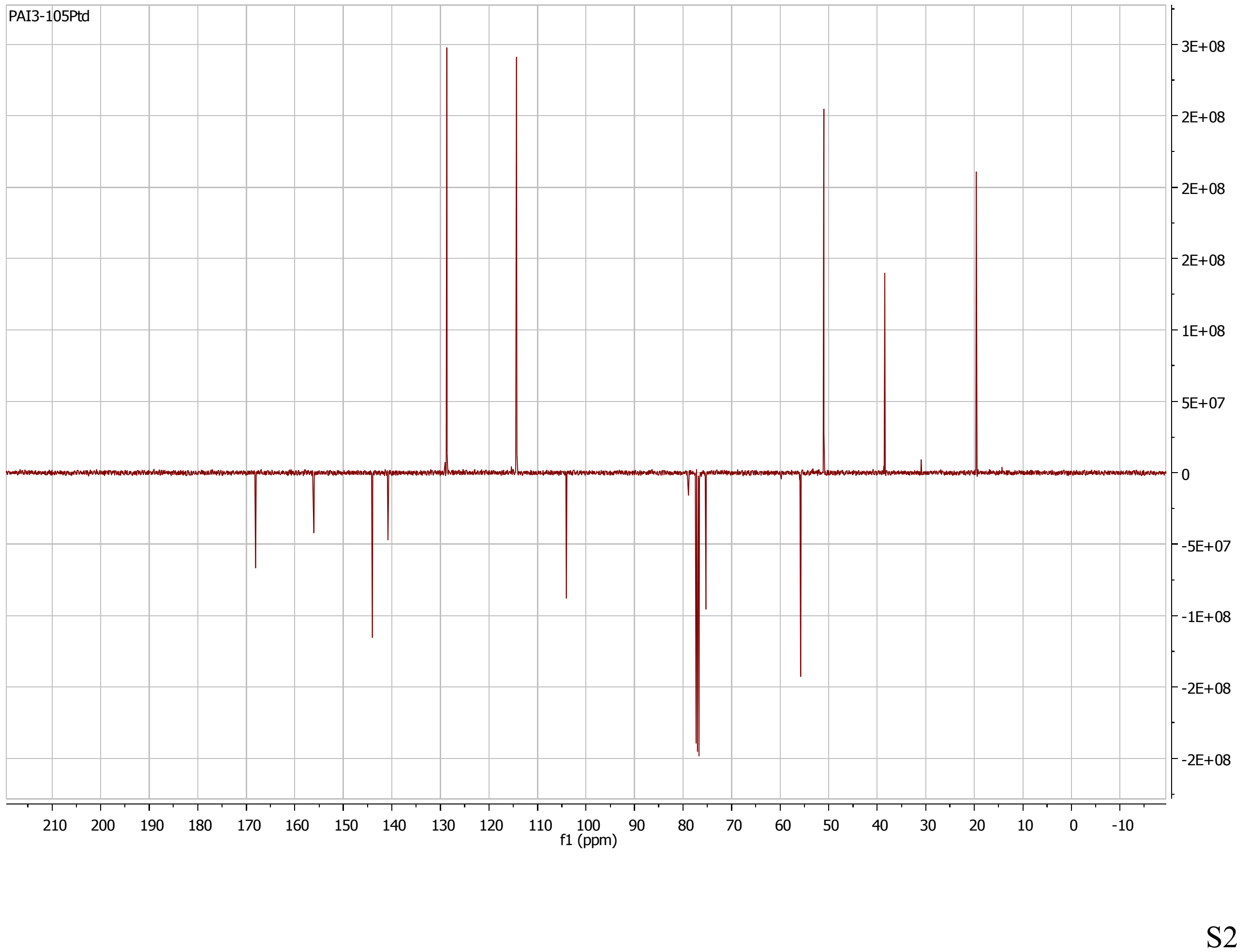

1HNMR spectra of compound 3b

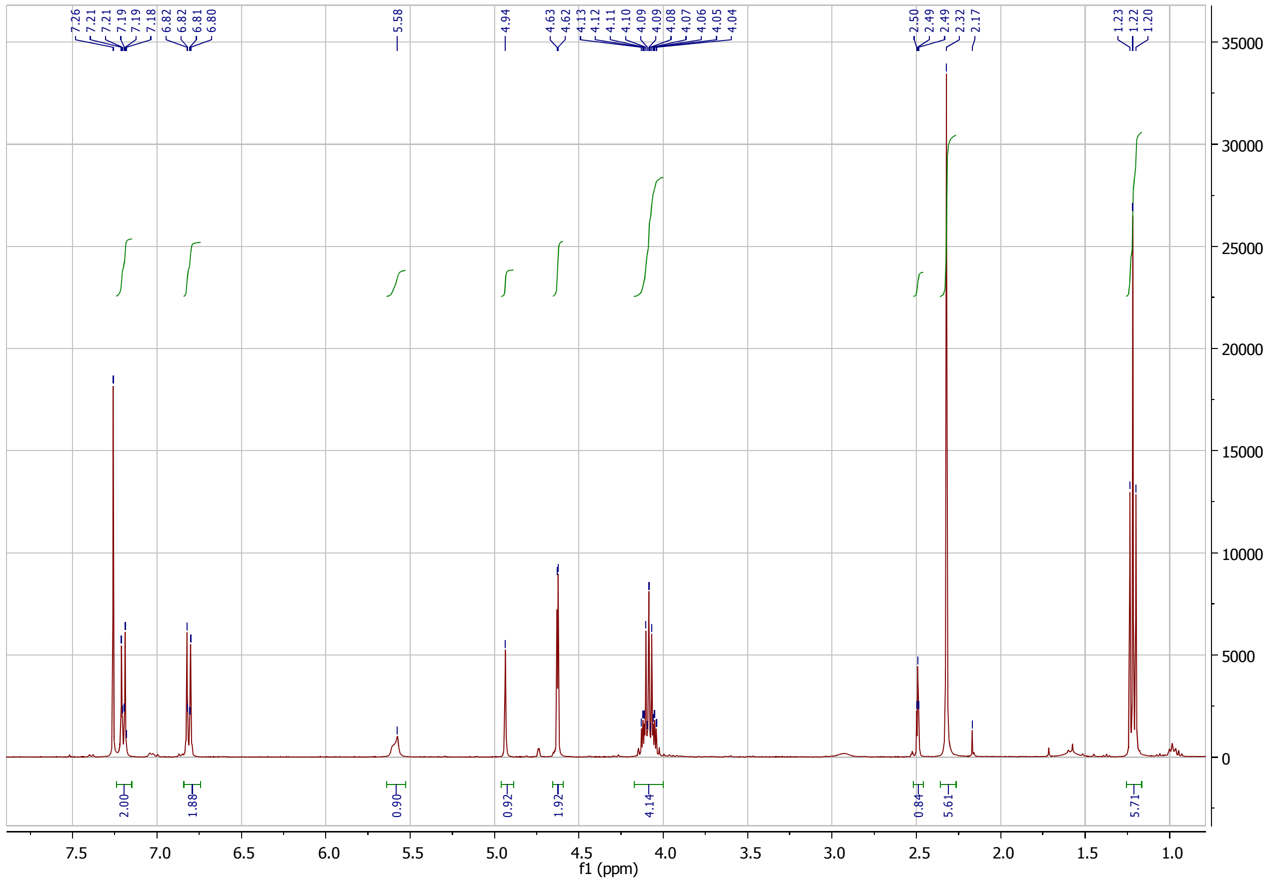

13CNMR spectra of compound 3b

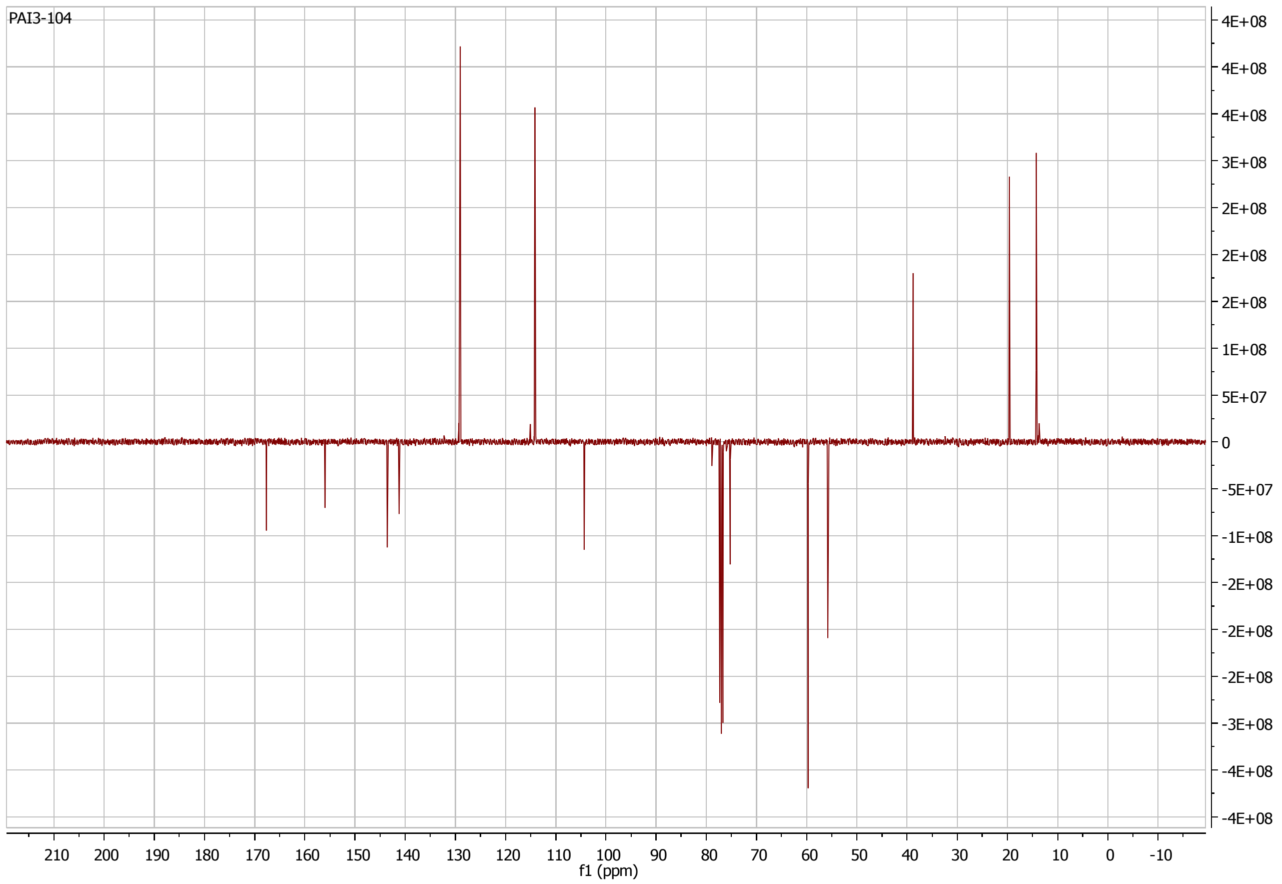

1HNMR spectra of compound 3c

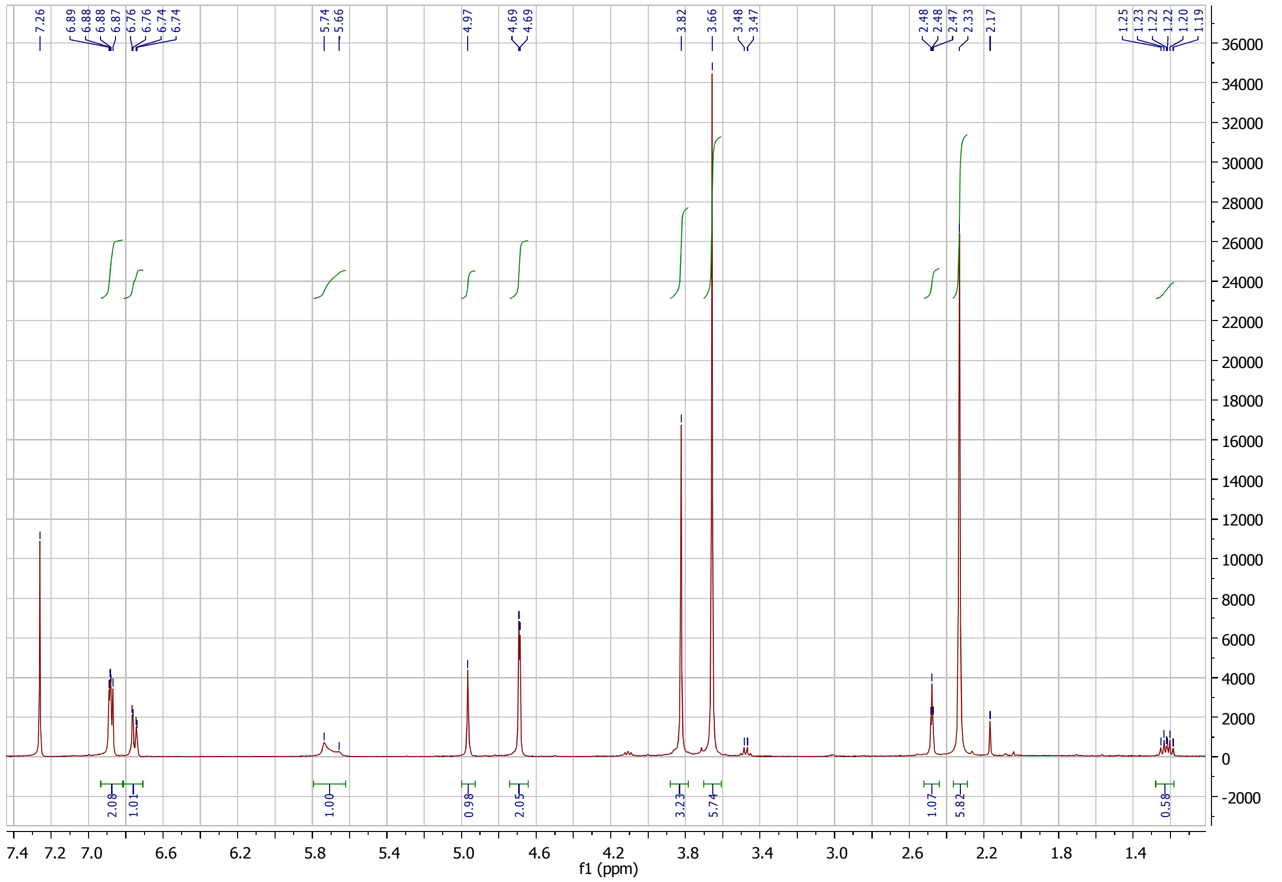

13CNMR spectra of compound 3c

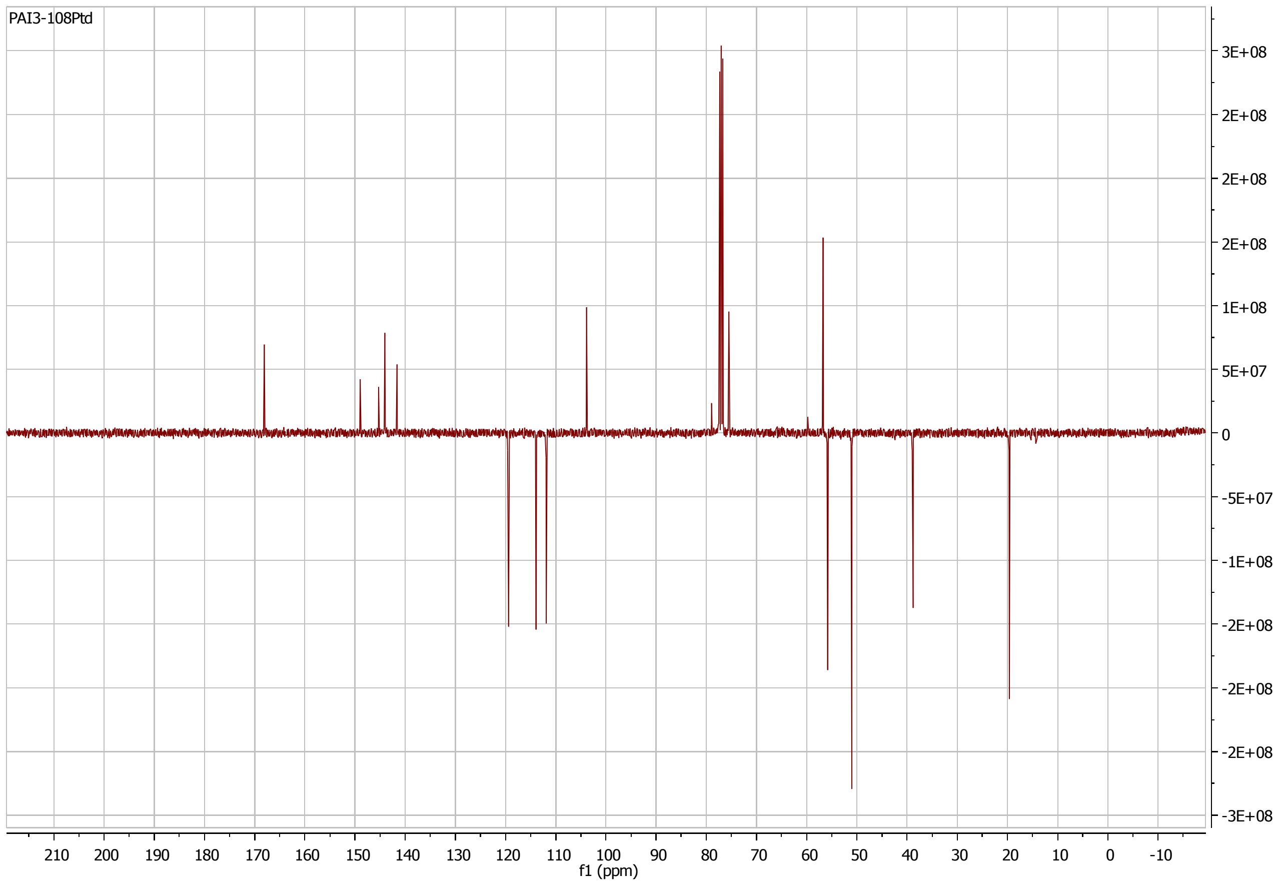

1HNMR spectra of compound 3d

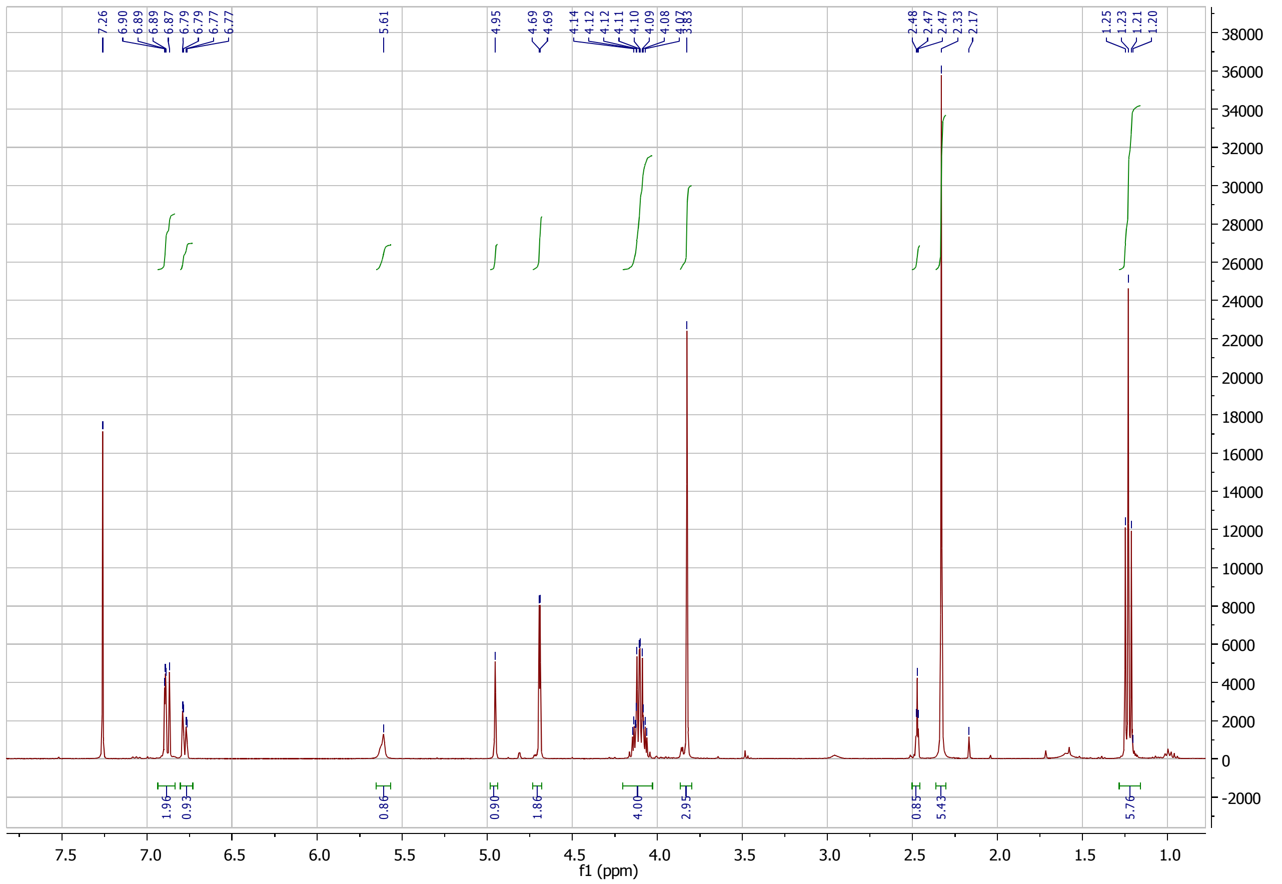

13CNMR spectra of compound 3d

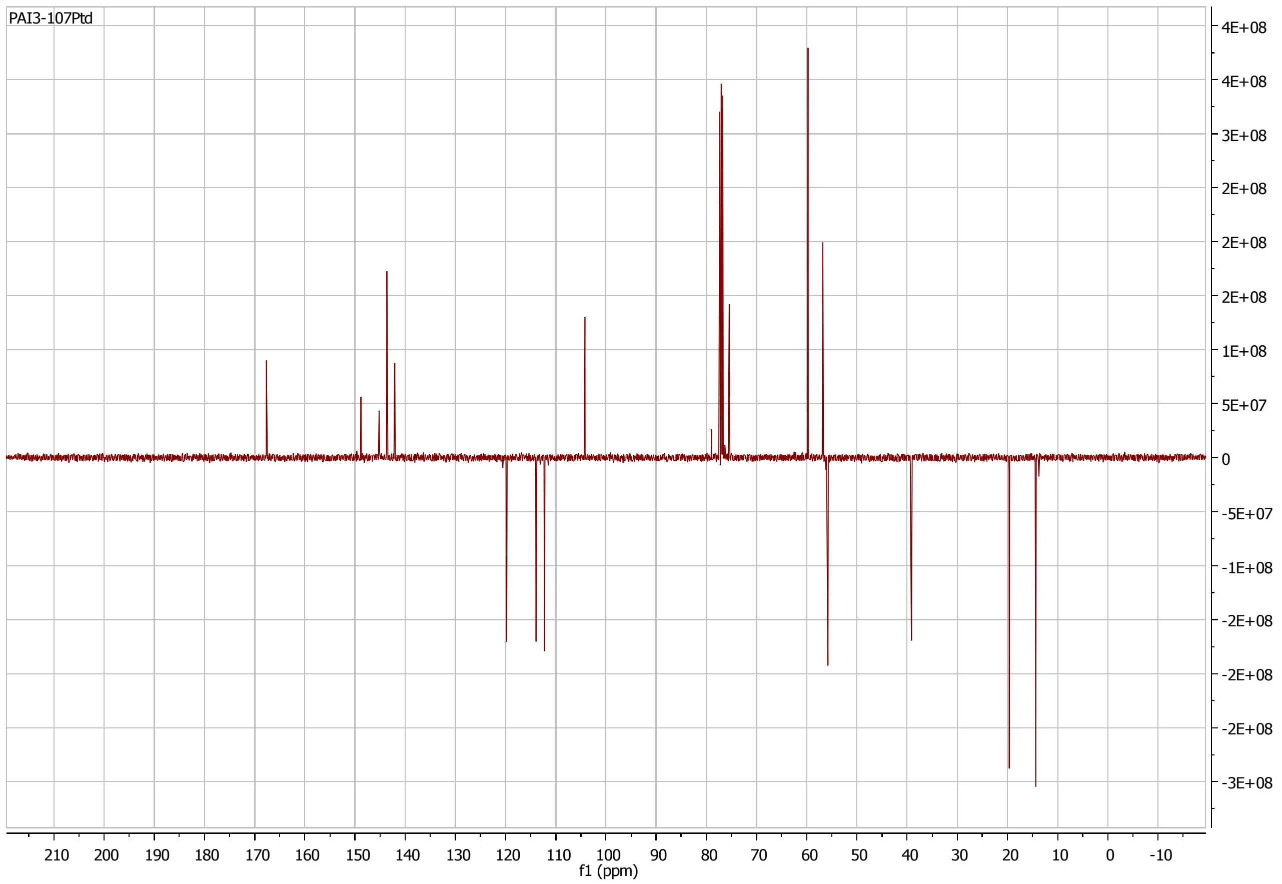

1HNMR spectra of compound 3e

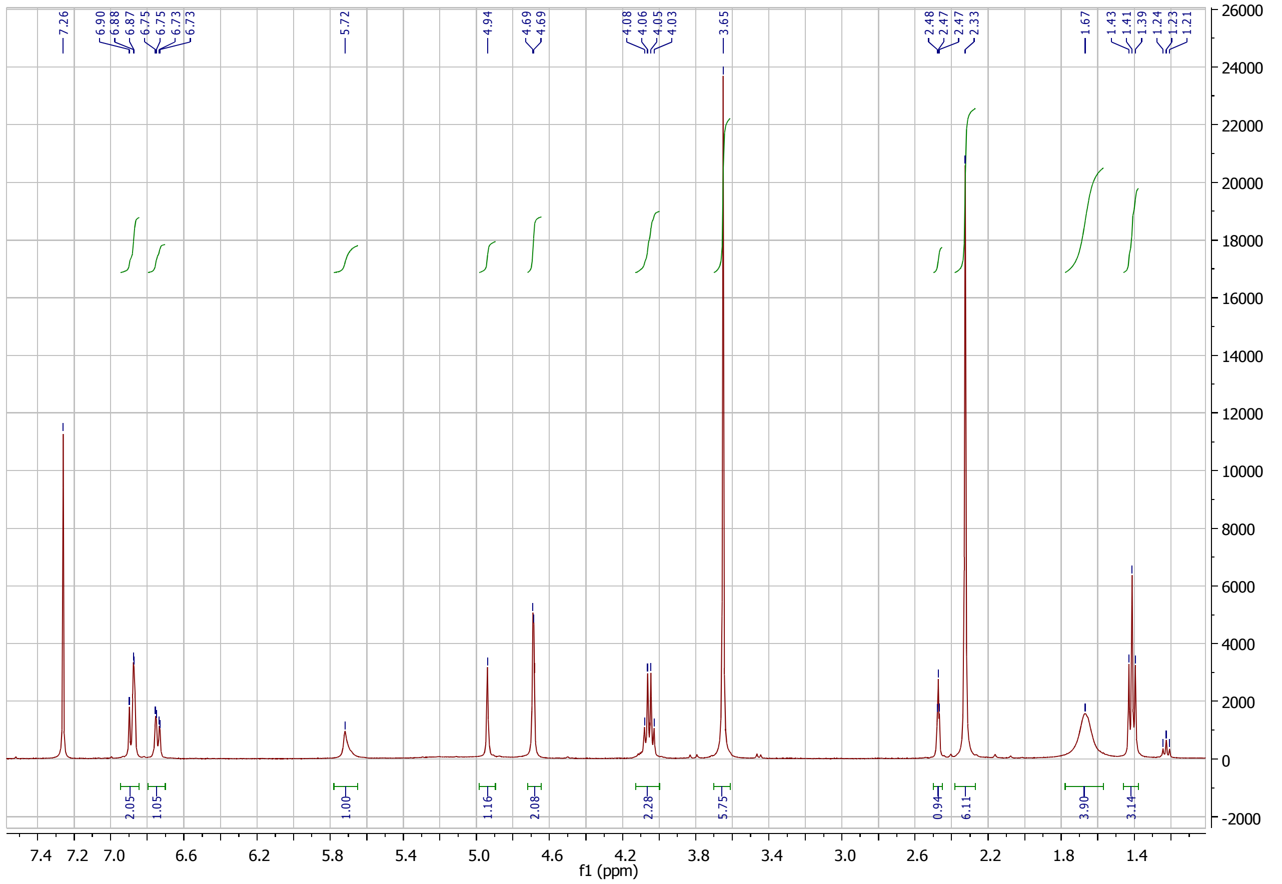

13CNMR spectra of compound 3e

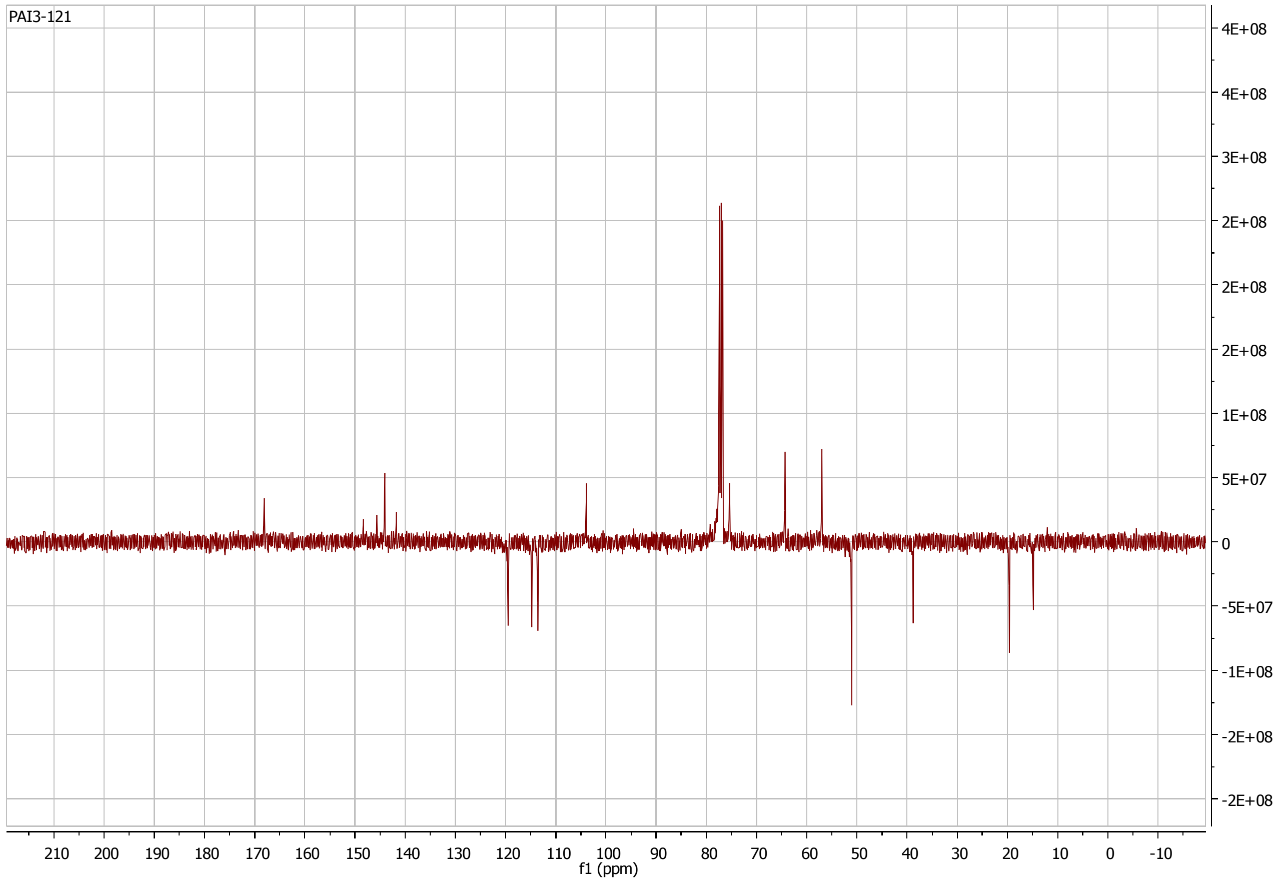

1HNMR spectra of compound 3f

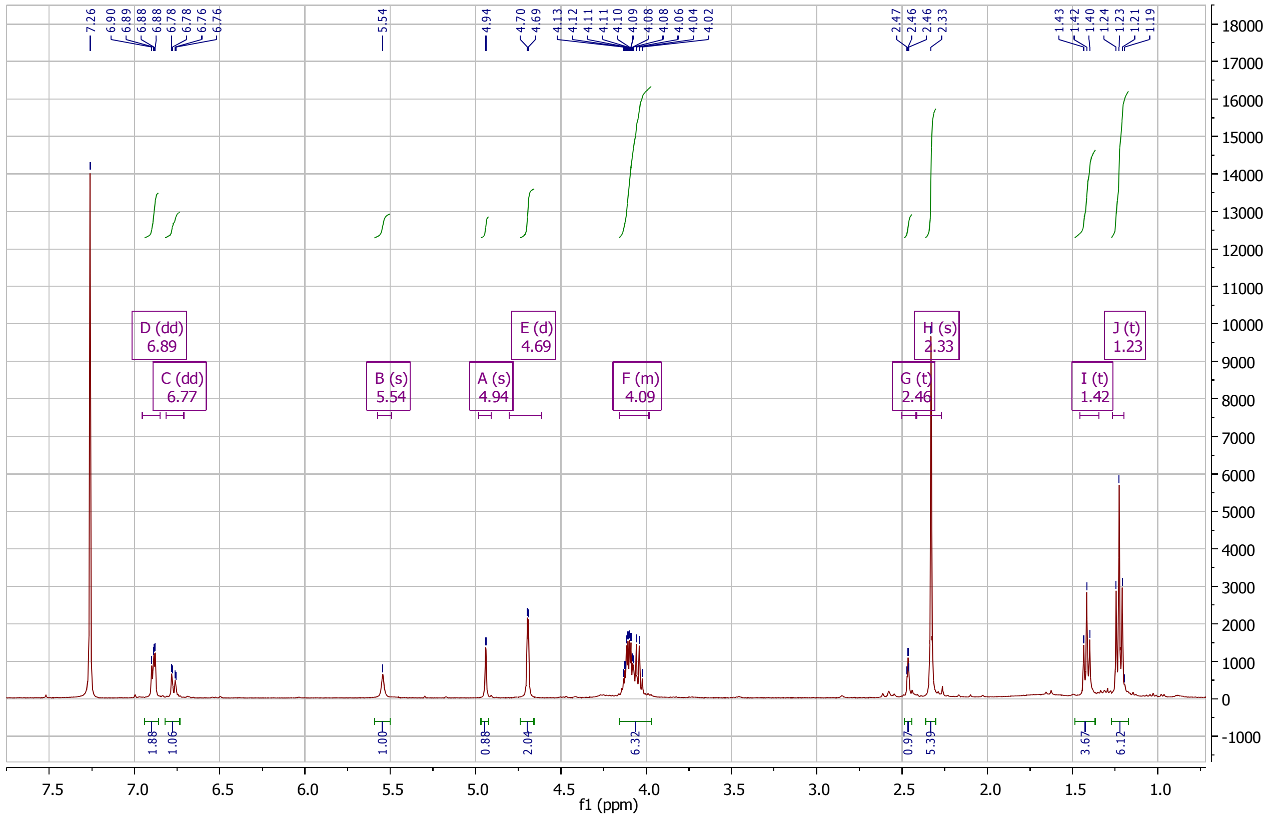

13CNMR spectra of compound 3f

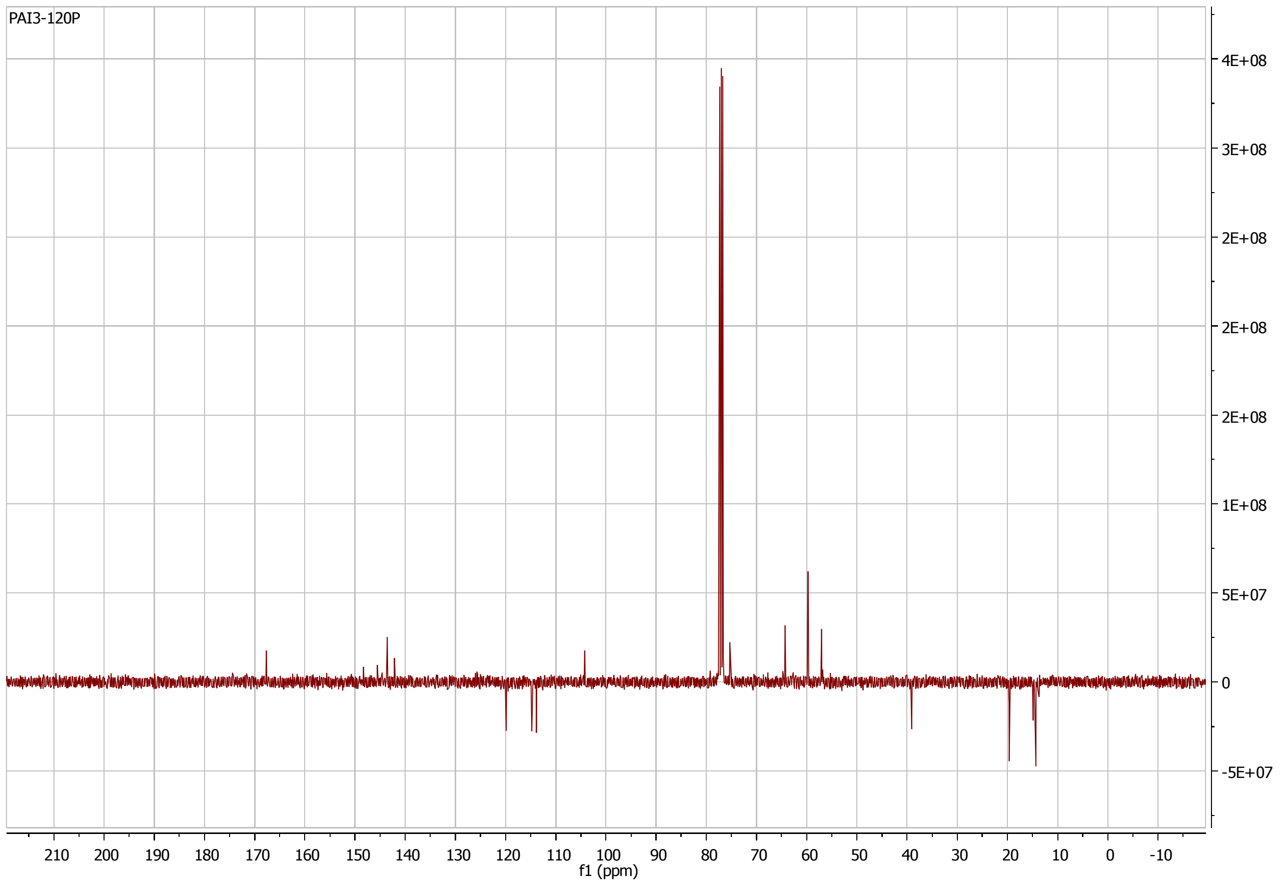

1HNMR spectra of compound 3g

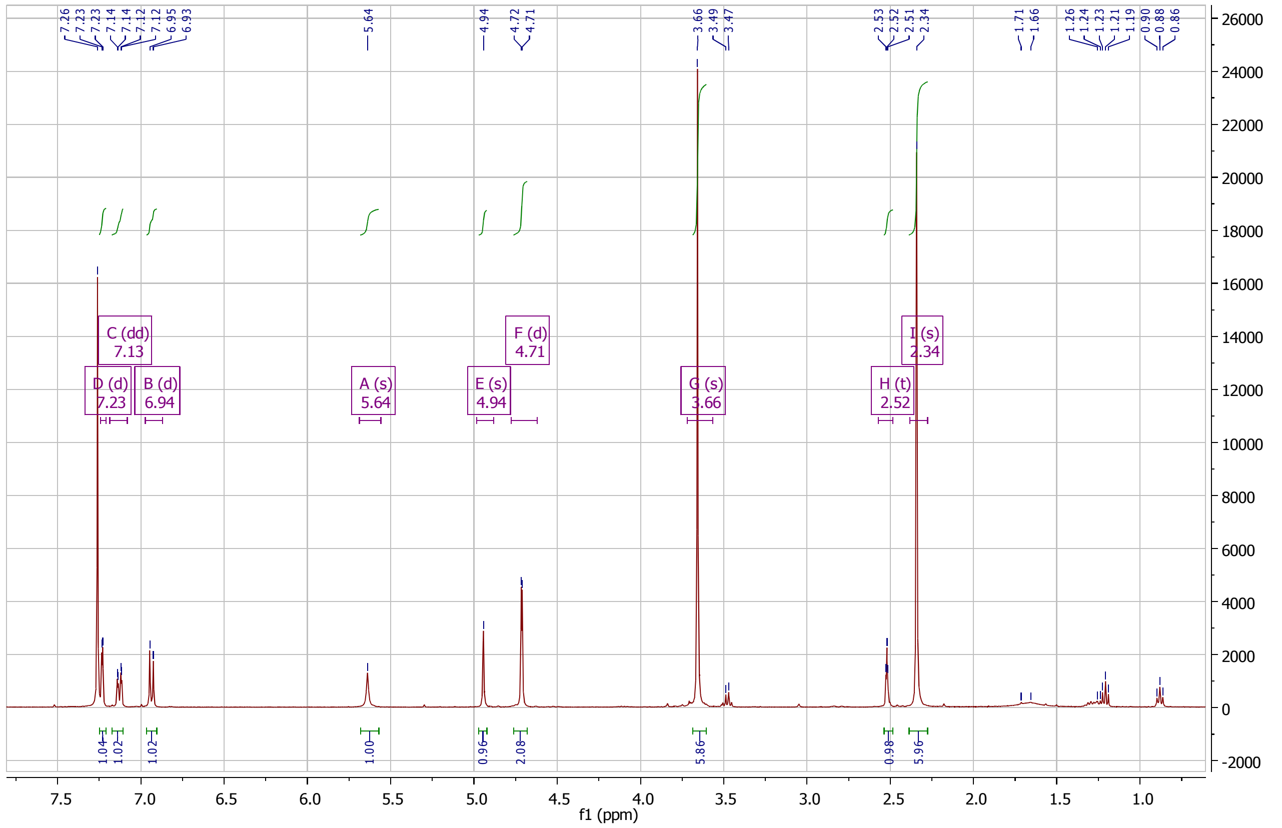

13CNMR spectra of compound 3g

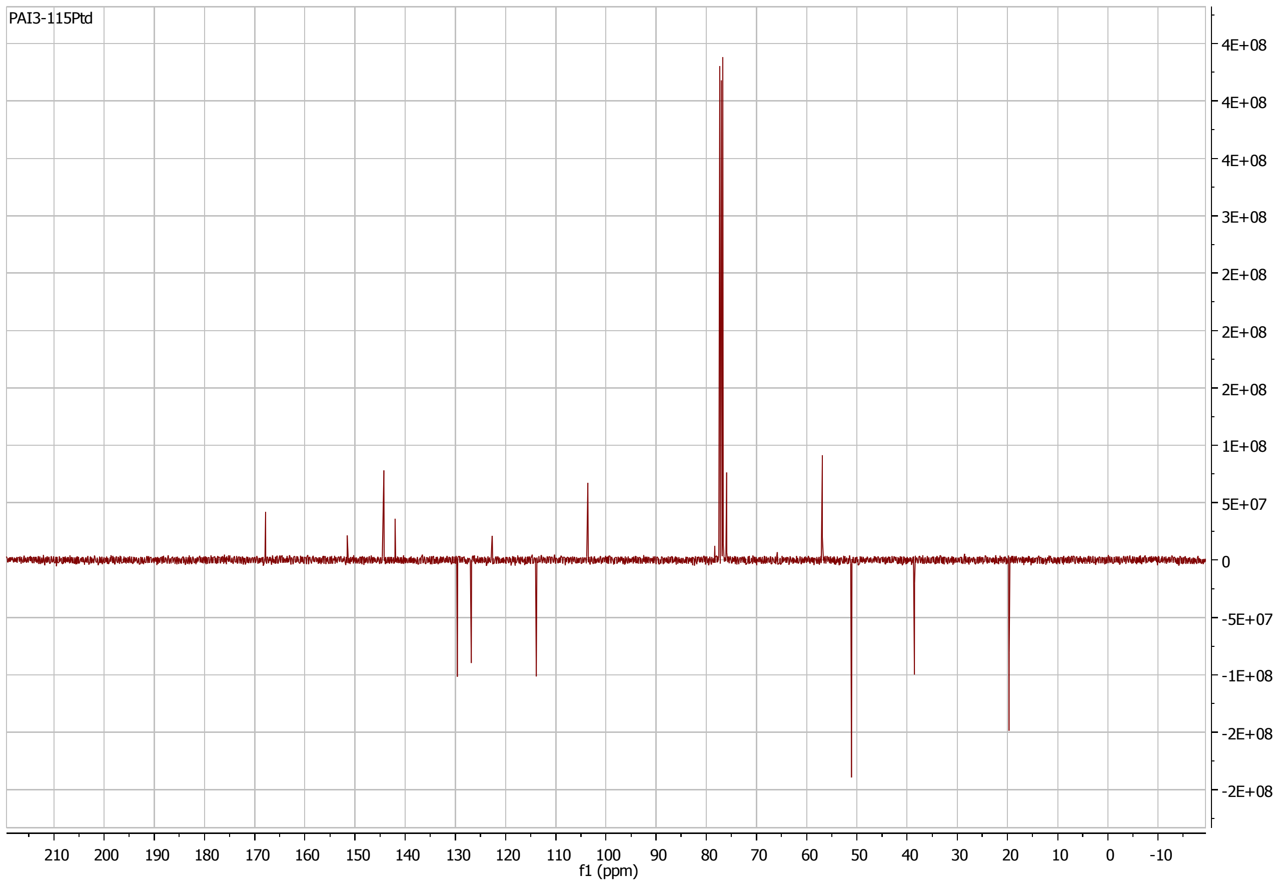

1HNMR spectra of compound 3h

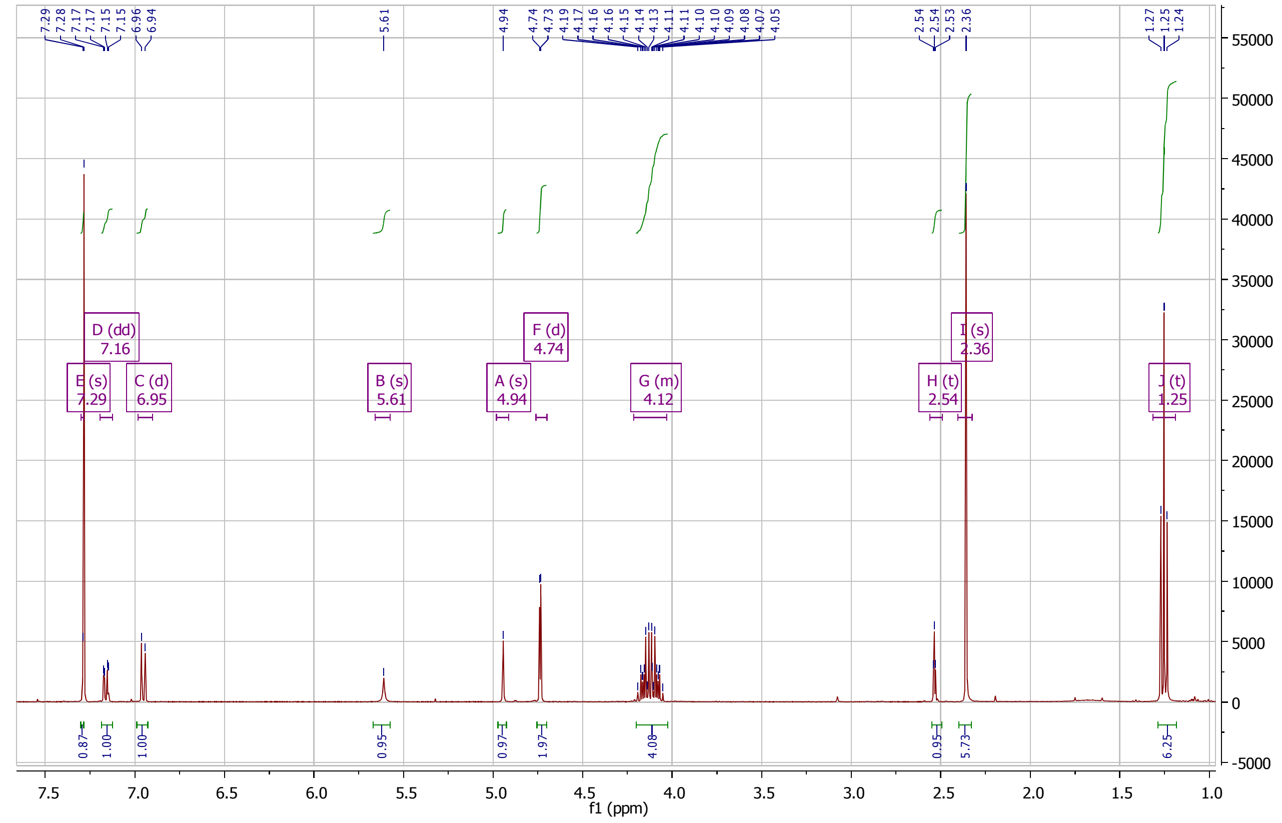

13CNMR spectra of compound 3h

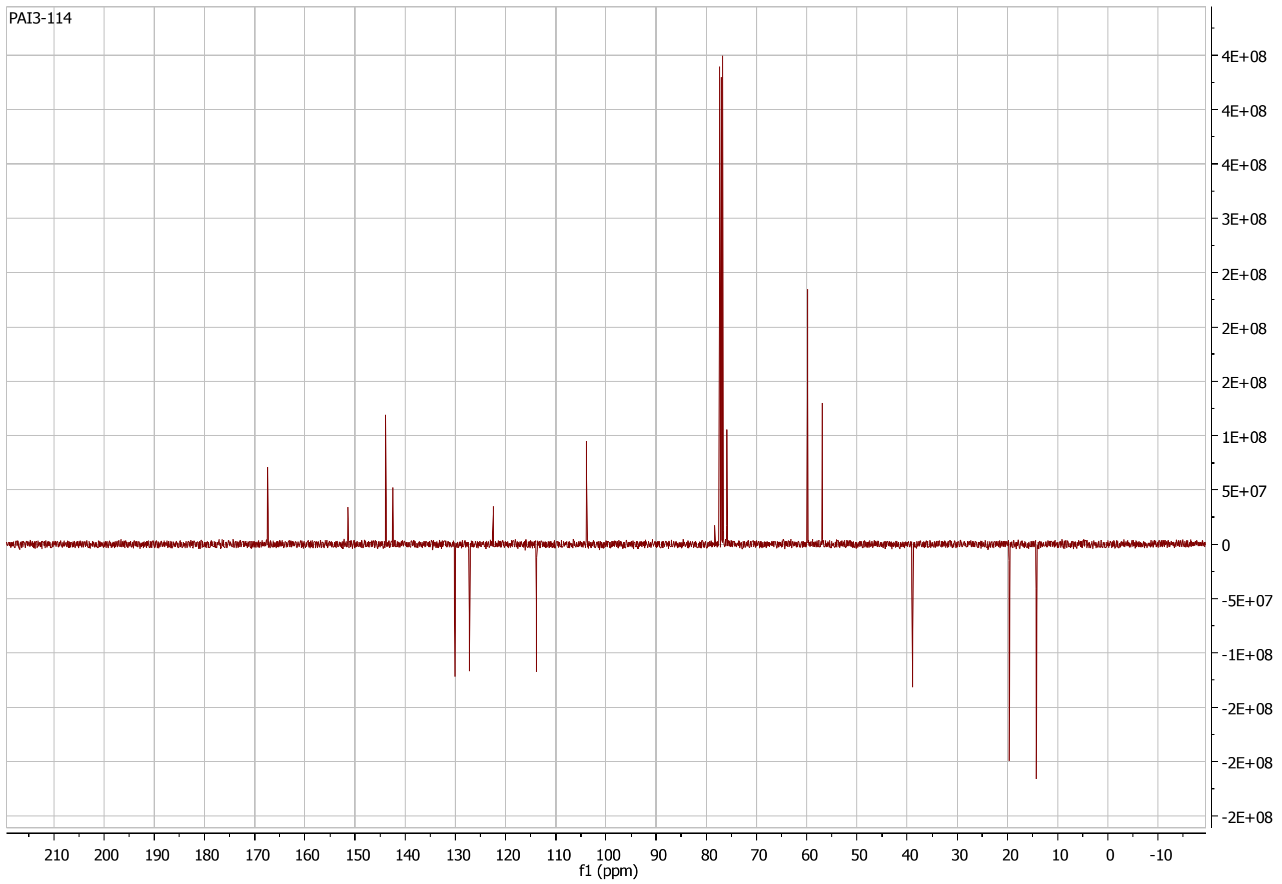

1HNMR spectra of compound 3i

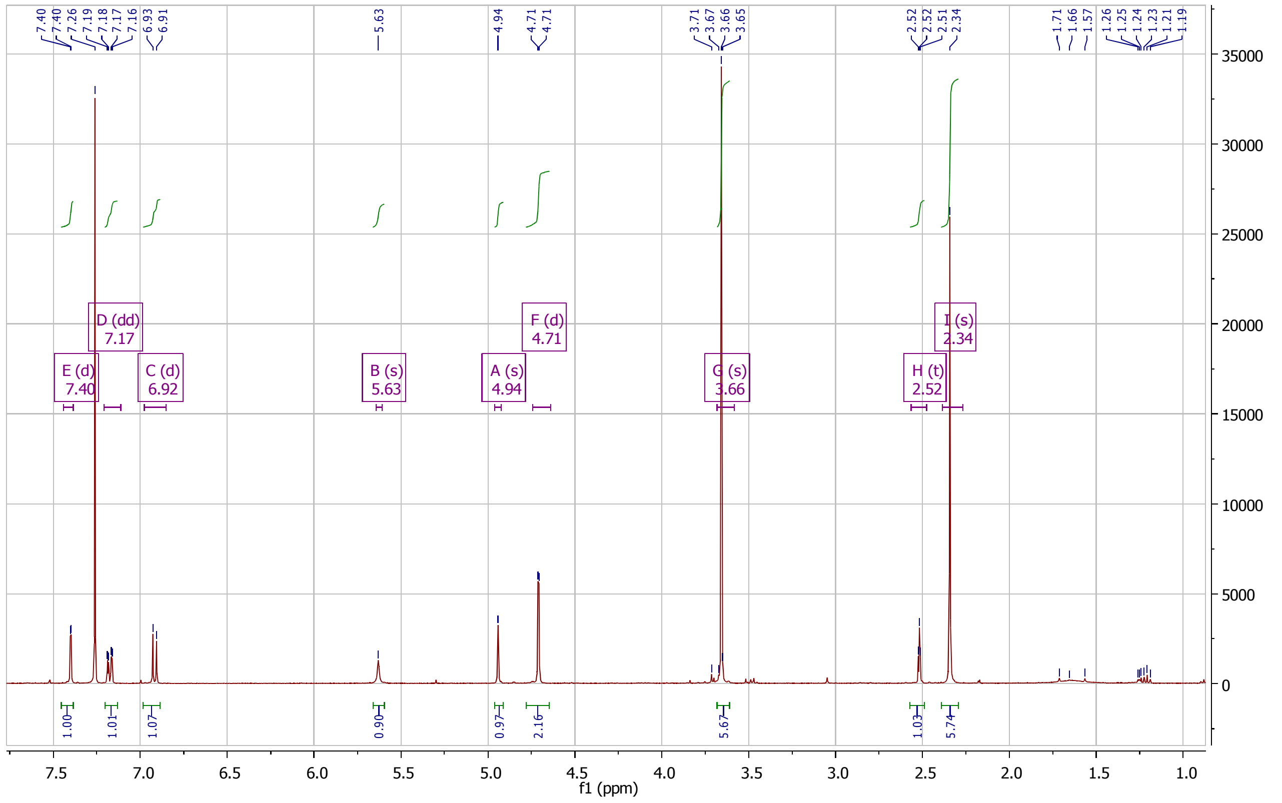

13CNMR spectra of compound 3i

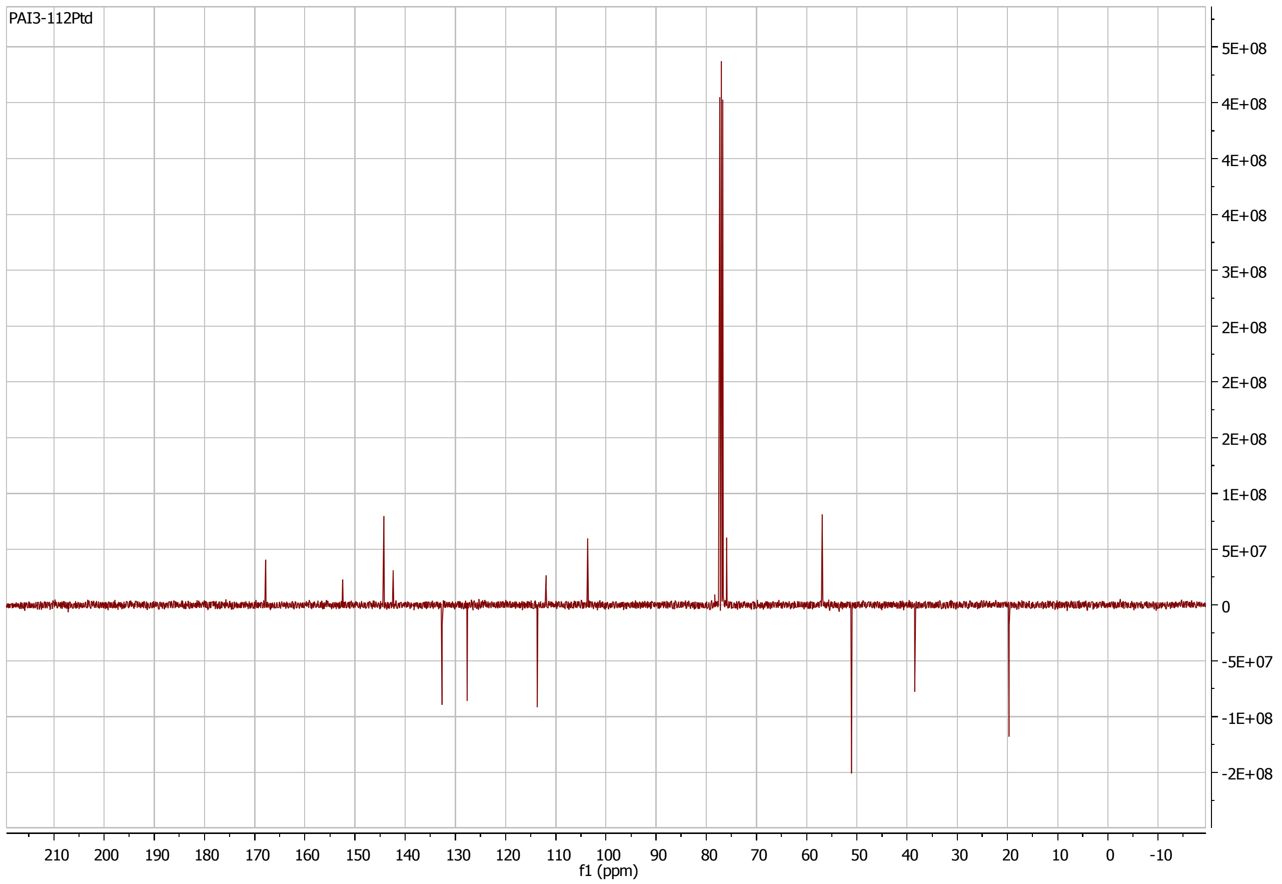

1HNMR spectra of compound 3j

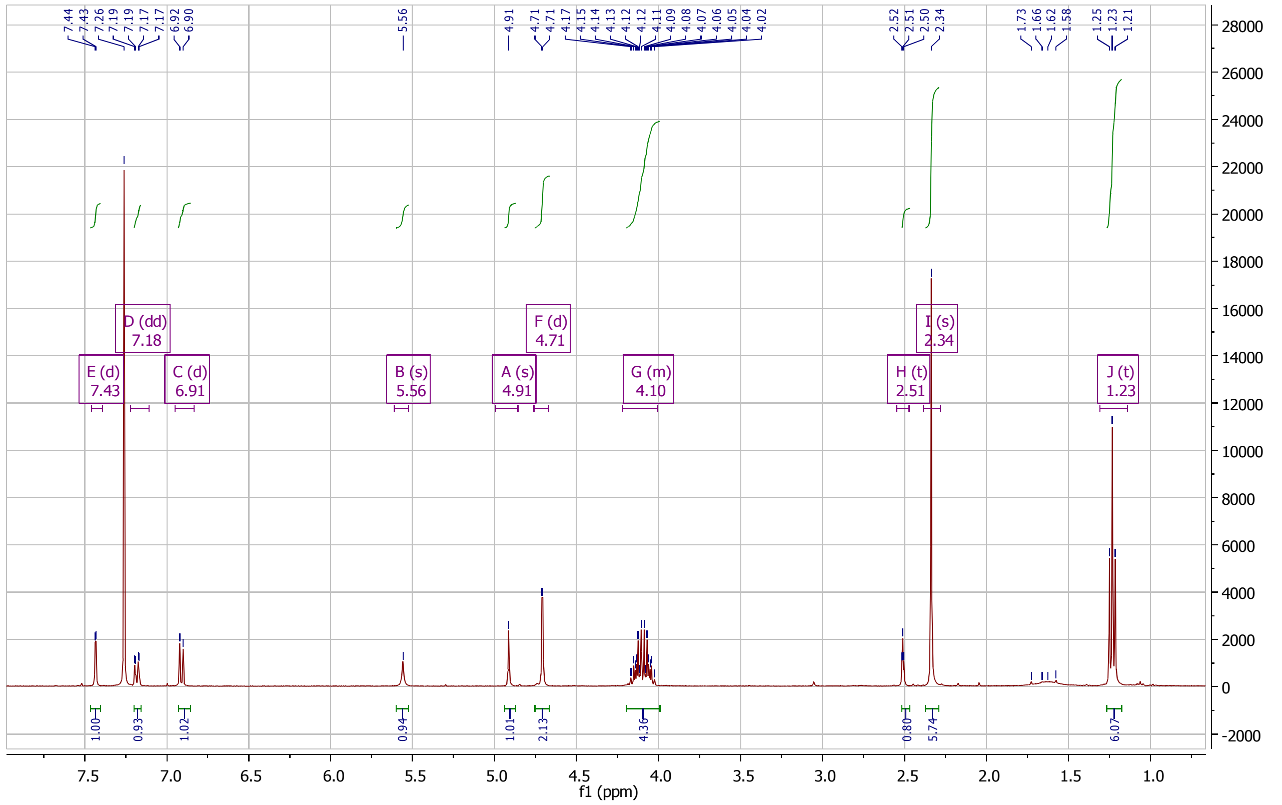

13CNMR spectra of compound 3j

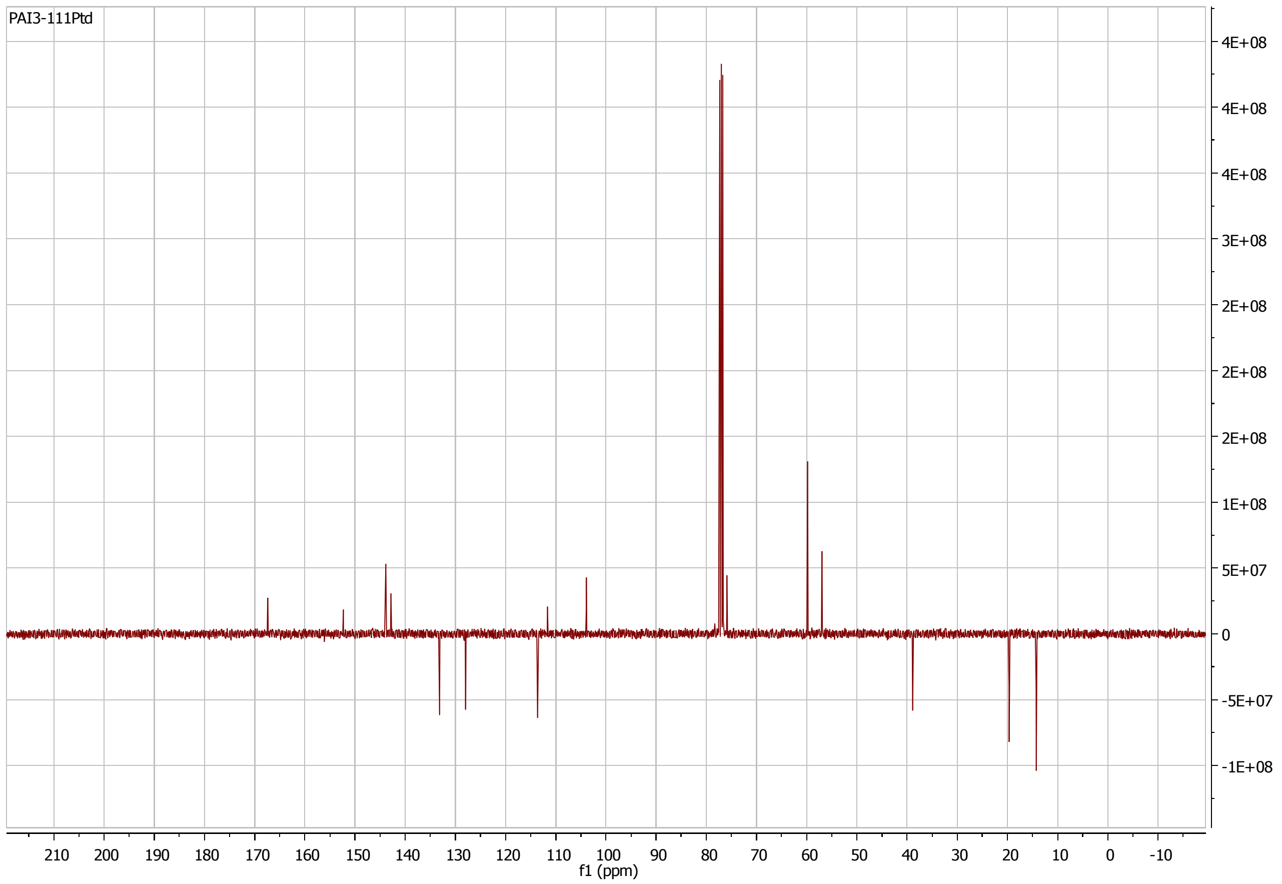

1HNMR spectra of compound 3k

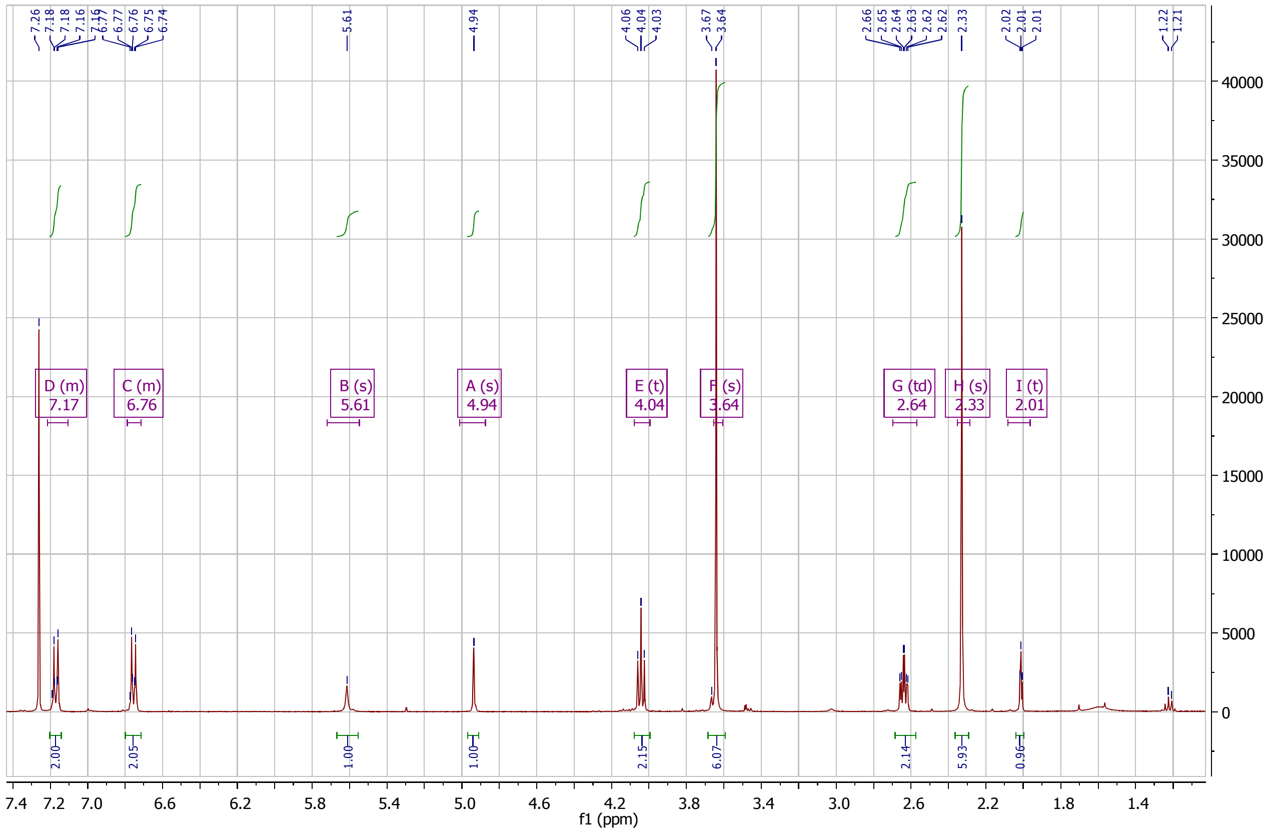

13CNMR spectra of compound 3k

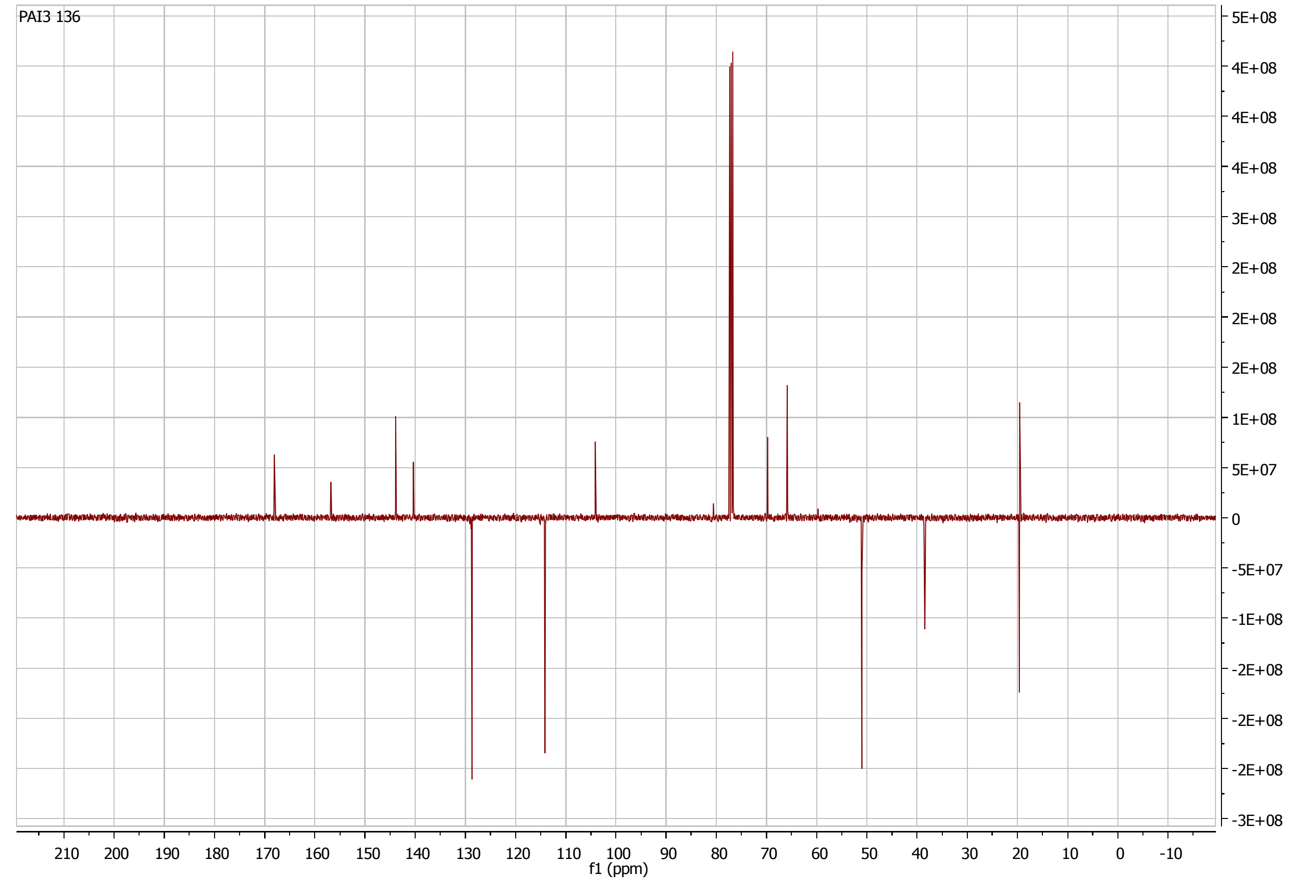

1HNMR spectra of compound 3l

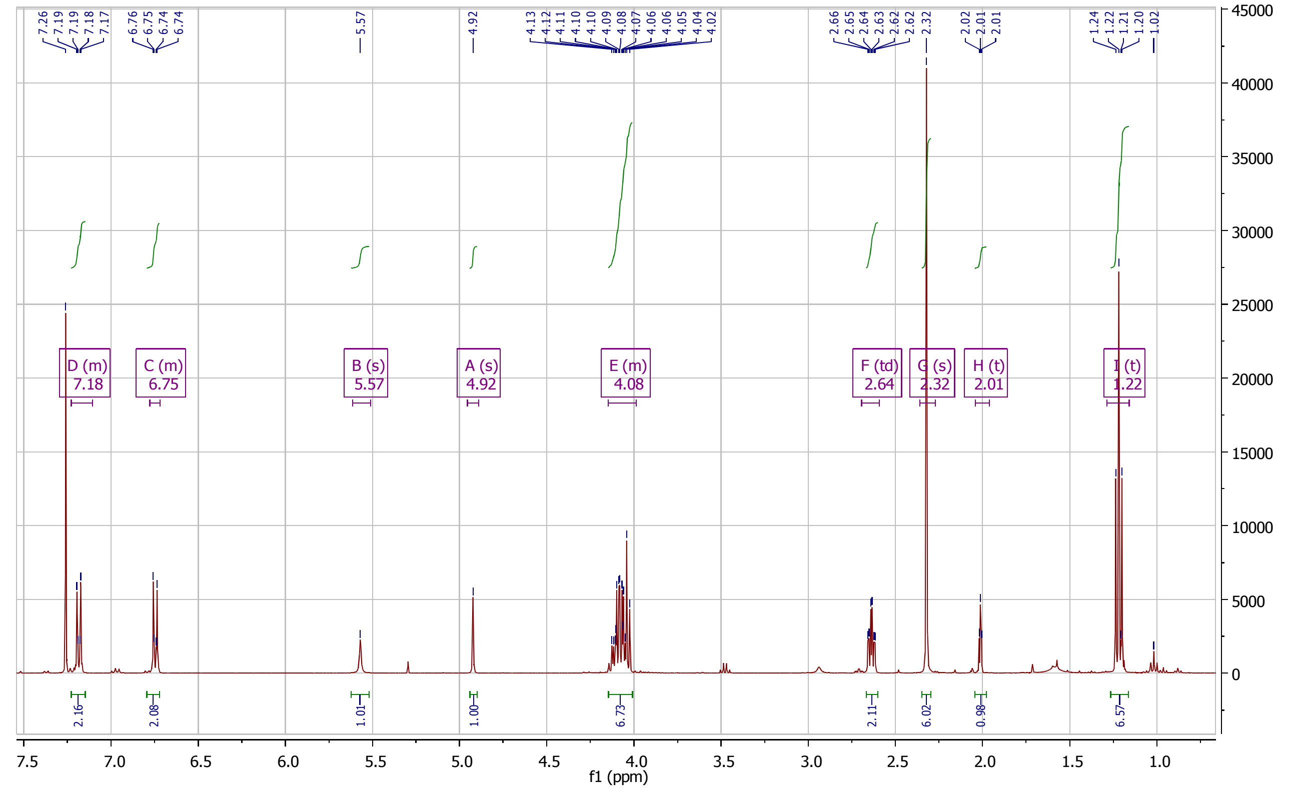

13CNMR spectra of compound 3l

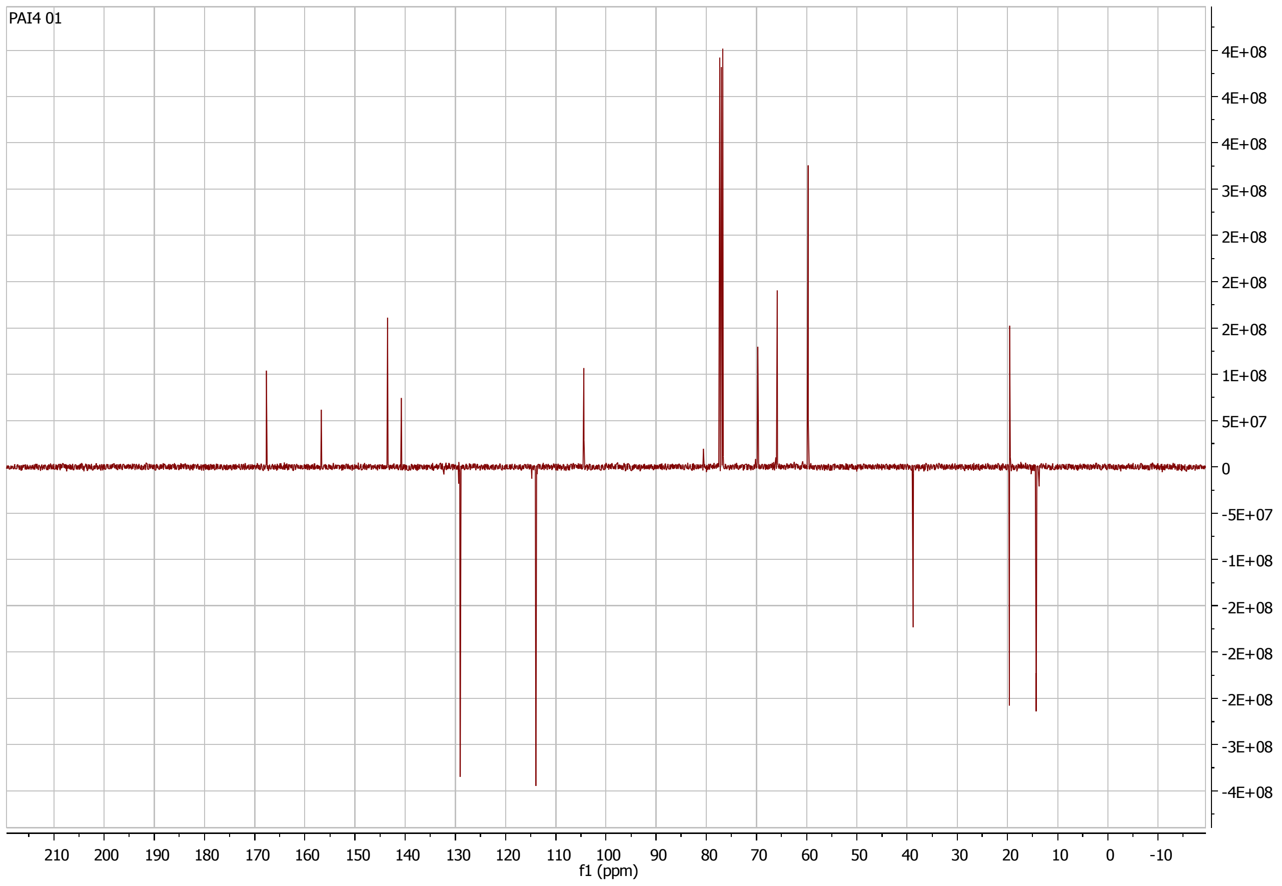

1HNMR spectra of compound 3m

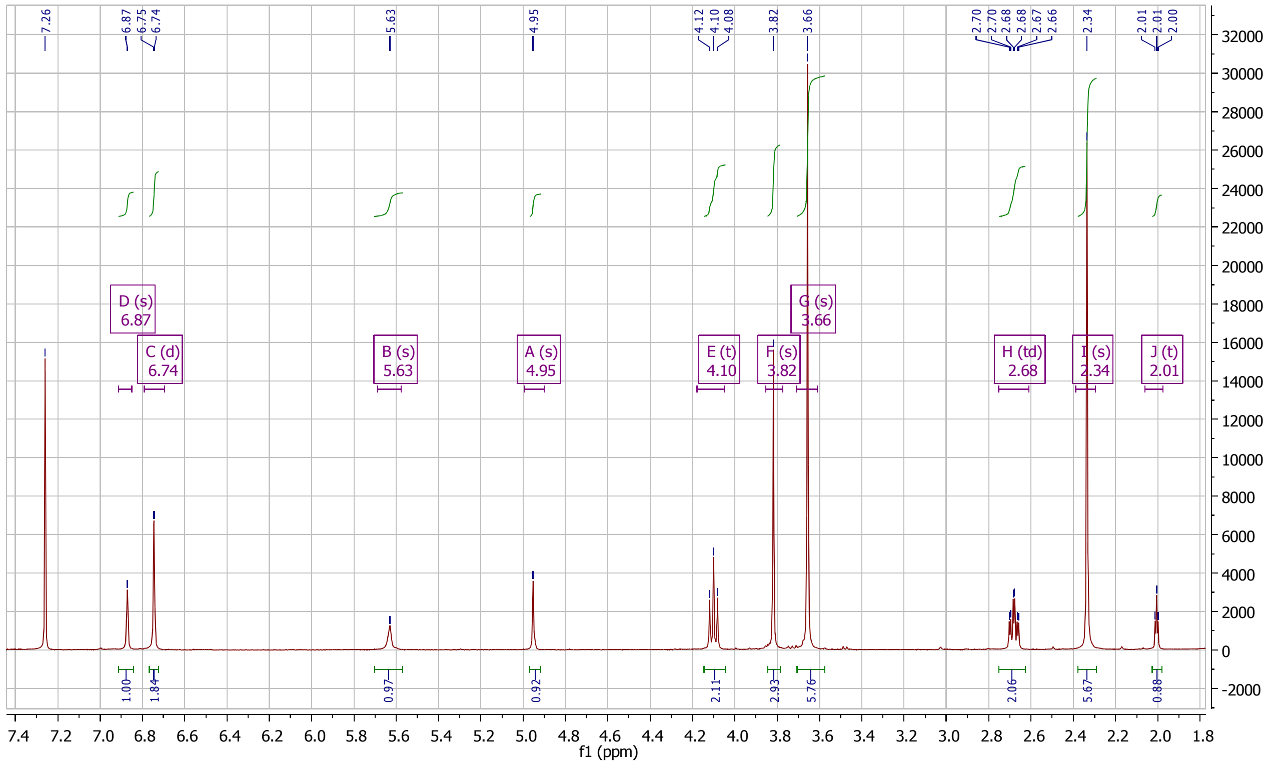

13CNMR spectra of compound 3m

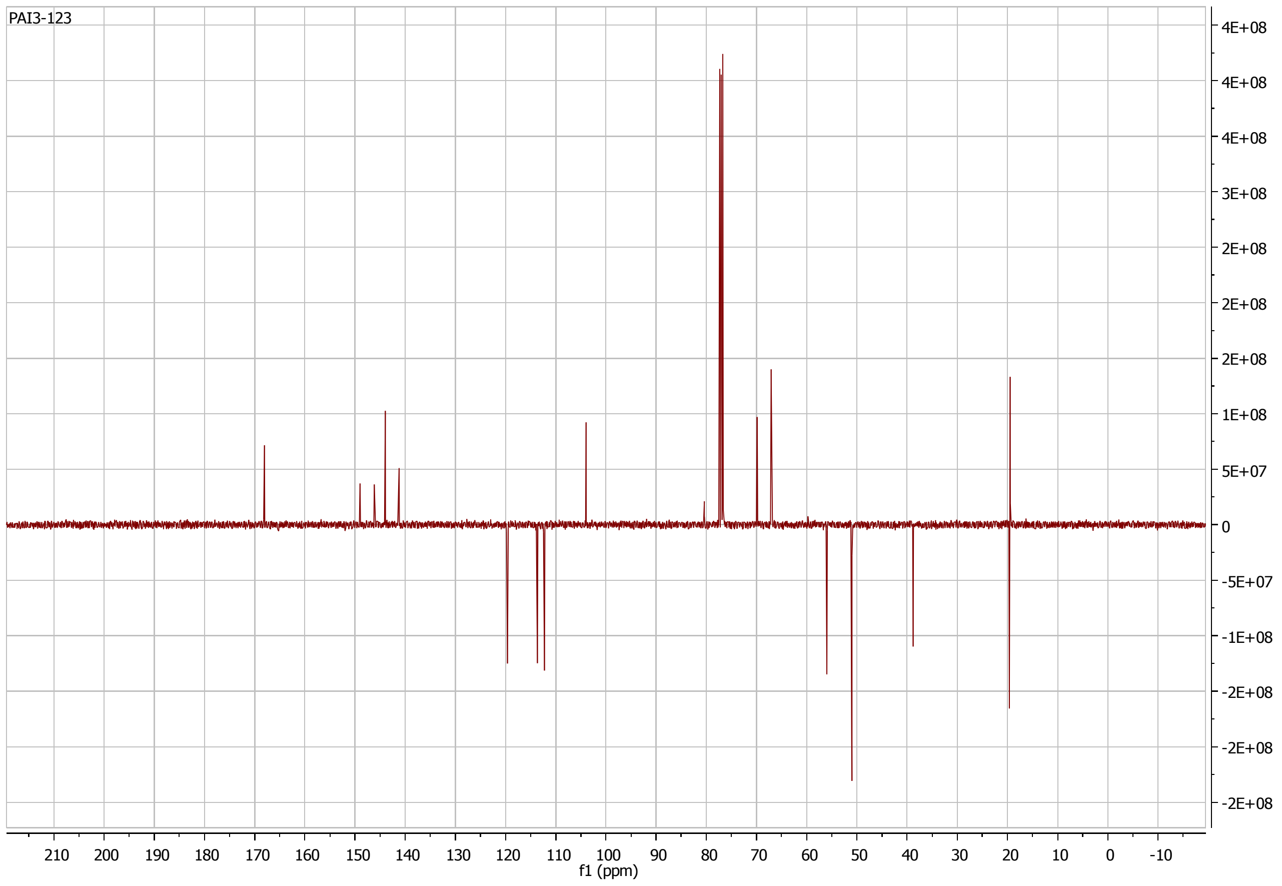

1HNMR spectra of compound 3n

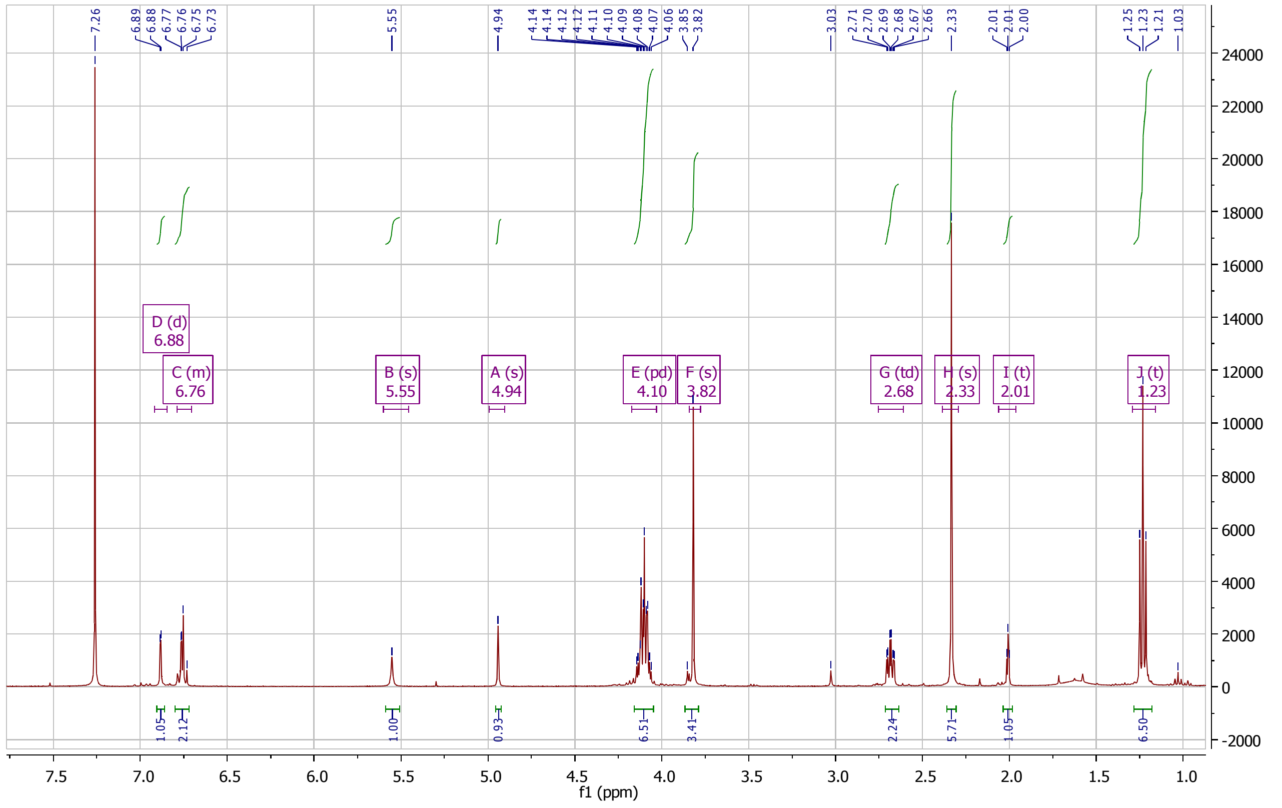

13CNMR spectra of compound 3n

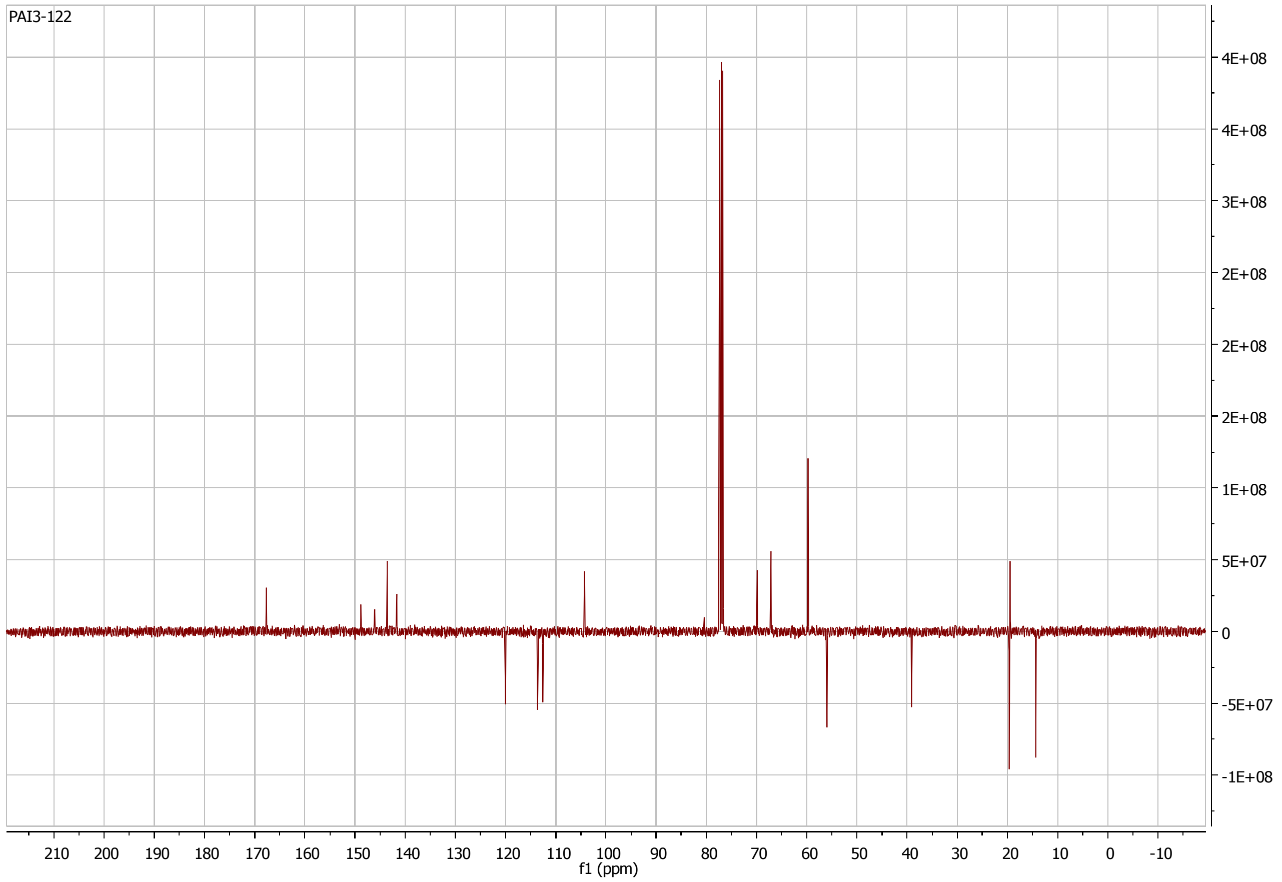

1HNMR spectra of compound 3o

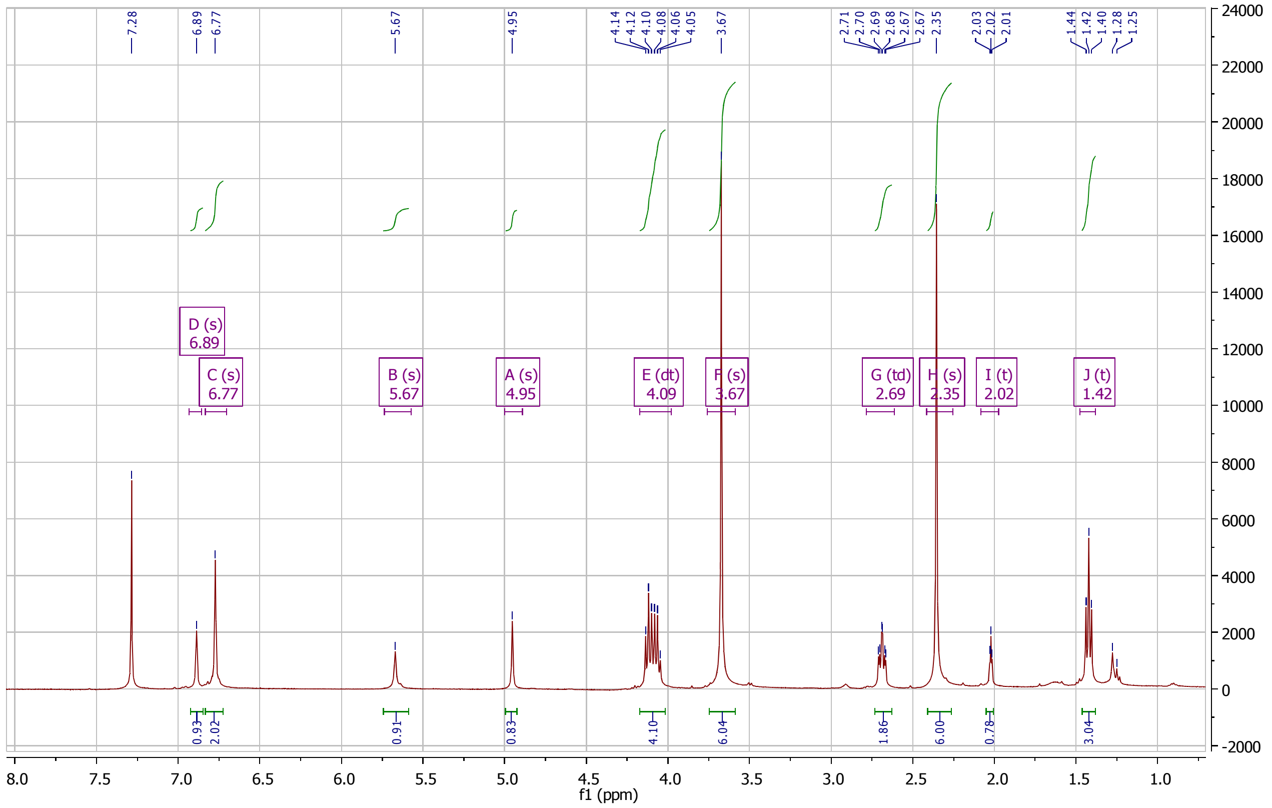

13CNMR spectra of compound 3o

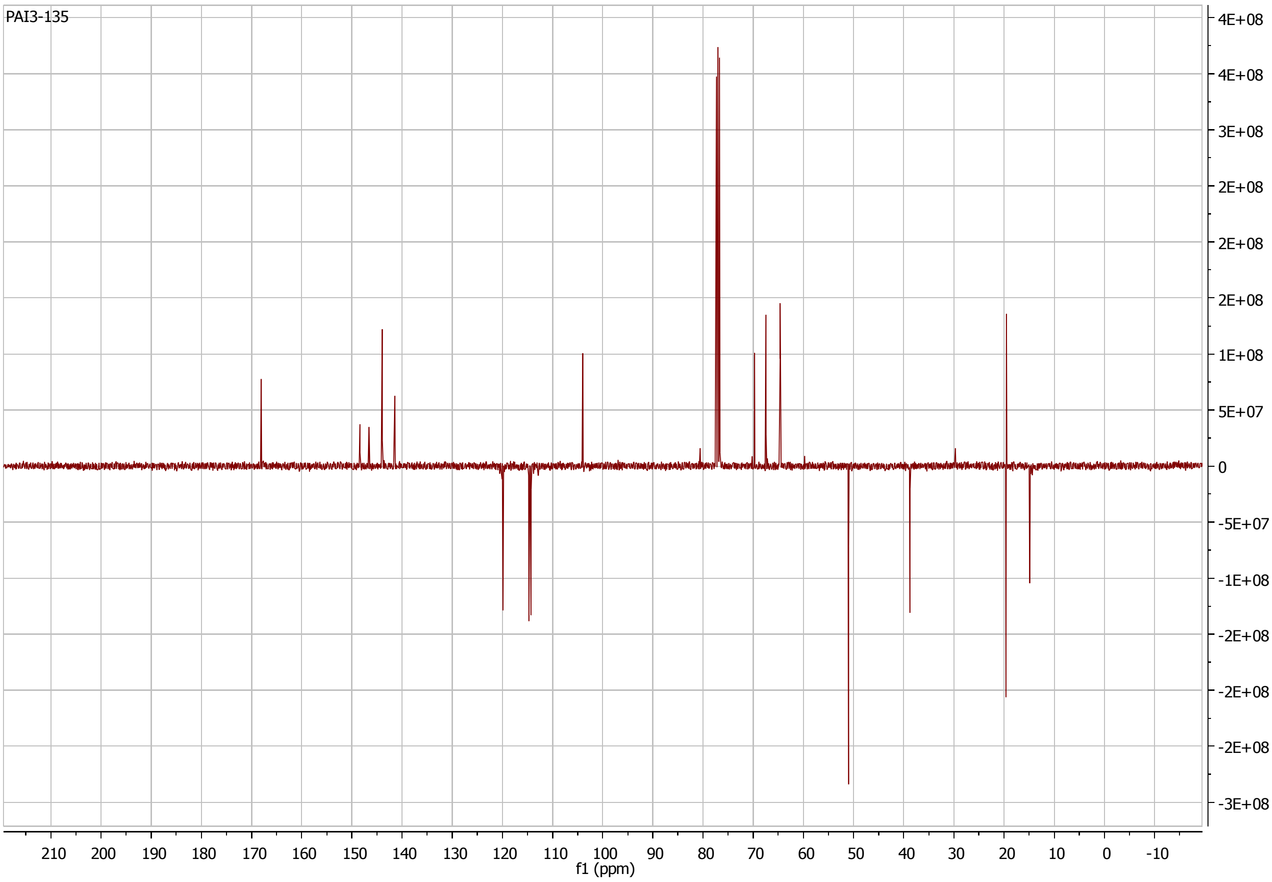

1HNMR spectra of compound 3p

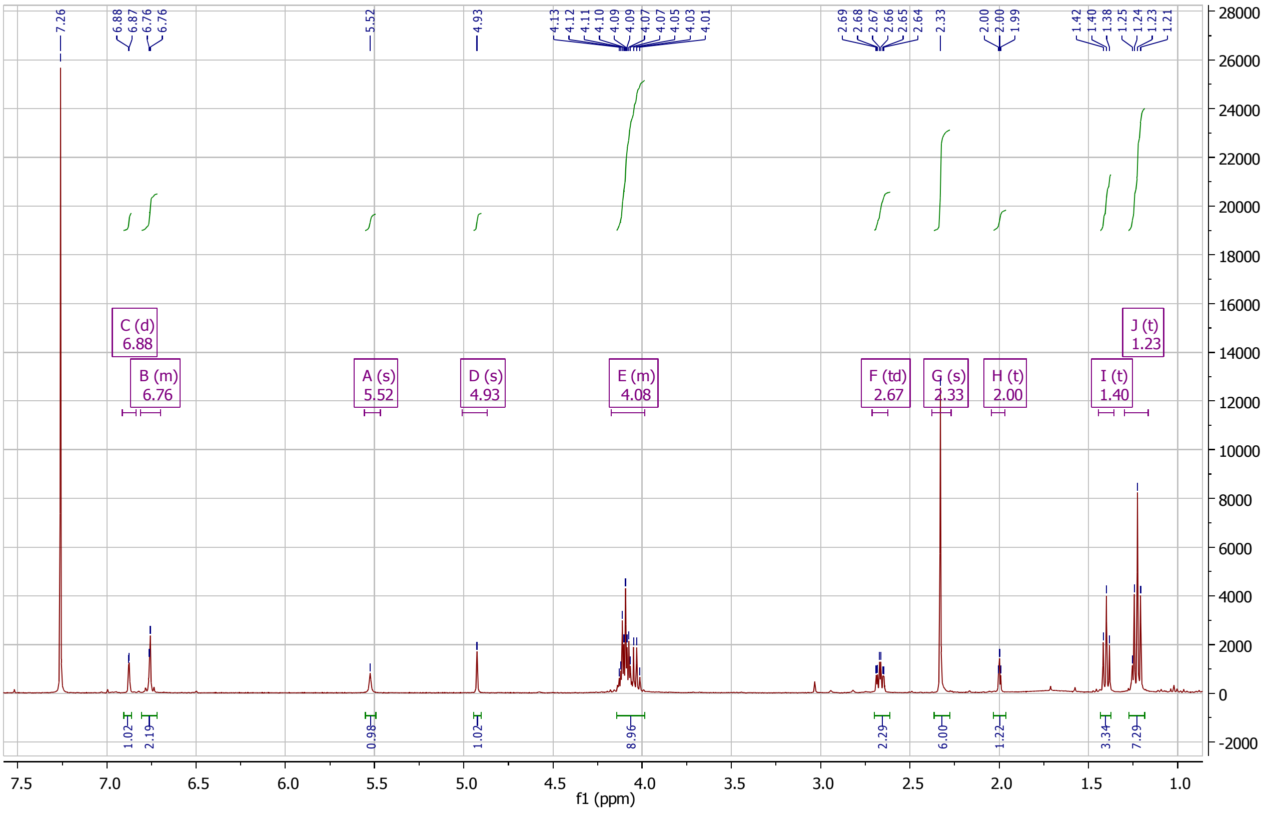

13CNMR spectra of compound 3p

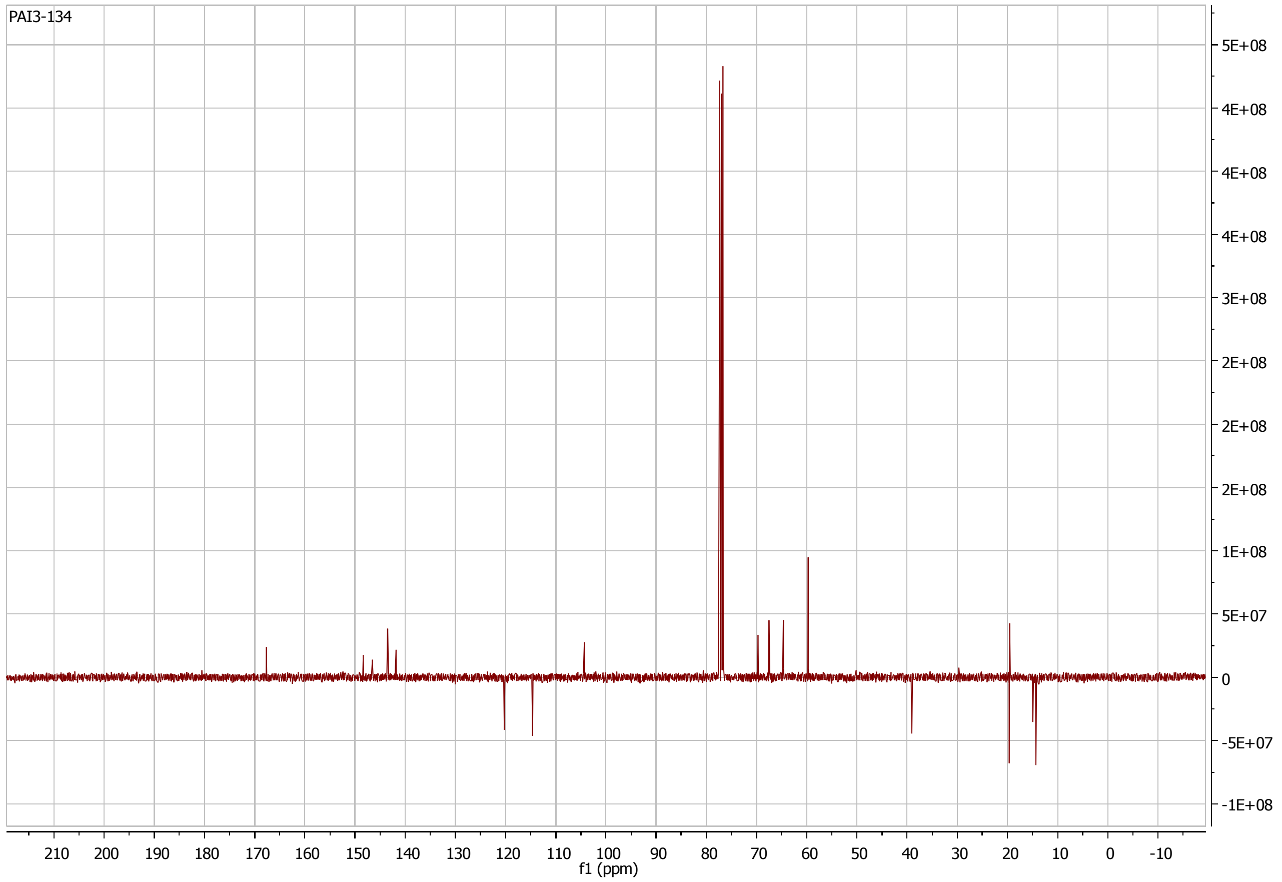

1HNMR spectra of compound 3q

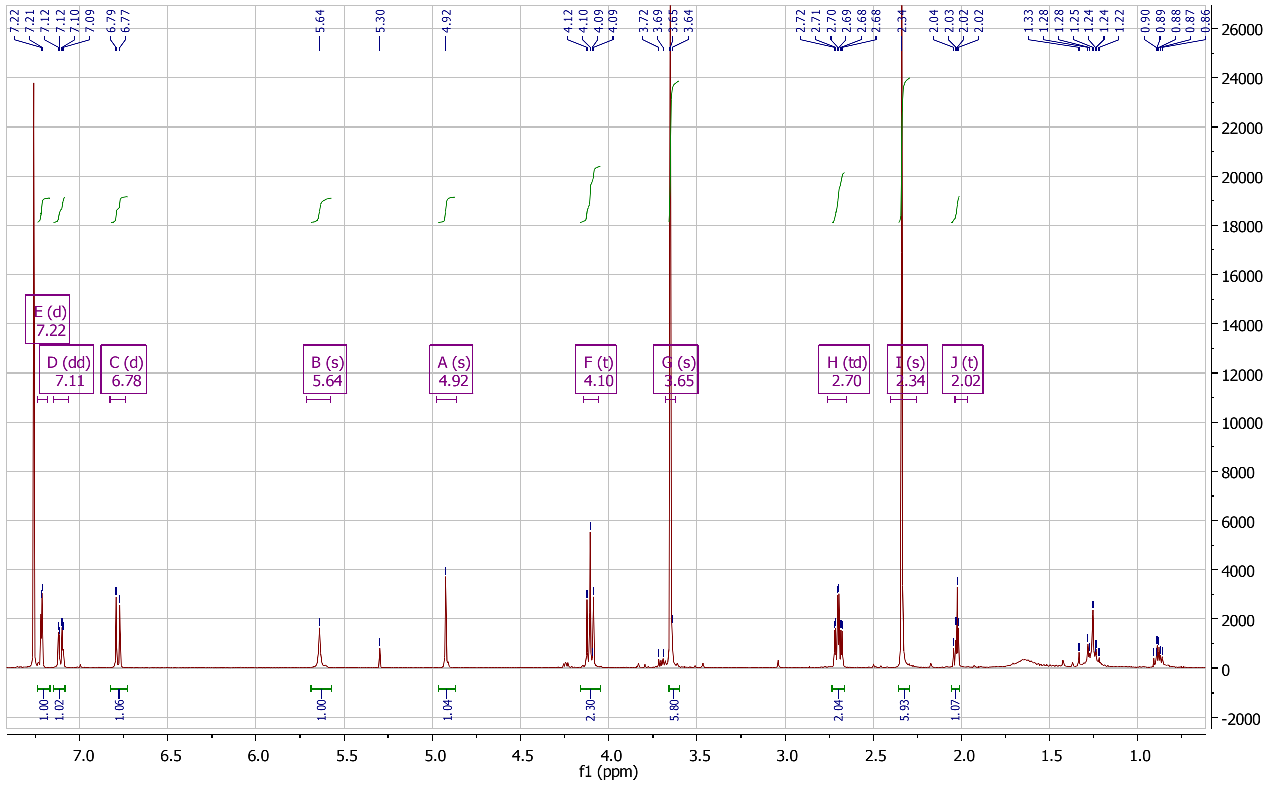

13CNMR spectra of compound 3q

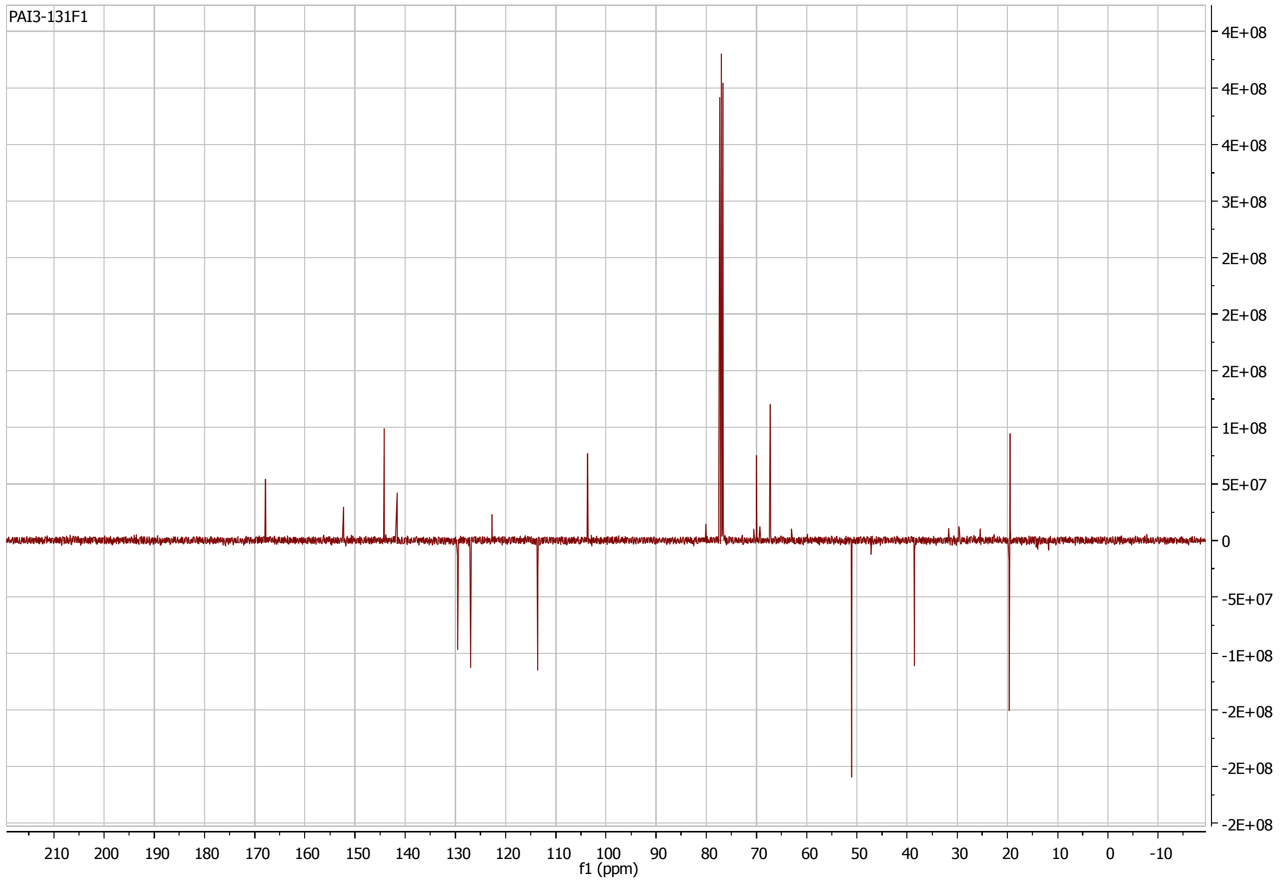

1HNMR spectra of compound 3r

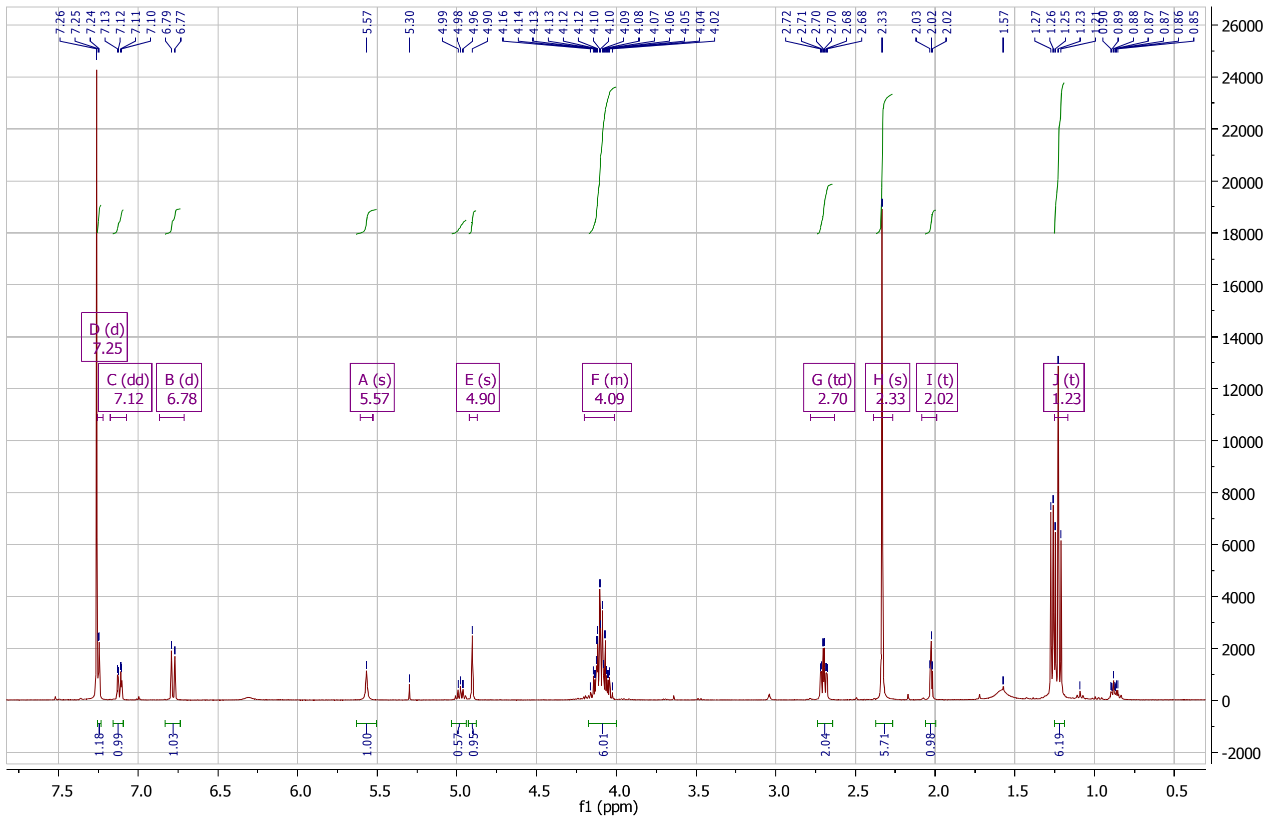

13CNMR spectra of compound 3r

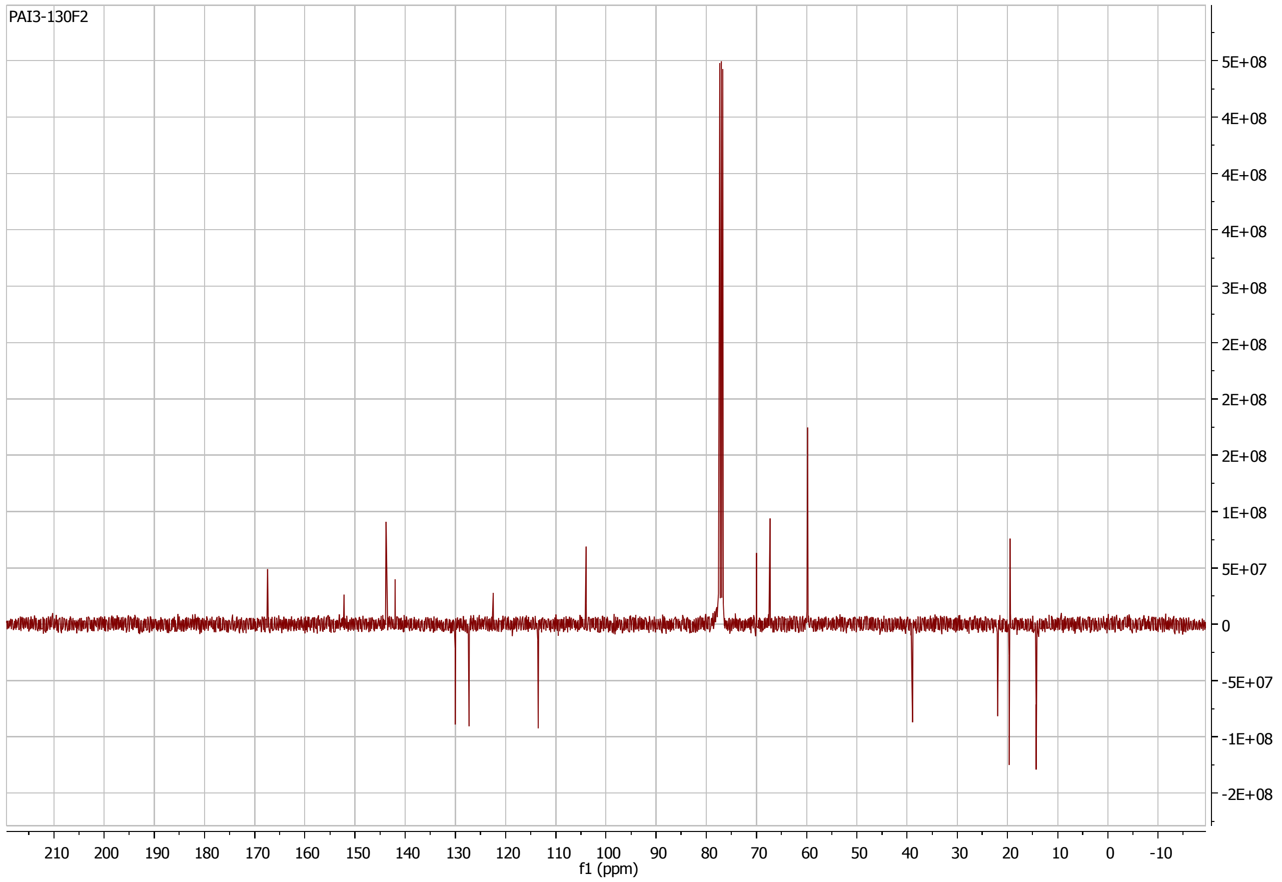

1HNMR spectra of compound 3s

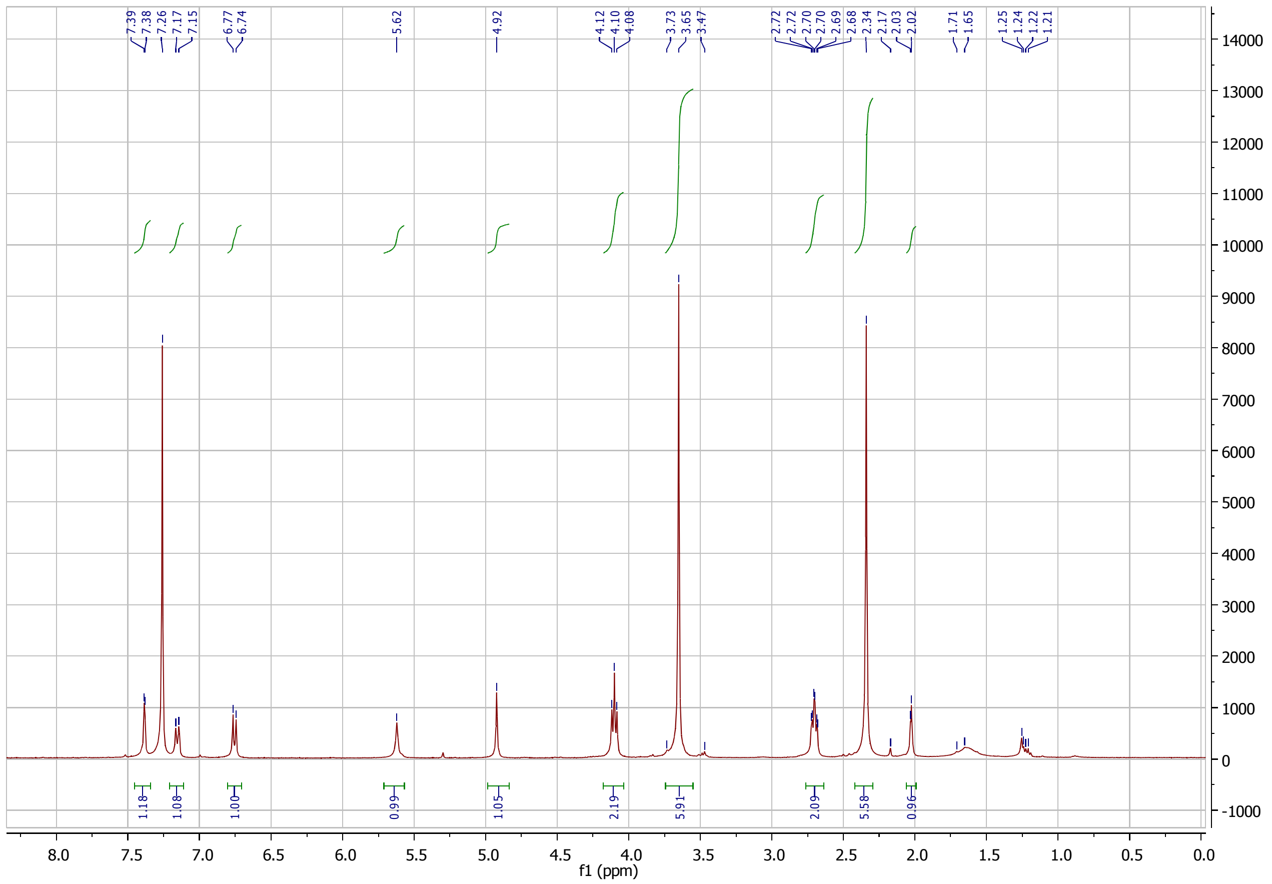

13CNMR spectra of compound 3s

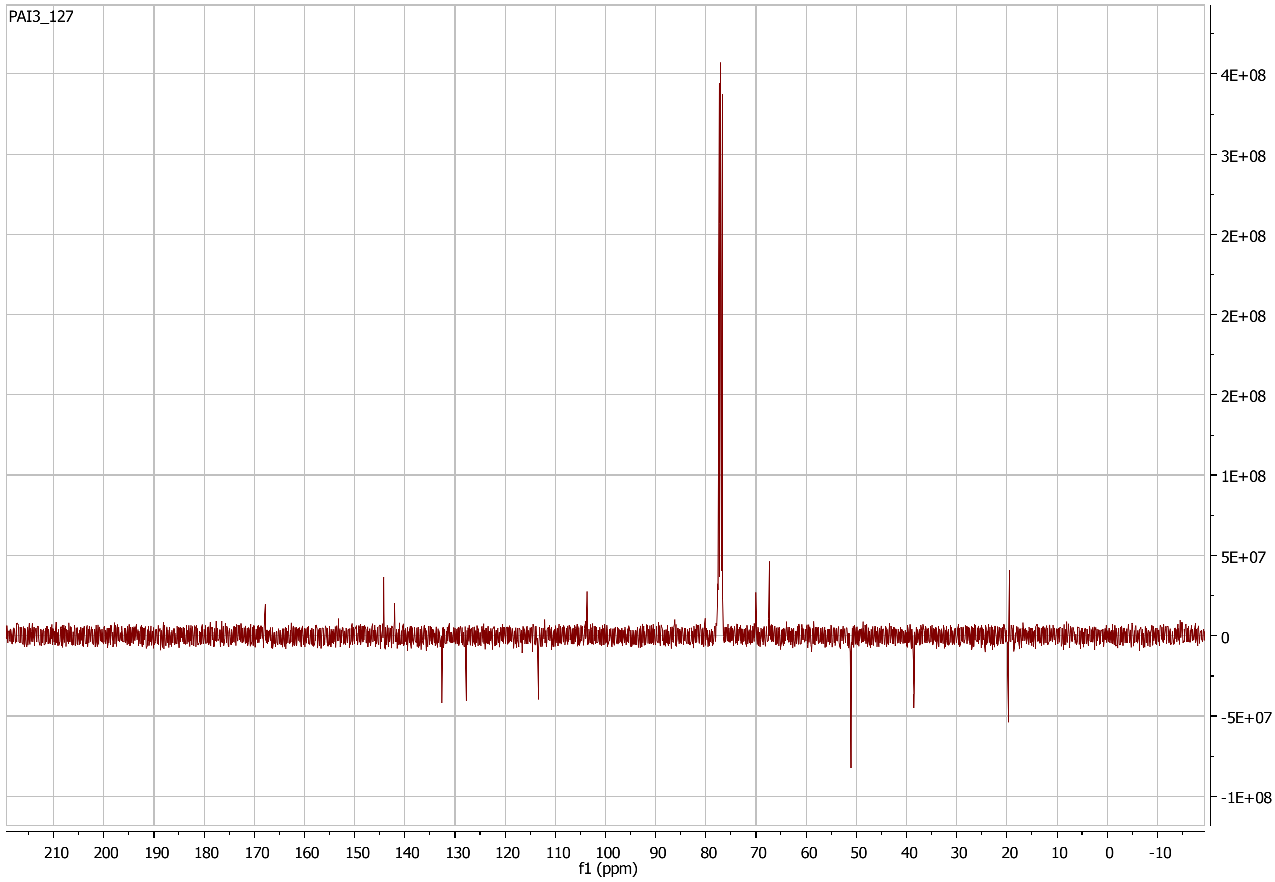

1HNMR spectra of compound 3t

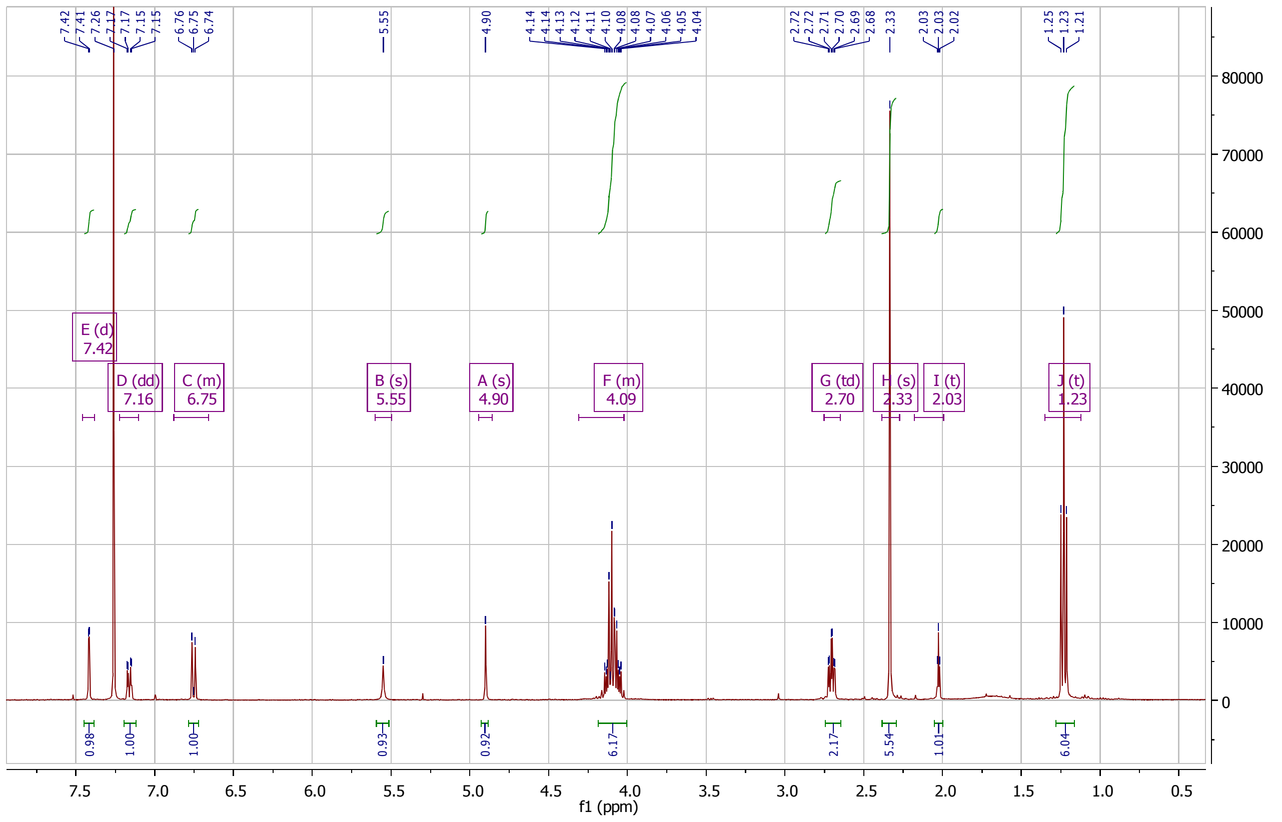

13CNMR spectra of compound 3t

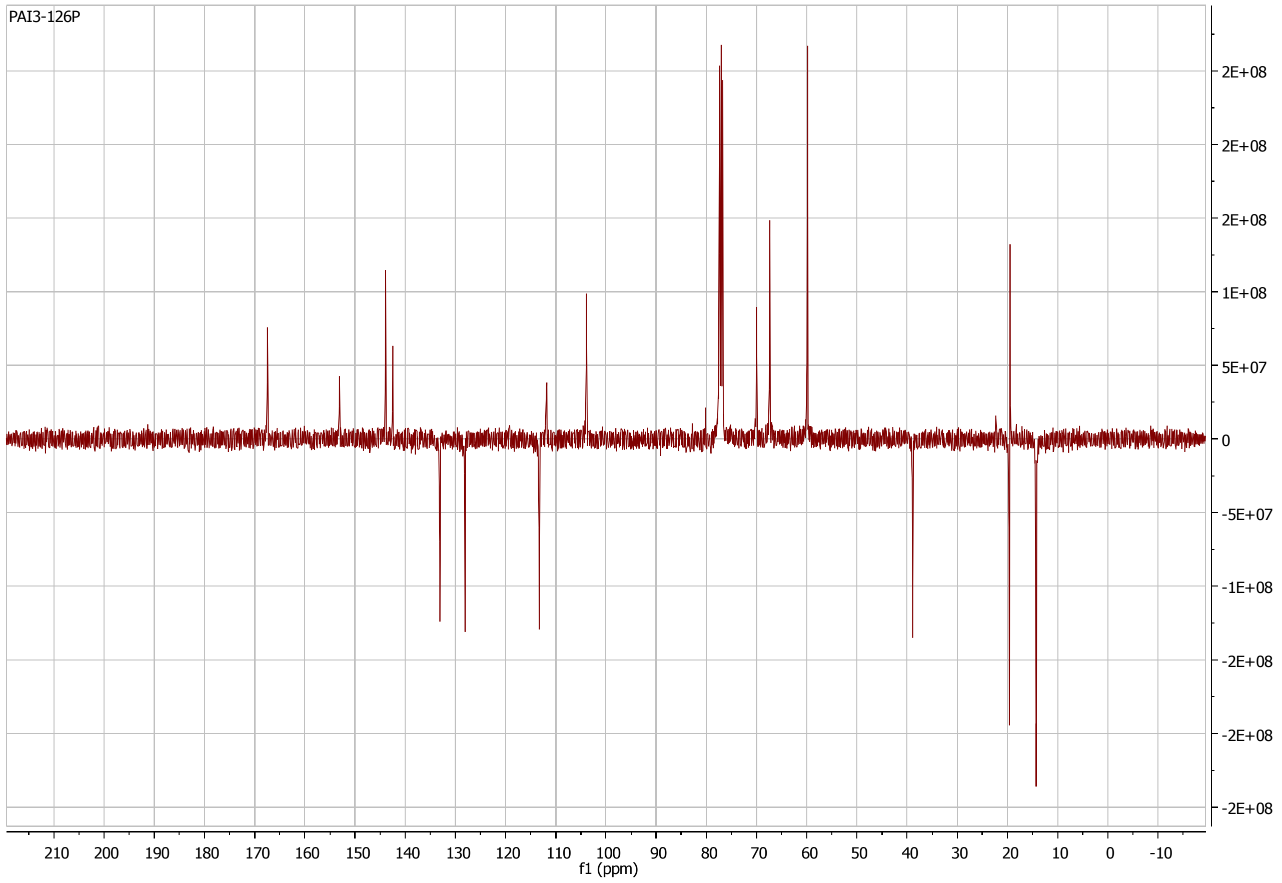

Supplement: Supplementary file 1 [file molecules-25-01329-s001.pdf]
